# Structural basis of phosphatidylinositol 3-kinase C2α function

**DOI:** 10.1038/s41594-022-00730-w

**Published:** 2022-03-07

**Authors:** Wen-Ting Lo, Yingyi Zhang, Oscar Vadas, Yvette Roske, Federico Gulluni, Maria Chiara De Santis, Andreja Vujicic Zagar, Heike Stephanowitz, Emilio Hirsch, Fan Liu, Oliver Daumke, Misha Kudryashev, Volker Haucke

**Affiliations:** 1 Leibniz-Forschungsinstitut für Molekulare Pharmakologie (FMP), Berlin, Germany; 2 Max Planck Institute for Biophysics, Frankfurt am Main, Germany; 3 Buchmann Institute for Molecular Life Sciences, Goethe University, Frankfurt am Main, Germany; 4 University of Geneva, Faculty of Medicine, Geneva, Switzerland; 5 Max Delbrück Centre for Molecular Medicine (MDC), Crystallography, Berlin, Germany; 6 Department of Molecular Biotechnology and Health Sciences, University of Torino, Torino, Italy; 7 University of Geneva, Section of Pharmacy, Geneva, Switzerland; 8 Department of Biology, Chemistry, Pharmacy, Freie Universität Berlin, Berlin, Germany; 9 Present Address: Biological Cryo-EM Center, Hong Kong University of Science and Technology, Clear Water Bay, Kowloon, Hong Kong, China

**Keywords:** X-ray crystallography, Cryoelectron microscopy, Endosomes, Phospholipids, Membrane lipids

## Abstract

Phosphatidylinositol 3-kinase type 2α (PI3KC2α) is an essential member of the structurally unresolved class II PI3K family with crucial functions in lipid signaling, endocytosis, angiogenesis, viral replication, platelet formation and a role in mitosis. The molecular basis of these activities of PI3KC2α is poorly understood. Here, we report high-resolution crystal structures as well as a 4.4-Å cryogenic-electron microscopic (cryo-EM) structure of PI3KC2α in active and inactive conformations. We unravel a coincident mechanism of lipid-induced activation of PI3KC2α at membranes that involves large-scale repositioning of its Ras-binding and lipid-binding distal Phox-homology and C-C2 domains, and can serve as a model for the entire class II PI3K family. Moreover, we describe a PI3KC2α-specific helical bundle domain that underlies its scaffolding function at the mitotic spindle. Our results advance our understanding of PI3K biology and pave the way for the development of specific inhibitors of class II PI3K function with wide applications in biomedicine.

## Abstract (web summary)

High-resolution structures of phosphatidylinositol 3-kinase (PI3K) type IIa unravel a coincident mechanism of lipid-induced enzyme activation and enable the development of inhibitors of class II PI3K function with applications in biomedicine.

## Main

Phosphoinositide 3-kinases (PI3Ks) are a family of lipid-modifying enzymes that phosphorylate the 3′-OH group of inositol phospholipids and play key roles in physiology ranging from cell growth and metabolism to organismal development. Dysfunction of PI3K signaling is implicated in human diseases including cancer, immunodeficiency, diabetes and neurological disorders^1–3^. Mammalian PI3Ks are grouped into three classes based on their structural organization. Class I PI3Ks are receptor-activated heterodimeric enzymes with pivotal roles in cell signaling (for example, cell growth) via synthesis of phosphatidylinositol (PI) 3,4,5-trisphosphate (PI(3,4,5)P_3_) at the plasma membrane^2,4–6^. Isoform-specific pharmacological inhibitors of class I PI3K activity have undergone clinical development as anticancer therapeutics and for the treatment of human disorders caused by PI3K pathway hyperactivation.

Complexes of Vps34, the sole class III PI3K member, produce PI 3-phosphate (PI(3)P) in the endolysosomal system and during autophagy to regulate vesicle-mediated sorting en route to lysosomes^1^. Recent structural studies^7,8^ have enabled the development of selective Vps34 inhibitors that have been instrumental for the analysis and manipulation of class III PI3K function in autophagy and in the regulation of nutrient signaling.

The class II PI3K isoforms PI3KC2α, PI3KC2β and PI3KC2γ are unique in directly synthesizing PI 3,4-bisphosphate (PI(3,4)P_2_) from PI 4-phosphate (PI(4)P) at the plasma membrane and within the endolysosomal system^9,10^, in addition to synthesis of PI 3-phosphate (PI(3)P)^11–14^. The mechanistic basis for the ability of class II PI3Ks to recognize PI(4)P as a substrate to directly produce local pools of PI(3,4)P_2_ at defined endocytic membrane nanodomains is unknown. PI3KC2α is essential in mice^13^. Loss of its catalytic activity is associated with cellular defects in endocytosis^15,16^, angiogenesis and endothelial cell function^17–19^, regulation of blood pressure^20^, viral replication^21,22^, platelet formation^23,24^ and primary cilia signaling^13^. Abrogation of PI3KC2α activity in animal models and in humans leads to kidney cyst formation, skeletal abnormalities, neurological symptoms and cataract formation^25^. In addition to these catalytic roles, PI3KC2α is also required for genome stability by acting as a scaffold at the mitotic spindle during cell division^26^.

In contrast to class I^4,6^ and class III PI3Ks (refs. ^4,7,8,27^) that are understood in structural detail, little is known about the structural and functional architecture, and mechanism of activation of class II PI3Ks including PI3KC2α. Unlike their class I and class III relatives that are targeted to their site of action via associated subunits, class II enzymes such as PI3KC2α lack stable association with other subunits^4,14^ and, thus, must be activated via a distinct regulatory mechanism that so far has remained elusive. The lack of structural information on PI3KC2α and related class II PI3Ks has also greatly hampered the development of isoform-selective pharmacological inhibitors for clinical applications. Moreover, the molecular basis of the scaffolding function of PI3KC2α at the mitotic spindle via its association with the microtubule-binding protein TACC3 at kinetochore fibers to prevent aneuploidy^26^ is unknown.

To address these important unresolved issues, we have determined high-resolution crystal structures as well as a 4.4-Å cryogenic- electron microscopic (cryo-EM) structure of PI3KC2α in different conformational states and in the presence of nonselective small molecule inhibitors. Our results identify a hitherto unknown coincident mechanism of lipid-induced activation of PI3KC2α that is distinct from that of all other PI3Ks and can serve as a model for the entire class II PI3K family. Moreover, our structural and functional biochemical data will greatly facilitate the future development of isoform-selective PI3KC2α inhibitors with biomedical applications that range from the treatment of thrombosis^28^ to viral infections^21,22^, diabetes^29^ and cancer^1,14^.

## Results

### Overall architecture of PI3KC2α

**Fig. 1:**
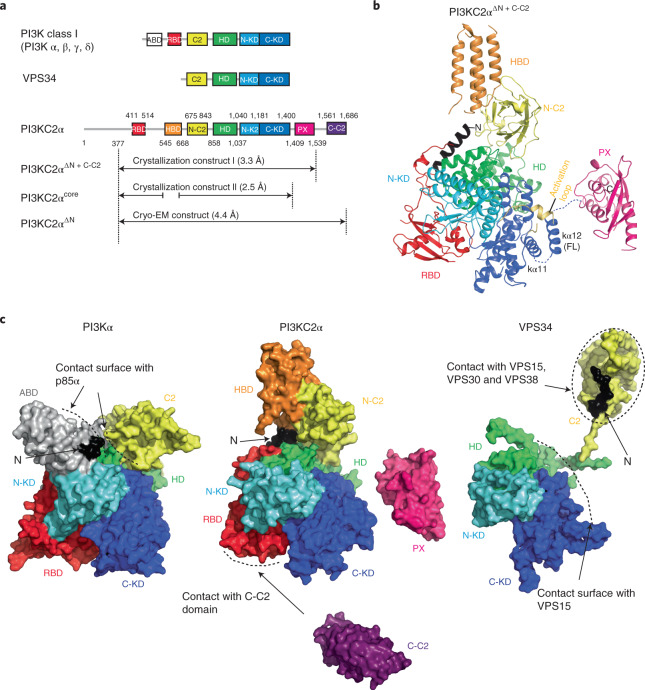
Overall structure of PI3KC2α. **a**, Domain organization of PI3Ks. ABD, adapter binding domain, C2, C2 domain, HD, helical domain, N-KD, C-KD, N- and C-terminal lobes of the kinase core domain. Amino acids 1–377 of PI3KC2α are predicted to be disordered. HBD specific to class II PI3Ks identified in this study. Recombinant PI3KC2α constructs used for X-ray protein crystallography and single-particle cryo-EM are indicated. **b**, Overall structure of PI3KC2α^ΔN+ΔC−C2^. The compact core comprises an N-terminal helix (black), the RBD (red), N-C2 (yellow), HD (green), N-KD (cyan) and C-KD (blue). The HBD (orange) points away from the compact core region and forms the stalk of the inverted lollipop. In this open conformation, a short helix is observed in the activation loop (yellow) and the kα12 helix within the KD is well folded. The N terminus of the PX domain is located 15.6 Å away from the C terminus of KD. **c**, Comparison of PI3K architectures. Surface representations of PI3Kα (PDB 2RD0; that is, a class I PI3K), PI3KC2α^ΔN+ΔC−C2^ (as in **b**) and its C-C2 domain (PDB 6BTY) (that is, a class II PI3K), and Vps34 (PDB 5DFZ; that is, class III PI3K). The HD and N-KD of PI3Kα contact the N-terminal domain of PI3Kα; the p85α regulatory subunit associates with the ABD. PI3KC2α lacks regulatory subunits but contains a unique HBD as well as lipid-binding PX and C-C2 domains that regulate membrane binding and activity. In Vps34 the N-terminal C2 domain acts as a protein interaction hub for its associated subunits Vps15, Vps30 and Vps38.

**Extended Data Fig. 1:**
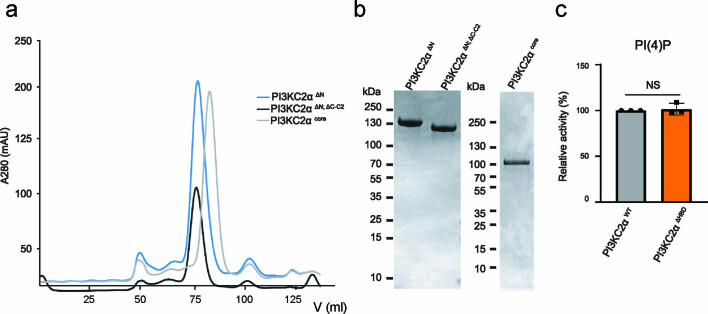
Purification and lipid kinase activity of PI3KC2α^ΔN^, PI3KC2α^ΔN+ΔC−C2^, and PI3KC2α^core^. **(a)** Size exclusion chromatography of PI3KC2α^ΔN^, PI3KC2α^ΔN+ΔC−C2^ and PI3KC2α^core^. Peak fractions were collected for structural analysis. **(b)** Coomassie Blue-stained SDS–PAGE of purified PI3KC2α^ΔN^, PI3KC2α^ΔN+ΔC−C2^ and PI3KC2α^core^ from (a). **(c)** *In vitro* kinase activity of PI3KC2α^WT^ and PI3KC2α^ΔHBD^ using PI(4)P as a substrate. HBD deletion showed no influence on kinase activity. Data represent mean ± SD from triplicate (n = 3) experiments. One sample, 2 tailed t-test with hypothetical mean of 100, NS = non-significant. Source data

**Extended Data Fig. 2:**
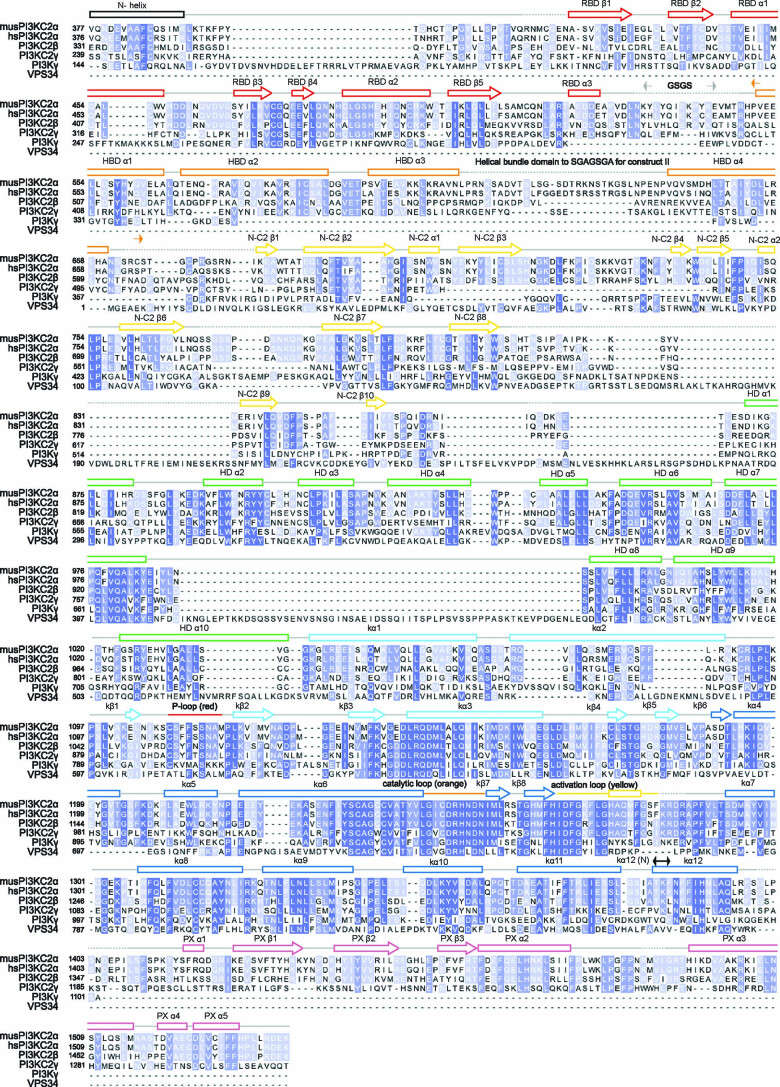
Secondary structure of PI3KC2α and primary sequence alignment with PI3Ks. The sequence corresponding to crystallization construct I (that is musPI3KC2α^ΔN+ΔC−C2^) was used as template to align human PI3KC2α (hs PI3KC2α, UniProt accession: O00443.2), other human class II PI3K isoforms [PI3KC2β (UniProt accession: O00750.2), PI3KC2γ (UniProt accession: O75747.3)], human class I PI3K (PI3Kγ, UniProt accession: P48736.3), and human class III PI3K (VPS34, UniProt accession: Q8NEB9.1). The dashed lines indicate disordered linker regions. All identified secondary structural elements are color-coded as in Fig. 1a. The light grey arrow indicates the replacement of a short, disordered sequence by GSGS to improve crystallization of PI3KC2α^ΔN+ΔC−C2^. To generate the ΔHBD construct, the HBD was replaced by the sequence SGAGSGA as indicated by the orange arrow. kα12 (N) is indicated by a black arrow.

Like other members of the PI3K family^1,4^, PI3KC2α contains a PI3K core that consists of a Ras-binding domain (RBD) and an N-terminal C2 domain, as well as helical and kinase domains (KDs). In addition, PI3KC2α harbors the C-terminal Phox-homology (PX) and C2 domains that are unique to class II enzymes (Fig. 1a). To determine the X-ray crystal structure of PI3KC2α, we embarked on an iterative process of construct screening and optimization of various forms of PI3KC2α from different species assisted by hydrogen/deuterium exchange–mass spectrometry (HDX–MS) to identify disordered regions. A purified mouse PI3KC2α construct containing a re-engineered internal loop sequence and lacking the intrinsically disordered N-terminal region and the C-terminal C2 domain (C-C2) (Extended Data Figs. 1a,b and 2) was used to obtain the 3.3 Å crystal structure of PI3KC2α^ΔN+ΔC−C2^ (Table 1).

**Table 1:** Data collection and refinement statistics

	PI3KC2α^ΔN+C−C2^	PI3KC2α^core^ (apo)	PI3KC2α^core^ (ATP-Mg^2+^)	PI3KC2α^core^ (Torin-2)	PI3KC2α^core^ (PIK-90)
**Data collection**^a^					
Space group	*P*2_1_2_1_2_1_	*P*2_1_2_1_2_1_	*P*2_1_2_1_2_1_	*P*2_1_2_1_2_1_	*P*2_1_2_1_2_1_
Cell dimensions					
*a*, *b*, *c* (Å)	82.6, 115.9, 144.4	135.2, 151.6, 56.0	56.1, 133.4, 152.7	55.3, 134.2, 151.0	55.9, 135.4, 151.6
α, β, γ (°)	90.0, 90.0, 90.0	90.0, 90.0, 90.0	90.0, 90.0, 90.0	90.0, 90.0, 90.0	90.0, 90.0, 90.0
Resolution (Å)	49.20–3.25 (3.45–3.25)^b^	43.20–2.42 (2.51–2.42)	47.56–2.75 (2.85–2.75)	47.49–2.59 (2.68–2.59)	42.26–2.65 (2.75–2.65)
*R*_merge_	0.14 (2.42)	0.11 (2.23)	0.13 (1.71)	0.10 (2.13)	0.11 (2.10)
*I* / σ*I*	9.73 (0.84)	10.56 (0.60)	9.58 (0.77)	10.91 (0.67)	11.17 (0.72)
Completeness (%)	99.1 (97.1)	99.6 (99.0)	99.5 (99.4)	97.6 (93.4)	97.6 (96.2)
Redundancy	4.9 (4.9)	4.5 (4.4)	4.2 (4.1)	4.1(3.8)	4.0 (3.8)
**Refinement**					
Resolution (Å)	3.25	2.42	2.75	2.59	2.65
No. reflections	22,257	44,389	30,372	34,906	33,051
*R*_work_ / *R*_free_	26.16 / 30.85	22.93 / 27.68	24.59 / 28.65	21.95 / 26.87	23.39 / 27.69
No. atoms					
Protein	8,224	6,552	6,611	6,519	6,540
Ligand/ion	41	13	50	65	46
Water	19	85	86	79	56
*B* factors					
Protein	136.62	72.15	82.14	86.56	80.84
Ligand/ion	231.63	106.22	100.98	118.78	98.83
Water	139.07	62.28	59.11	74.12	61.86
R.m.s. deviations					
Bond lengths (Å)	0.013	0.009	0.014	0.004	0.013
Bond angles (°)	1.63	1.25	1.66	0.69	1.67

^a^One crystal for each structure was used for data collection and structure determination.

^b^Values in parentheses are for the highest-resolution shell.

The overall structure of PI3KC2α^ΔN+ΔC−C2^ comprises a large compact multidomain kinase core that is mounted onto a helical stalk (Fig. 1b). The latter is unique to class II PI3Ks and has not been observed or predicted before in any other PI3K, that is PI3Kα−δ or Vps34 (Fig. 1c). The compact core region is flanked by the distantly located PX domain that is stabilized by contacts with a neighboring molecule in the crystal. Earlier biochemical data demonstrated an autoinhibitory role of the PX-C2 domains for PI3KC2α catalytic activity^30^. The release of the PX-C2 domain from the catalytic core may thus indicate that the obtained PI3KC2α^ΔN+ΔC−C2^ crystal structure represents an active conformation of the enzyme, a hypothesis tested and corroborated below. The core region and the helical stalk comprise an N-terminal helix and five additional domains: the RBD, the N-terminal C2 domain (N-C2), the solenoid helical domain that tightly packs against the catalytic KD comprising a smaller N-terminal and a larger C-terminal lobe, and a helical bundle domain (HBD) (Fig. 1b). The helical domain in this assembly acts as the central hub that connects the conserved archetypical two-lobed KD of PI3KC2α (refs. ^4,6,31^) with the N-C2 domain. The globular N-terminal RBD displays a conserved α/β sandwich fold that resembles the RBD of class I PI3Ks (refs. ^4,6^) (Fig. 1c) and forms close contacts with the N-terminal lobe of the KD. In spite of this structural conservation, the primary sequence between the RBDs of PI3KC2α and class I PI3Ks is poorly conserved (<25%, Extended Data Fig. 2). This is consistent with the fact that class II PI3Ks including PI3KC2α functionally associate with endosomal Rab family proteins^12,13^ rather than with Ras, a major activator of class I PI3Kα (refs. ^5,6,32^).

A conserved feature of all mammalian class II PI3Ks is a sequence insertion of about 100 amino acids between the RBD and the N-C2 domain (Extended Data Fig. 2) that is absent in class I and class III enzymes (Fig. 1c). This sequence folds into a unique stalk-forming HBD comprising four helices (Fig. 1b). The HBD points away from the kinase core and is dispensable for catalytic activity (Extended Data Fig. 1c), suggesting that it exhibits a structural role as a protein interaction scaffold, as corroborated below (Fig. 6). The HBD contacts the N-terminal C2 domain via its C-terminal helices and connects to this domain via a short flexible linker. The N-C2 domain preserves an antiparallel β-sandwich topology. Based on the abundance of conserved basic residues within the disordered loop that connects β-strands 3 and 4, the N-C2 domain might associate with negatively charged membrane phospholipids. This role of the N-C2 domain is distinct from its function in Vps34, where it serves as a protein interaction hub^7^ (Fig. 1c).

### Structural basis for PI3KC2α catalytic activity

**Fig. 2:**
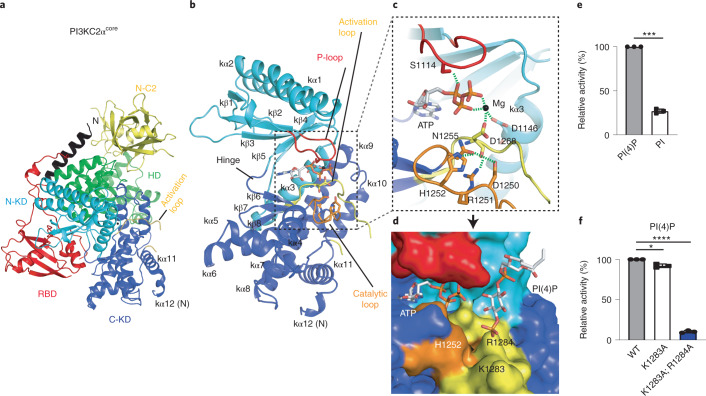
Structure and activity of the PI3KC2α kinase core domain. **a**, Overall structure of PI3KC2α^core^. Note that in this conformation, the short helix within the activation loop is disordered and only the N-terminal part of kα12 is folded. **b**, The kinase core domain (KD) of PI3KC2α in complex with ATP and Mg^2+^. P-loop (red), catalytic loop (orange) and activation loop (yellow) are well defined. ATP is found at the interface between the N and C lobes of the KD. **c**, Close-up view of the ATP-binding pocket. Green dashed lines indicate interactions. The Mg^2+^ ion (gray sphere) neutralizes the charges of D1146 and D1268 to enable phosphate binding of ATP (shown as sticks). R1251 in the catalytic loop interacts with the activation loop. Intramolecular interactions of D1250, H1252 and N1255 in the catalytic loop are identified. **d**, Model for diC4-PI(4)P binding to the KD of PI3KC2α. Surface representation of the KD with K1283 and R1284 in the activation loop contacting the 4-phosphate of PI(4)P. **e**, In vitro kinase activity of PI3KC2α^ΔN^ using either PI(4)P or PI as a substrate. Data represent mean ± s.d. from *n* = 3 experiments, one sample, two-tailed *t*-test with hypothetical mean of 100, ^∗∗∗^*P* = 0.0003. **f**, PI(3,4)P_2_-synthesizing activity of PI3KC2α^ΔN^ activation loop mutants. Mutation of K1283A and K1284A abrogates kinase activity. Data represent mean ± s.d. from *n* = 3 experiments, One sample, two-tailed *t*-test with hypothetical mean of 100, ^∗^*P* = 0.0263 and ^∗∗∗∗^*P* < 0.0001. Source data

**Extended Data Fig. 3:**
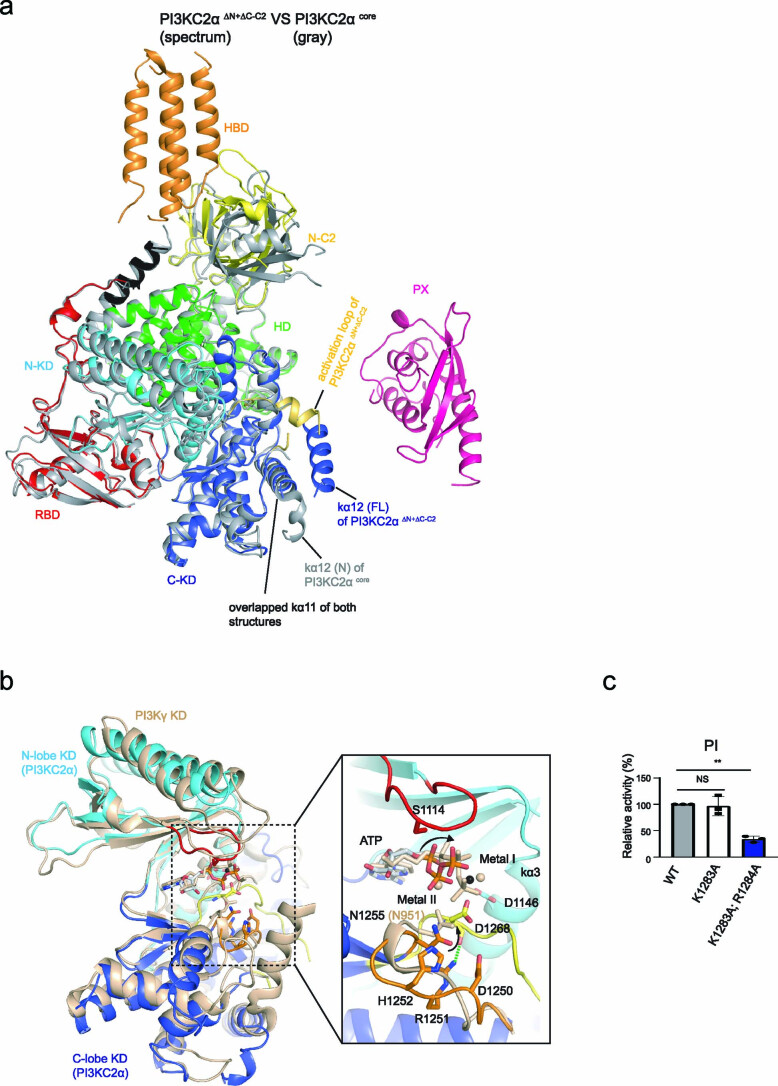
Catalytic activity and structure of PI3KC2α. **(a)** Structural superimposition of PI3KC2α^ΔN+ΔC−C2^ (spectrum) and PI3KC2α^core^ (gray). The main distinctive features are the conformations of kα12 and of the activation loop. In the PI3KC2α^ΔN+ΔC−C2^ structure, a short helical segment within the activation loop and kα12 is well defined and folded. In contrast, in the structure of PI3KC2α^core^, only the N-terminal part of kα12 is folded and the short helical segment within the activation loop of PI3KC2α ^core^ is disordered. **(b)** Structural superimposition of the kinase domains from PI3KC2α (N-lobe: cyan; C-lobe: blue) and PI3Kγ (1E8X; wheat). Close-up view of the proposed movement of catalytic loop residues, N1255, and ATP with the appearance of a second metal ion (metal II). Wheat spheres indicate two Mg^2+^ ions bound to PI3Kγ, black sphere represents the single Mg^2+^ bound to PI3KC2α. **(c)** *In vitro* kinase activity of activation loop mutants with PI as a substrate. The K1283A and R1284A mutations greatly reduce activity. Data represent mean ± SD from triplicate (n = 3) experiments. One sample, 2 tailed t-test with hypothetical mean of 100, NS = non-significant; ^∗∗^p = 0.0025. Source data

To gain insights into the mechanism of catalysis and to enable the development of small molecule inhibitors of PI3KC2α, we determined crystal structures of the kinase core domain. Based on the PI3KC2α^ΔN+ΔC−C2^ construct, we designed a recombinant PI3KC2α^core^ variant that lacks the N-terminal low complexity region and the distal PX and C-C2 domains. The HBD was substituted by a short seven residue-long linker (Fig. 1a and Extended Data Fig. 2). The 2.5-Å crystal structure of PI3KC2α^core^ determined in the absence or presence of Mg^2+^ and ATP confirmed the architecture of the PI3KC2α catalytic core (Fig. 2a, Extended Data Fig. 3a and Table 1) and revealed important insights into the catalytic mechanism (Fig. 2b,c).

The PI3KC2α KD displays a typical PI3K KD fold comprising a smaller N- and a larger C-lobe linked by a hinge (Extended Data Fig. 3b). The ATP-binding P-loop is located between strands kβ1 and kβ2, and contacts the α-phosphate via a conserved serine (S1114). The catalytic loop located between kα6 and kβ7 contains a conserved ^1250^DRH^1252^ motif, in which R1251 stabilizes the N-terminal part of the activation loop containing the ^1268^DFG^1270^ motif. H1252 within the DRH motif could act as catalytic base in lipid kinases to deprotonate the 3′-OH of the inositol substrate, a reaction aided by D1250 and D1268 (ref. ^31^). An atypical feature of the crystallized PI3KC2α catalytic core is the presence of only a single Mg^2+^ ion that is coordinated by the β- and γ-phosphates of ATP and D1146 in kα3 and D1268 in the DFG motif. In this Mg^2+^-bound state, N1255, that is a residue bound to a second Mg^2+^ ion in PI3Kγ^5^ and other PI3Ks^4^, interacts with D1250 and H1252 in the HRD motif, apparently to inhibit the catalytic function (Fig. 2c and Extended Data Fig. 3b). Hence, this conformation of PI3KC2α likely reflects an early precatalytic state. Binding of the second Mg^2+^ conceivably induces a conformational change of N1255 to release the HRD motif from intramolecular inhibition, thereby enabling catalysis.

Class II PI3Ks including PI3KC2α among other features are distinguished from class I and class III enzymes by their substrate selectivity, most notably their unique ability to use PI(4)P as a substrate to directly produce PI(3,4)P_2_ at endocytic membranes^1,14^, for example to facilitate endocytosis^15,33^. We confirmed the preference of purified recombinant PI3KC2α to synthesize PI(3,4)P_2_ from PI(4)P over PI(3)P synthesis from PI (Fig. 2e), in agreement with our earlier cell-based data^15,16^. Cellular production of PI(3,4)P_2_ versus PI(3)P in addition to substrate availability is likely modulated by the specific membrane environment, explaining the distinct functions of PI3KC2α at the plasma membrane and at endosomes^1,14^. Lipid substrate binding in PI 3-kinases is encoded within the activation loop. The primary sequence of the activation loop is highly conserved also among class II PI3Ks, suggesting that they bind their substrates via similar mechanisms (Extended Data Fig. 2). While the N-terminal part of the activation loop harboring the catalytic DFG motif is clearly resolved, the C-terminal part of the PI3KC2α activation loop containing the phospholipid headgroup-binding basic residues remains disordered. We therefore used the class I PI3Kα complexed to PI(4,5)P_2_ (ref. ^34^) as a reference to model PI(4)P headgroup binding to PI3KC2α. In this model, the 3′-OH group of inositol is oriented toward the γ-phosphate of ATP, whereas the 4-phosphate binds to a flexible positively charged surface created by K1283 and R1284 on the C-terminal activation loop (Fig. 2d). We tested this model experimentally by creating charge neutralization mutants of PI3KC2α. Consistently, we found that alanine substitution of K1283 and R1284 abrogated catalytic activity toward PI(4)P (Fig. 2f) and greatly reduced phosphorylation of PI (Extended Data Fig. 3c), whereas alanine substitution of K1283 only had very minor effects.

These data provide a firm structural basis for the unique ability of PI3KC2α and related class II PI3Ks to directly synthesize PI(3,4)P_2_ from PI(4)P to control its biological function and activity.

### Small molecule inhibition of PI3KC2α catalytic function

**Fig. 3:**
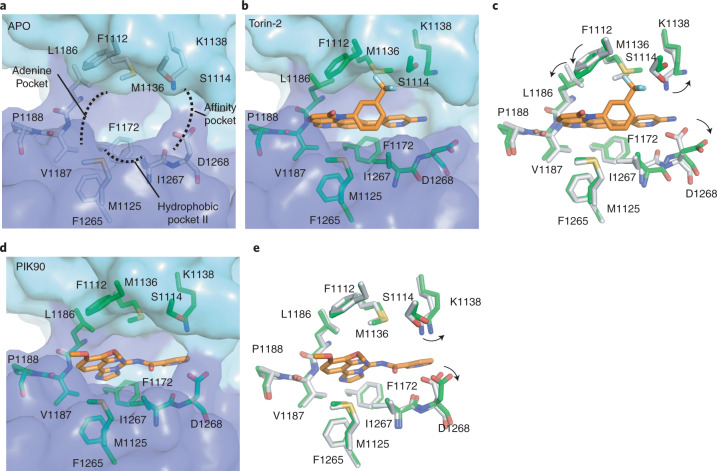
Structural basis for pharmacological inhibition of PI3KC2α by Torin-2 and PIK-90. **a**, The ATP-binding site of the PI3KC2α apo-enzyme. The adenine pocket, affinity pocket and hydrophobic pocket II are indicated with dashed line areas. **b**, Torin-2 binding to the ATP-binding site. The benzonapththyridine ring occupies the adenine pocket and hydrophobic pocket II. The inner amino-pyrimidine group targets the affinity pocket. The benzotrifluoride group binds to the N-lobe of the KD (cyan). **c**, Comparison of the binding pocket of the apo-enzyme (gray) and the PI3KC2α-Torin-2 complex (green). Arrows indicate conformational changes of K1138, D1268, F1112 and L1186 elicited by Torin-2 binding. **d**, PIK-90 binding to the ATP-binding site. The imidazoquinazoline ring of PIK-90 occupies the adenine pocket and hydrophobic pocket II. The terminal pyridine ring binds to the inner surface of the affinity pocket. **e**, Comparison of the binding pocket of the apo-enzyme (gray) and the PI3KC2α-PIK-90 complex (green). Arrows indicate conformational changes to the inhibitor binding. PIK-90 induces comparably minor conformational changes of the binding pocket.

**Extended Data Fig. 4:**
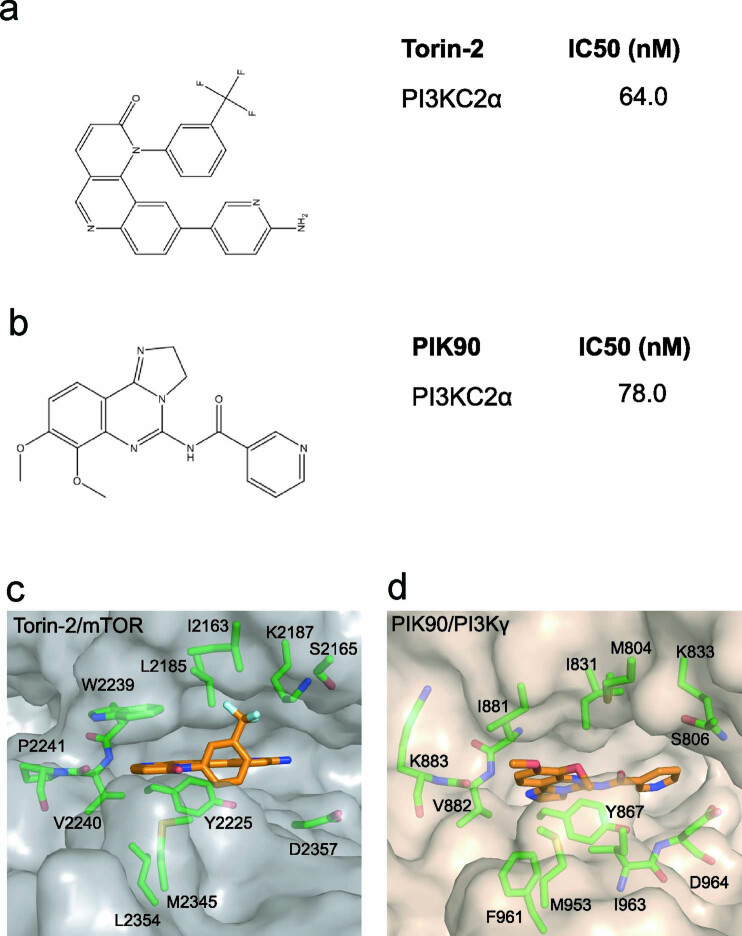
Pharmacological inhibition of mTOR kinase and PI3Kγ by Torin-2 and PIK-90. **(a,b)** 2D structure and IC_50_ of Torin-2 (a) and PIK-90 (b) bound to PI3KC2α. **(c)** Binding pocket of mTOR in association with Torin-2 (pdb: 4JSX). W2239 from the hinge of mTOR kinase contributes a pi-pi interaction with Torin-2. **(d)** Binding pocket of PI3Kγ in complex with PIK-90 (pdb: 2CHX). The pyridine ring of PIK-90 binds to a deep cavity in the affinity pocket of PI3Kγ.

Given the multiple important roles of PI3KC2α and related class II PI3Ks in cell physiology and in disease^1,14^, we next sought to probe the structural basis for inhibition of its catalytic activity by small molecules. Although no specific inhibitors of PI3KC2α have been identified so far, the enzyme is known to be targeted by pan-PI 3-kinase inhibitors (that is, PIK-90, Extended Data Fig. 4b)^35^ and by Torin-2, an ATP-competitive inhibitor of the PI3K-related kinase superfamily member mTOR^36^ (Extended Data Fig. 4a). To explore the determinants of inhibitor potency and specificity, we determined the structures of PI3KC2α^core^ bound to Torin-2 or PIK-90 and compared these to the structure of the apo-form of the enzyme (Fig. 3a). Torin-2 binds to PI3KC2α in a mode similar to mTOR (Fig. 3b and Extended Data Fig. 4c). The tricyclic benzonapththyridine ring of Torin-2 occupies the hydrophobic adenine pocket and a sulfur–pi interaction contributed by the conserved M1125 located in hydrophobic pocket II. Binding is further enabled by local small scale conformational changes of K1138 and D1268 to accommodate the amino-pyrimidine group of Torin-2 and by hydrophobic contacts of the benzotrifluoride group with M1136 and F1112 (Fig. 3b,c). The reduced half-maximum inhibitory concentration (IC_50_) of Torin-2 for mTOR (IC_50_^mTOR^ = 2.5 nM versus IC_50_^PI3KC2α^ = 64 nM) compared to PI3KC2α is largely due to the pi–pi interaction of the tricyclic benzonapththyridine ring with W2239 inside the mTOR hinge region^36^, a residue not conserved in PI3KC2α (Fig. 3b and Extended Data Fig. 4c). PIK-90, which also displays profound off-target activity toward PI3KC2α (IC_50_^PI3KC2α^ = 78 nM), occupies a similar position to Torin-2 in the ATP site. The imidazoquinazoline ring of PIK-90 binds to the adenine pocket via a single hydrogen bond and hydrophobic region II (Fig. 3d and Extended Data Fig. 4d), while the pyridine ring in the terminal hinge of PIK-90 targets the innermost region of the affinity pocket (Fig. 3d,e). In contrast to Torin-2, association of PI3KC2α with PIK-90 involves comparably minor conformational movements of K1138 and D1268 (Fig. 3e). We conclude that Torin-2 and PIK-90 bind to PI3KC2α via the conserved ATP-binding site common to PI3K-related kinases and PI3Ks and in a manner similar to complex formation with their target enzymes (Extended Data Fig. 4c,d), providing a structural basis for their high affinity but moderate selectivity.

Based on these data and the size and chemical composition of the catalytic site in PI3KC2α we predict that PI3KC2α should in principle be amenable to selective targeting by high-affinity small molecule inhibitors.

### Conformational control of PI3KC2α activity

**Fig. 4:**
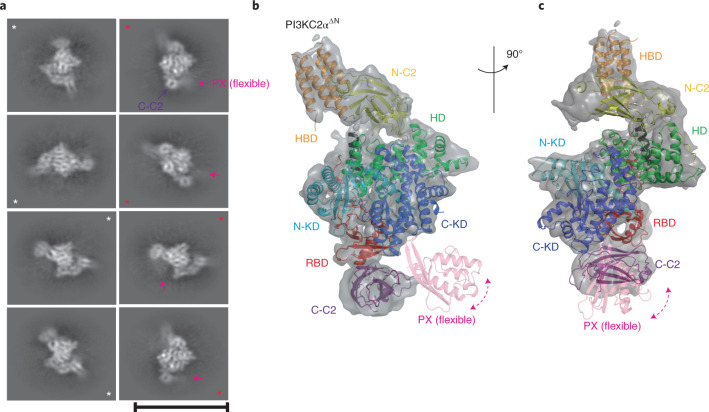
Cryo-EM structure of PI3KC2α^ΔN^. **a**, The selected 2D classes of PI3KC2α^ΔN^ from Extended Data Fig. 5b. The selected 2D classes without or with diffused density of PX domain are labeled with white or red stars. The density of PX domain is shown with a magenta arrow. The donut-shaped C-C2 domain is indicated with a purple arrow. Scale bar, 20 nm. **b**,**c**, 3D cryo-EM map of PI3KC2α^ΔN^ overlaid onto the crystal structures of PI3KC2α^ΔN+ΔC−C2^ and PI3KC2α^core^ and the C-terminal C2 domain of human PI3KC2α (PDB 6BTY). The flexible PX domain is shown as a transparent ribbon, where the EM density was subtracted after 3D reconstruction. The overall structure of PI3KC2α^ΔN^ reveals a closed conformation of the enzyme.The views in **b** and **c** are related by 90° rotation.

**Extended Data Fig. 5:**
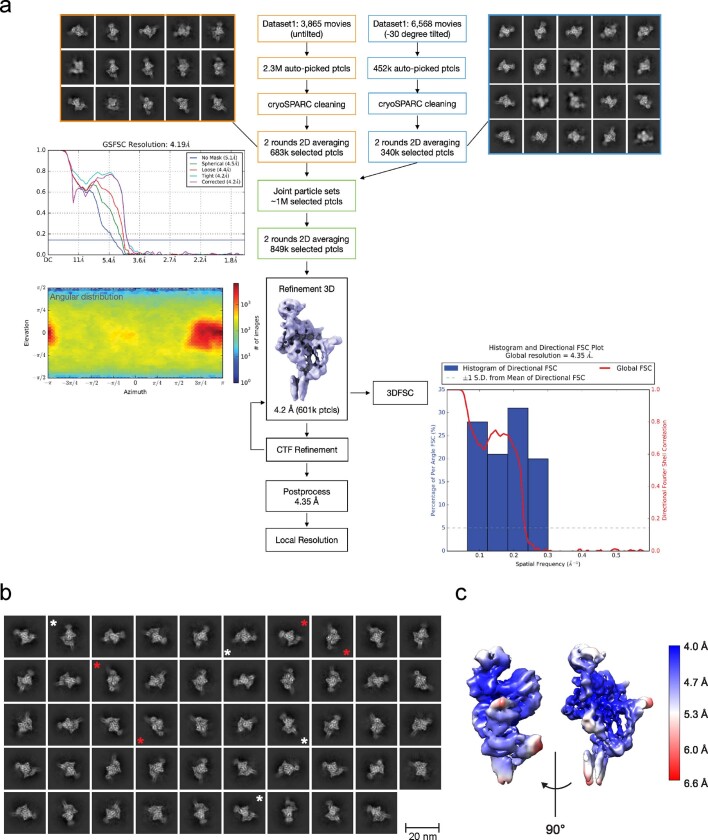
Cryo-EM structure determination of PI3KC2α^ΔN^. **(a)** Cryo-EM image processing workflow. 2.3 ×10^6^ of particles were extracted for 2D classification. 683k particles were selected after two rounds of 2D classification. 340k selected particles from 2D classes based on datasets with 30 tilted angles were joined for a third round of 2D averaging. 601k selected particles were finally used to obtain a 3D reconstruction with a global resolution 4.35 Å. The angular distribution is represented as a heat map to indicate the distributed projections of all particles contributing to the consensus map. **(b)** Representative 2D classes of PI3KC2α^ΔN^ after 2 rounds of 2D averaging. **(c)** Local resolution of the PI3KC2α^ΔN^ EM-map shown as a heat map from 4.04 Å (blue) to 6.71 Å (red) with two different views. The central core region of PI3KC2α displays a compact helix-rich fold with the highest local resolution.

Unlike class I and class III PI3Ks that are activated by membrane binding of their associated subunits^2,4,7,27,34^, class II PI3Ks such as PI3KC2α are autoregulated by their lipid-binding distal PX and C2 domains^30^. To structurally dissect the mechanism of PI3KC2α autoregulation, we purified near full-length PI3KC2α only lacking the intrinsically disordered N-terminal region but containing the distal PX and C2 domains (PI3KC2α^ΔN^) (Extended Data Fig. 1a,b). We then determined the three-dimensional (3D) structure of PI3KC2α^ΔN^ by single-particle cryo-EM (Fig. 4 and Extended Data Fig. 5). Tilted data collection followed by two-dimensional (2D) classification resulted in a reconstruction with a nominal resolution of 4.4 Å from roughly 600,000 particles (Table 2) with substantial resolution anisotropy and the highest resolution in the core domain (Extended Data Fig. 5). The cryo-EM structure of PI3KC2α^ΔN^ unequivocally showed the catalytic core of PI3KC2α, which was overlaid almost perfectly with the PI3KC2α crystal structure. Two additional EM densities were located underneath the core region: a donut-shaped density in 2D classes that adopted a barrel shape in the 3D map. This density could accommodate the previously solved crystal structure of the C-terminal C2 domain of PI3KC2α (ref. ^37^) (Fig. 4). A second, less well-defined density was located in the vicinity of the C-terminal lobe of the catalytic kinase core domain, which likely corresponds to the lipid-binding PX domain (Fig. 4).

**Table 2:** Cryo-EM data collection statistics

	PI3KC2α (untilted) (EMDB-12191)	PI3KC2α (−30° tilted) (EMDB-12191)
**Data collection and processing**		
Magnification	105,000	105,000
Voltage (kV)	300	300
Electron exposure (e^–^/Å^2^)	60	60
Defocus range (μm)	−1.5 to −2.8	−1.5 to −2.8
Pixel size (Å)	0.837	0.837
Symmetry imposed	C1	C1
Initial particle images (no.)	2.3 million	452,000
Final particle images (no.)	601,000 (untilted + tilted)	
Map resolution (Å)	4.4	
FSC threshold	0.143	
Map resolution range (Å)	5.2–4.4	

**Fig. 5:**
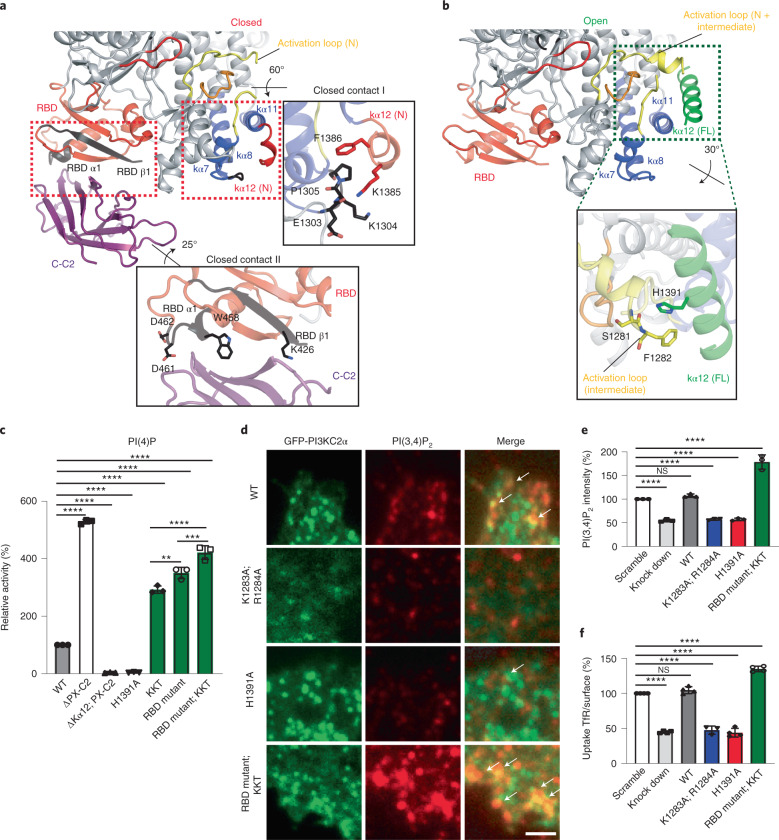
Conformational control of PI3KC2α activity in vitro and at endocytic clathrin-coated pits in living cells. **a**, Closed conformation of PI3KC2α based on the docked model in Fig. 4. Only the N terminus of kα12 (red) is defined. Regions identified as inhibitory contacts by HDX–MS (linker of kα7 and kα8, β1 of the RBD, tip of RBD α1) are shown with black ribbons. Close-up view of closed contacts I and II with key residues indicated. **b**, Open conformation of PI3KC2α based on the crystal structure of PI3KC2α^ΔN+ΔC−C2^. kα12 (green) is completely folded and stabilizes a short helical segment of the activation loop (yellow) that is only defined in the open state. Close-up view indicating crucial interactions between kα12 and the activation loop. **c**, In vitro PI(3,4)P_2_-synthesizing activity of PI3KC2α carrying mutations that disrupt key interactions stabilizing the open or closed states. Deletion of kα12 or the H1391A mutation disrupt the open state and cause inactivation of PI3KC2α. The KKT mutation (^1303^EKP^1305^ to ^1303^KKT^1305^; that is, closed contact I) or mutations in the RBD (K426A, W458A, D462A, D463A; that is, in closed contact II) disrupt the closed state and cause PI3KC2α hyperactivation. A deletion mutant lacking the C-terminal PX-C2 domains serves as a control. Data represent mean ± s.d. from *n* = 3 experiments. One-way ANOVA with Tukey’s multiple comparisons, ^∗∗^*P* = 0.0012; ^∗∗∗^*P* = 0.0002; ^∗∗∗∗^*P* < 0.0001. **d**,**e**, Conformational control of local PI(3,4)P_2-_synthesis mediated by PI3KC2α at endocytic clathrin-coated pits in living cells. **d**, High-magnification close-up views of PI3KC2α-depleted Cos7 cells re-expressing siRNA-resistant variants of eGFP-PI3KC2α WT or mutants (green) stained for PI(3,4)P_2_ (red) and analyzed by total internal reflection fluorescence microscopy. Examples of colocalization of PI3KC2α with PI(3,4)P_2_ are highlighted by white arrows. Scale bar, 50 µm. **e**, Quantified PI(3,4)P_2_ levels at endocytic clathrin-coated pits in PI3KC2α-depleted Cos7 cells re-expressing eGFP-PI3KC2α WT or mutant versions. Data represent s.e.m. from *n* = 3 independent experiments. One-way ANOVA with Tukey’s multiple comparisons, NS, not significant; ^∗∗∗∗^*P* < 0.0001. **f**, Ratio of internalized (10 min, 37 °C) to surface (45 min, 4 °C) transferrin (TfR) in PI3KC2α-depleted Cos7 cells re-expressing eGFP-PI3KC2α WT or mutant versions. Data represent s.e.m. from *n* = 4 independent experiments. One-way ANOVA with multiple Tukey’s comparisons, NS, significant; ^∗∗∗∗^*P* < 0.0001. Source data

**Extended Data Fig. 6:**
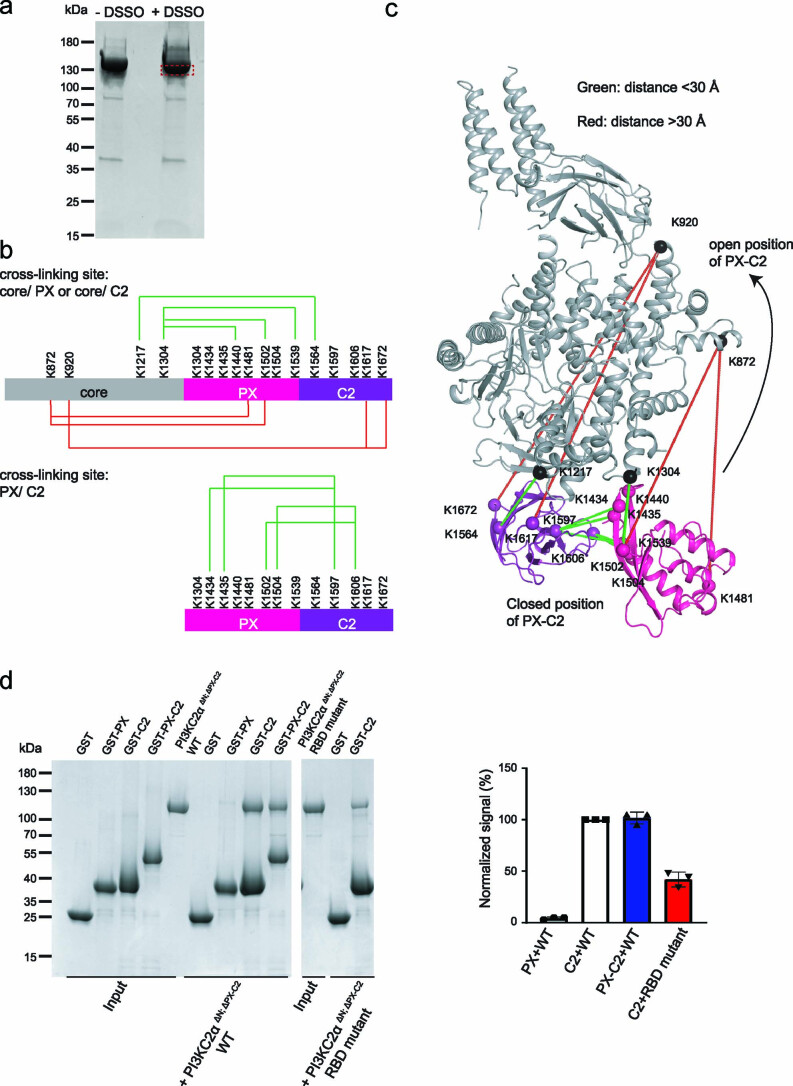
Different conformational states of PI3KC2α. **(a)** PI3KC2α^ΔN^ was crosslinked with DSSO chemical crosslinker and separated by 4–15% gradient SDS-PAGE. The monomeric band of crosslinked PI3KC2α^ΔN^ indicated as dashed red square was cut out for further MS analysis. **(b)** Identified crosslinked peptides using DSSO as chemical crosslinker. **(c)** Model of PI3KC2α^ΔN^ based on the cryo-EM map without the PX domain. The PX domain was docked onto the distal C2 domain using restraints imposed by the obtained crosslinks between the PX and distal C2 domain, using the HADDOCK server. The obtained model was subsequently verified manually using all identified crosslinking sites between the kinase core, PX, and distal C2 domains and the C- to N- terminal connections between these domains. Green line: distance < 30 Å; red line; distance > 30 Å. Distant crosslinks (>30 Å) suggest different conformational states of PI3KC2α. **(d)** Interaction between the kinase core and lipid binding C2 domains of PI3KC2α. GST pull down assays using GST-PX, GST-C2 or GST-PX-C2 as baits to capture PI3KC2α^ΔN+ΔPX−C2^ Wild-type (WT) or RBD mutant (K426A; W458A; D461A; D462A) versions. Samples were analyzed by SDS-PAGE and Coomassie Blue staining and quantified using ImageJ software. Binding of PI3KC2α^ΔN+ΔPX−C2^ WT was set to 100%. Data represent SEM from triplicate (n = 3) experiments. Source data

As the PX domain exhibited significant flexibility within PI3KC2α, we complemented our results from single-particle cryo-EM by crosslinking–mass spectrometry (XL–MS). This analysis (Supplementary Table 2) imposed distance constraints (30 Å, measured by the Cα–Cα distance between two crosslinked residues) of the PX domain relative to the C-C2 domain and the C-terminal lobe of the kinase core domain (Extended Data Fig. 6a–c), and supported docking of the PX domain into the cryo-EM structure^38^. We then used the information derived from cryo-EM, XL–MS and the crystal structure of PI3KC2α^core^ to generate a composite 3D model of PI3KC2α. Ιn this model, only the N-terminal half of kα12 (kα12(N)) is folded whereas the remainder of it is disordered. kα12(N) interacts with the loop between kα7 and kα8 of the KD forming closed contact I. The disordered C-terminal half of kα12 provides the flexibility necessary to place the C-C2 domain in the vicinity of the RBD, where it forms another set of interactions referred to as closed contact II (Fig. 5a). These contacts precisely map to putative autoinhibitory interfaces defined earlier by HDX–MS analysis^30^. In particular, we had reported that mutation of ^1303^EKP^1305^ to ^1303^KKT^1305^ (that is, ‘KKT mutant’), in the kinase core domain in closed contact I, results in elevated lipid kinase activity. Moreover, HDX–MS analysis had identified a putative intramolecular interaction between the RBD and the distal PX-C2 domain module^30^. Our integrative structural analysis identifies this interaction as closed contact II, formed by K426, W458, D461 and D462 on the RBD. Based on these data, we hypothesized that the composite structure of PI3KC2α^ΔN^ represents an inactive conformation that is stabilized by closed contacts I and II.

We further experimentally tested this model. Affinity chromatography experiments showed that the immobilized glutathione *S*-transferase- (GST-)tagged C2, but not the PX domain, directly associates with PI3KC2α^ΔN+ΔPX−C2^ but less well with a mutant version of PI3KC2α, in which the RBD binding interface with the distal C2 domain had been perturbed by mutations in the closed contact I interface (K426A, W458A, D461A, D462A; that is, the ‘RBD mutant’) (Extended Data Fig. 6d). Disruption of either closed contact I in the KKT mutant (^1303^EKP^1305^ to ^1303^KKT^1305^) or of contact II in the RBD mutant (K426A, W458A, D461A, D462A) significantly increased the PI(3,4)P_2_-synthesizing activity of PI3KC2α, a phenotype further augmented in a double KKT/RBD mutant of PI3KC2α, in which both inhibitory interfaces were disrupted (Fig. 5c).

As PI3KC2α transitions from the inactive closed to the active open conformation, the C2 and PX domains likely are dislodged from their positions at the RBD and kinase core, respectively, while kα12 undergoes repositioning and folding into a complete helix (Fig. 5a,b). Consistently, we observed the PX domain to be displaced from the kinase core, and closed contact I involving the N terminus of kα12 to be disrupted in the crystal structure of PI3KC2α^ΔN+ΔC−C2^ (Fig. 1b and Extended Data Fig. 3a). Instead, in this open conformation of PI3KC2α, the kα12 helix interacts via hydrogen bonding of H1391 with the backbone of a short helical segment within the intermediate section of the activation loop (Fig. 5b). In the open conformation, the C-terminal part of the activation loop known to be crucial for lipid binding remains flexible to enable catalysis.

**Fig. 6:**
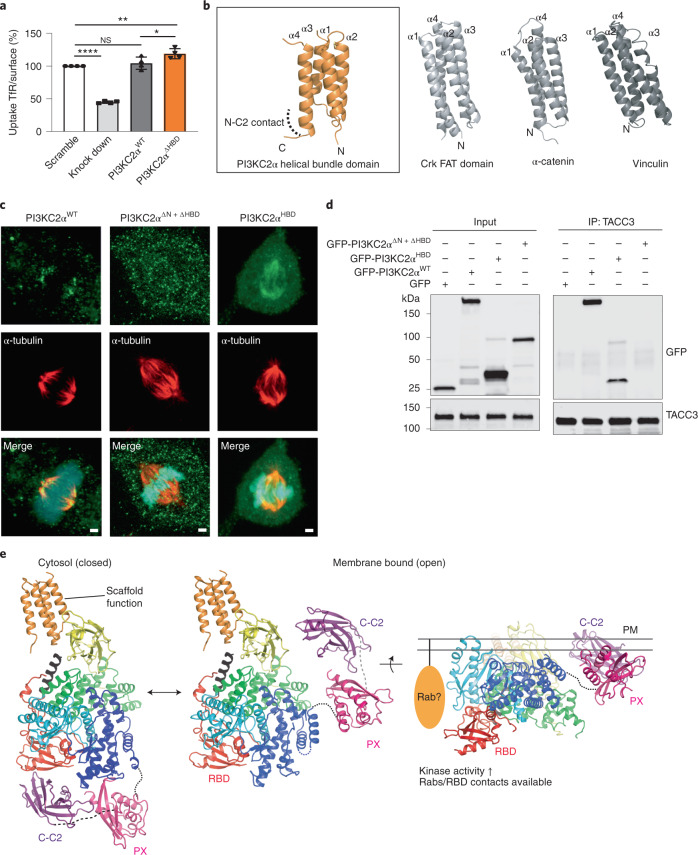
Structural basis of the scaffolding function of PI3KC2α and model for PI3KC2α activation at membranes. **a**–**c**, The unique HBD underlies the scaffold function of PI3KC2α. **a**, Bar diagrams representing the ratio of internalized (10 min, 37 °C) to surface (45 min, 4 °C) transferrin in PI3KC2α^WT^ or PI3KC2α^ΔHBD^ expressing Cos7 cells depleted of the endogenous PI3KC2α enzyme. The HBD domain is dispensable for clathrin-mediated endocytosis. Data represent s.e.m. from *n* = 4 independent experiments, one-way ANOVA with Tukey’s multiple comparisons, NS, not significant; ^∗^*P* = 0.0262; ^∗∗^*P* = 0.0046; ^∗∗∗∗^*P* < 0.0001. **b**, Structure of PI3KC2α HBD and its closest structural relatives, that is, focal-adhesion targeting (FAT) domain of Crk-associated substrate (Cas) and the F-actin binding domains of vinculin and α-catenin. Homology search and ranking was conducted using the DALI server (PDB 3T6G, *Z* = 9.6, r.m.s.d. = 2.2 Å; PDB 6NR7, *Z* = 8.5, r.m.s.d. = 2.0 Å, PDB 4IGG, *Z* = 8.5, r.m.s.d. = 2.1 Å). The identified homologs share functional features by serving as scaffolds for protein–protein interactions. **c**, The HBD targets PI3KC2α to the mitotic spindle. Confocal images of fixed metaphase-arrested HeLa cells expressing eGFP-PI3KC2α^WT^, PI3KC2α^ΔN+ΔHBD^ or PI3KC2α^HBD^ immunolabelled for α-tubulin (red) (representative of three independent experiments). eGFP-PI3KC2α^WT^ or PI3KC2α^HBD^ localize to the mitotic spindle, whereas a mutant lacking the HBD (PI3KC2α^ΔN+ΔHBD^) displays a diffuse cytosolic localization. DAPI, cyan; PI3KC2α, green and α-tubulin, red. Scale bars, 5 µm. **d**, TACC3 binds to the HBD of PI3KC2α. Coimmunoprecipitation of endogenous TACC3 and eGFP-PI3KC2α HBD in metaphase-arrested HEK293T cells expressing WT or mutant versions of eGFP-PI3KC2α, or eGFP. Endogenous TACC3 was immunoprecipitated using anti-TACC3 antibodies (IP, immunoprecipitation) and bound proteins were detected using anti-GFP antibodies. WCL, whole cell lysate. Representative data from three independent experiments are shown. **e**, Model for PI3KC2α activation at endocytic membranes. A PI(4,5)P_2_-induced large-scale conformational rearrangement of the enzyme causes the displacement of the distal C2 domain from the RBD and is likely facilitated by complex formation of the RBD with a Rab protein. The concomitant association of the disordered N-terminal region of PI3KC2α with clathrin has been omitted for clarity. Source data

**Extended Data Fig. 7:**
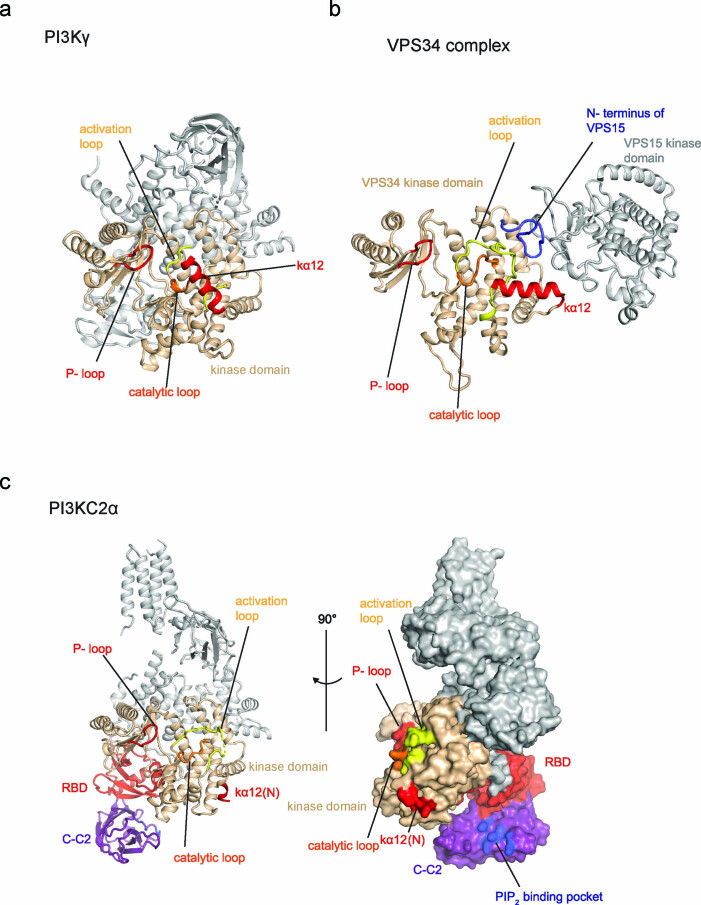
Comparison of the inhibitory conformations of PI3Ks. **(a)** Auto-inhibited conformation of PI3Kγ (pdb: IE8X). kα12 (red) bends inward towards the catalytic pocket. **(b)** Auto-inhibited conformation of yeast Vps34/Vps15 in complex II (pdb: 5DFZ). The activation loop of VPS34 (yellow) is locked by kα12 (red) and the N-terminus of VPS15 (blue). **(c)** Auto-inhibited conformation of PI3KC2α^ΔN^ as ribbon and surface representation. The distal C2 domain binds to the RBD. The yellow and orange regions represent the catalytic pocket and the activation loop, where lipid substrates bind. The PI(4,5)P_2_-binding surface in the distal C2 domain is colored in blue. The locations of the lipid-binding pockets are distributed on different sides of the PI3KC2α molecule in the closed conformation.

This mechanism is distinct from the function of kα12 in other PI3Ks. PI3Kγ uses kα12 to capture the catalytic loop in an inactive state (Extended Data Fig. 7a), whereas in VPS34, kα12 interacts with the N terminus of VPS15 to trap the activation loop^7^ (Extended Data Fig. 7b). Finally, conformational opening requires the lipid-binding distal C2 domain to be displaced from its inhibitory contact with the RBD. In the closed conformation, association with the RBD renders the lipid-binding surface of the C2 domain difficult to access and misaligns it with the substrate lipid-binding site in the activation loop (Extended Data Fig. 7c). Hence, we predict the distal C2 domain to be flexibly positioned away from the kinase core in the open conformation of the enzyme (Fig. 6e and below). A further prediction from this structure-based activation mechanism is that disruption of the interaction between kα12 and the activation loop in the open form should abrogate lipid kinase activity. In vitro kinase assays confirmed that the H1391A mutation in the center of the kα12-activation loop interface resulted in a complete loss of enzymatic activity (Fig. 5c). H1391 is thus required to stabilize the open catalytically active conformation of PI3KC2α.

Our combined data indicate a molecular model for the conformational control of PI3KC2α activity by large-scale rearrangements in the position of the lipid-binding PX and C2 domains that is accompanied by refolding and repositioning of the kα12 helix critical for catalysis.

### Conformational control of PI3KC2α function in cells

We tested this structure-based model for the conformational activation of PI3KC2α at membranes by analyzing the PI(3,4)P_2_-synthesizing activity of PI3KC2α during endocytic membrane dynamics. Depletion of PI3KC2α from Cos7 cells resulted in reduced levels of PI(3,4)P_2_ at endocytic plasma membrane coated pits and a concomitant reduction in clathrin-mediated endocytosis of transferrin. These defects were rescued by re-expression of the small interfering RNA-resistant wild-type (WT) enzyme (Fig. 5d–f) or a mutant lacking the HBD (Fig. 6a), in agreement with its presumed scaffolding role during mitosis. In contrast, PI3KC2α mutant versions defective in PI(4)P substrate binding (K1283A,R1284A) or lacking critical hydrogen bonding via H1391 to stabilize the open conformation (H1391A) failed to restore PI(3,4)P_2_ levels and defective endocytosis. Conversely, conformational activation of PI3KC2α by disrupting closed contacts I and II via the combined KKT and RBD mutations led to elevated cellular PI(3,4)P_2_ synthesis and a gain in endocytic transferrin uptake (Fig. 5d–f). These results confirm that structural changes in the position of the lipid-binding PX and C2 domains and the kα12 helix underlie the conformational activation of PI3KC2α at membranes in vivo.

A further prediction from our combined structural and biochemical studies is that distinct structural elements mediate the catalytic roles of PI3KC2α in endocytic membrane dynamics and its noncatalytic function at the mitotic spindle^1,14^. The unique HBD of PI3KC2α, which points away from the KD (Figs. 1b and 4c), is dispensable for catalytic activity in vitro (Extended Data Fig. 1c) and for endocytosis in vivo (Fig. 6a). We therefore hypothesized, that the HBD might facilitate targeting of the enzyme to the mitotic spindle by associating with the microtubule-binding protein TACC3 (ref. ^26^). Consistently, we found that the HBD of PI3KC2α with its four antiparallel α-helices displays strong structural homology to cytoskeletal proteins, such as the focal-adhesion targeting domain of Crk-associated substrate (Cas) and to the F-actin binding domains of vinculin and α-catenin (Fig. 6b). To probe the possible function of the HBD in targeting of PI3KC2α to the mitotic spindle, we examined the subcellular localization of an N-terminally truncated PI3KC2α lacking the clathrin binding region, a mutant version thereof, in which the HBD was deleted (PI3KC2α^ΔN+ΔHBD^), or the isolated HBD alone (PI3KC2α^HBD^). The HBD was sufficient for targeting to the mitotic spindle and for association with TACC3 (Fig. 6c,d), whereas deletion of the HBD abrogated the spindle localization of PI3KC2α^ΔN^ (Fig. 6c) and complex formation with TACC3 (Fig. 6d). These data uncover the unique HBD as an important structural element that underlies the scaffolding function of PI3KC2α at the mitotic spindle^26^. We note that while the presence of the HBD is conserved among the members of the class II PI3K subfamily, its relatively low level of sequence conservation (Extended Data Fig. 2) suggests that they interact with different protein binding partners to execute putative noncatalytic functions.

## Discussion

Our integrated structural analysis of PI3KC2α reveals different conformational states of the enzyme that suggest a molecular model for the local activation of PI3KC2α at endocytic membranes (Fig. 6e). In its cytosolic form, the enzyme is present in a closed inactive conformation that is stabilized by intramolecular contacts within the kinase core domain that occlude catalysis and an inhibitory interface between the RBD and the distal C2 domain, which may be further augmented by placement of the PX domain at the interface between the distal C2 and the C-terminal lobe of the kinase core domain, in agreement with our earlier biochemical data^30^. Clathrin-mediated recruitment^15,39^ and activation of PI3KC2α at endocytic membranes involves a large-scale conformational change within the single subunit enzyme that releases PI3KC2α from autoinhibition to enable local PI 3-phosphate synthesis (Fig. 5). In this active conformation, the substrate PI(4)P is bound by basic residues (that is, K1283, R1284) within the PI3KC2α activation loop (Fig. 2d–f). Of note, these residues are absent from Vps34, a PI3K that is unable to use PI(4)P as a substrate, providing a molecular explanation for the distinct catalytic activities of PI3KC2α and related class II PI3Ks (refs. ^1,2,14^).

The mechanism of activation of PI3KC2α is distinct from that of all other PI3Ks, in which membrane binding and catalytic activation are induced by conformational transitions in tightly associated accessory subunits (compare Fig. 1c). In class I PI3K, hydrophobic residues in the C-terminal tail of the KD not present in PI3KC2α as well as basic amino acids in the charged activation loop are only allowed to contact the membrane once the enzyme has been released from allosteric inhibition by its regulatory p85 subunit^40^. The class III Vps34 complex binds to membranes via the tips of two arms that is three of its four subunits: one contact is formed by the catalytic Vps34 subunit and the Vps15 myristoylation site, the other one involves the Vps30/Beclin 1 BARA domain^7^.

The unique mechanism of membrane binding and activation of PI3KC2α is not only interesting from a mechanistic viewpoint, but also bears important implications for our understanding of class II PI3K biology. Our structural data predict that the conformational activation and, thereby, the catalytic function of PI3KC2α is triggered by multiple coincident signals, most notably, the membrane association of its PX and C2 domains^30,37^. The exquisite lipid-binding specificity of these domains for PI(4,5)P_2_ thereby limits PI3KC2α activity to nanoscale sites enriched in PI(4,5)P_2_, providing a structural explanation for the observed spatiotemporal restriction of PI3KC2α-mediated synthesis of PI(3,4)P_2_ or PI(3)P at late-stage endocytic pits^15,33,41^ and the base of primary cilia^13,42^. Our structural and biochemical data further predict that the conformational activation of PI3KC2α, and likely other class II PI3K family members, further requires or is facilitated by complex formation of the RBD with an endocytic Rab protein^12,13^. Rab association would aid displacement of the distal C2 domain from the RBD and, thereby, act as a third coincidence determinant, in addition to clathrin^15,39^ and PI(4,5)P_2_ (ref. ^30^). The structure-based mechanism for the activation of PI3KC2α at membranes described here therefore predicts that the multiple physiological functions of PI3KC2α, for example in endocytic receptor internalization and recycling^12,15,16,33^, VEGF-driven angiogenesis^19^ and viral replication^21,22^, result from and are defined by the coincident interaction of PI3KC2α with PI(4,5)P_2_ and different Rab proteins that steer its catalytic activity to distinct nanoscale sites. Identifying the respective Rab protein underlying these activities will be key to our understanding of the physiological functions of PI3KC2α in cell physiology and disease.

Additionally to providing insights into the mechanism of PI3KC2α activation and function, our structural analysis of PI3KC2α in complex with nonselective PI3K inhibitors will undoubtedly serve as a door-opener for rational development of isoform-selective PI3KC2α inhibitors and other class II PI3K family members that may provide new therapeutic avenues for the treatment of important human diseases such as thrombosis^28^, viral infection^21,22^, diabetes^29^ or cancer^1,14^. Finally, we provide a structural basis for the scaffolding function of PI3KC2α at the mitotic spindle that involves the association of its unique HBD with the microtubule-associated kinetochore protein TACC3 (Fig. 6). These structural insights will be of relevance to develop new therapeutics to fight cancer and cancer metastasis^14,26^.

## Methods

### Oligonucleotides

Oligonucleotide sequences used in this study are listed in Supplementary Table 1.

### Cell lines

HeLa, human embryonic kidney 293T (HEK293T) and Cos7 cells were obtained from ATCC and cultured in DMEM with 4.5 g l^−1^ glucose (Lonza) containing 10% heat-inactivated FBS, 100 U ml^−1^ penicillin and 100 μg ml^−1^ streptomycin (Gibco). Cells were routinely tested for and devoid of mycoplasma contamination.

### Cloning and mutagenesis

Complementary DNA encoding mouse PI3KC2α was synthesized from total RNA extracted from mouse brain using a gene specific primer (5′TAGATACGTTGCCGCAGTCAGCTG3′) (Supplementary Table 1) according to the SuperScript III protocol. For baculovirus-mediated expression in insect cells, cDNA encoding mouse PI3KC2α^ΔN^ (amino acids 377–1686), PI3KC2α^ΔN+ΔC−C2^ (amino acids 377–1,539) and PI3KC2α^ΔN+ΔPX−C2^ (amino acids 377–1,400) was amplified by PCR and cloned into pFL10His via KasI/XbaI restriction sites. Mutations were introduced by site-directed mutagenesis using PCR. Crystallization construct I, PI3KC2α^ΔN+ΔC−C2^ contains a re-engineered internal loop with amino acids 533–544 being replaced by the amino acid sequence GSGS. In PI3KC2α^core^, the HBD (amino acids 550–665) was replaced by the sequence SGAGSGA. GST-PX (amino acids 1409–1539), GST-PX-C2 (amino acids 1,409–1,686) and GST-distal C2 (amino acids 1,561–1,686) were cloned into pGEX-4T-1 using BamHI and NotI sites for expression in *Escherichia coli*. The siRNA-resistant enhanced green fluorescent protein- (eGFP-)PI3KC2α mutant was generated by site-directed mutagenesis PCR based on siRNA-resistant eGFP-PI3KC2α used in our earlier studies^15,30^.

### Protein expression and purification

His_10_-tagged PI3KC2α^ΔN^, PI3KC^ΔN+ΔC−C2^, PI3KC2α^core^ and related mutants were expressed in *Sf*21 insect cells, using SF900-II serum-free media (ThermoFisher). In brief, *Sf*21 cells (800 ml) grown to a density of 1.5–2 × 10^6^ cells per ml were infected with 8 ml amplified baculovirus encoding the desired construct. Cells were collected when the viability was less than 90%. Cell pellets were stored frozen at −20 °C until purification. For purification, cell pellets from each 200 ml of culture were resuspended in 35 ml of lysis buffer (50 mM Tris pH 7.2, 300 mM NaCl, 10 mM imidazole, 1 mM dithiothreitol (DTT), 0.5% Triton X-100, 1 tablet per 50 ml of protease inhibitor cocktail), sonicated for 1 min (1 s pulse on, 5 s pulse off) and centrifuged for 20 min at 87,000*g*. Then 50 ml of supernatant were incubated with 0.5 ml Nickel NTA beads (Sigma Inc.) on a rotating wheel for 1 h. Beads were collected in an open column, washed with 20 ml of lysis buffer, then with 30 ml of wash buffer (50 mM Tris pH 7.5, 300 mM NaCl, 20 mM imidazole, 1 mM DTT). Protein was eluted with 8 ml of elution buffer (20 mM Tris pH 7.5, 300 mM NaCl, 300 mM imidazole, 5 mM DTT). The His_10_-tag was released by tobacco etch virus (10 mg of protein per 0.25 mg of tobacco etch virus) cleavage overnight, while dialyzing against size-exclusion chromatography (SEC) buffer (20 mM Tris pH 7.5, 300 mM NaCl, 5 mM DTT) at 4 °C. Proteins were purified on a Superdex 200 gel filtration column at 4 °C with SEC buffer (20 mM Tris pH 7.5, 300 mM NaCl, 5 mM DTT). Proteins were concentrated to about 2.4 mg ml^−1^ for PI3KC2α^ΔN^, 2.5 mg ml^−1^ for PI3KC2α^ΔN+ΔC−C2^, 5 mg ml^−1^ for PI3KC2α^core^ and 1–2 mg ml^−1^ for other mutants. All proteins were flash frozen in liquid nitrogen and stored at −80 °C.

### Crystallization of PI3KC2α^ΔN+ΔC−C2^ and PI3KC2α^core^

To obtain PI3KC2α^ΔN+ΔC−C2^ crystals, >1,000 conditions were screened using a Crystal Gryphon robot setup (Art Robbins) with 200 nl of protein solution (2.5 mg ml^−1^) and 200 nl of screen solution in 96-well sitting-drop plates. Initial crystals were observed with a 50% water-diluted kit from Molecular Dimensions Morpheus E10 and E11, which originally contains 0.12 M ethylene glycol mix, 0.1 M Tris/Bicine, 40% v/v ethylene glycol, 20% w/v polyethylene glycol (PEG) 8,000 (in E10) or 40% glycerol and 20% PEG 4,000 (in E11). Optimal crystals were obtained at room temperature by microseeding in 24-well sitting-drop plates in mother liquid containing 0.1 M Tris pH 7.5, 8–9% PEG 20,000, 10% ethylene glycol and 10% formamide (for the protein complex with Torin-2:mother liquid:seed of 1:5:0.5 μl). Thin plate-shaped crystals grew into large clusters within 2–3 d. Single crystals were isolated and washed in cryogenic solution (50% fresh prepared mother liquid, 50% buffer (20 mM Tris pH 7.5, 150 mM CsI, 5 mM DTT), supplied with 10% ethylene glycol). Initial PI3KC2α^core^ crystals were obtained using homemade screening buffers containing 0.1 M Tris pH 7.5, 200 mM MgSO_4_, 10% PEG 8,000. Concentrated protein sample was filtered with 0.2-μm spin filters and final crystals were grown in 0.1 M Tris pH 7.5, 100–200 mM MgSO_4_, 7–10% PEG 3,350. Crystals were cryo-protected with mother liquid supplied with 10% ethylene glycol. For ligand soaking, 2 mM ATP, 1 mM Torin-2, 0.5 mM PIK-90 were prepared in cryoprotection solution and incubated with protein crystals for 30 min. Crystals were mounted in a nylon loop and flash cooled in liquid nitrogen.

### Data collection, model building and refinement

Diffraction data were collected at station BL14.1 of BESSY/Helmholtz Center Berlin (HZB). Images were processed with XDSAPP^43^. The PI3KC2α^ΔN+ΔC−C2^ structure was determined by molecular replacement with the PHENIX suite^44^ using the helical domains and KDs of PI3Kγ (PDB 1E8X) and the PX domain of PI3KC2α (*2IWL*) as search models. The structure was manually built using COOT and iteratively refined using Refmac^45^ and BUSTER^46^ PI3KC2α^core^ structures were determined by molecular replacement with PHENIX using the crystal structure of PI3KC2α^ΔN+ΔC−C2^ as a search model. The structures were manually built using COOT^47^ and refined with PHENIX and BUSTER. Data collection and structure refinement statistics are summarized in Table 1. Ramachandran statistics in the order of favored, allowed, outliers for each structure are: PI3KC2α^ΔN+C−C2^ (95.26, 4.74, 0%), PI3KC2α^core^ apo (96.91, 2.97, 0.12%), PI3KC2α^core^ in complex with ATP-Mg^2+^ (94.6, 5.28, 0.12%), PI3KC2α^core^ in complex with Torin-2 (98.76, 1.11, 0.12%) and PI3KC2α^core^ in complex with PIK-90 (96.78, 3.22, 0%). Structural data were deposited in the Protein Data Bank (PDB) and are available under accession numbers 7BI2, 7BI4, 7BI6 and 7BI9.

### Negative stain screening of PI3KC2α^ΔN^ in buffer with different salt concentrations

Purified PI3KC2α^ΔN^ (roughly 2.4 mg ml^−1^, in 20 mM Tris-HCl buffer, with 300 mM NaCl at pH 7.4) was diluted to a final concentration of 0.02 mg ml^−1^ into buffers containing 20 mM Tris-HCl, and various concentrations of NaCl (50 to 300 mM) at pH 7.4, before being negatively stained with 2% (w/v) uranyl formate. Negatively stained images were collected using a Tecnai Spirit BioTwin transmission electron microscope (Thermo Fisher Scientific) at 120 kV at a nominal magnification of ×49,000 (2.26 Å per pixel) on a Gatan Rio CCD camera (4,000 × 4,000). The sample diluted in a buffer containing 100 mM NaCl presented a good particle distribution without showing significant aggregates. A group of images was collected at a defocus around −1.5 to −3.5 μm. Contrast transfer function (CTF) estimation was performed with CTFFIND 4.1 (ref. ^48^). Particles were auto-picked using Gautomatch-0.53 (https://www2.mrc-lmb.cam.ac.uk/research/locally-developed-software/zhang-software/), and 2D class averages were performed with RELION-2.0 and higher^49^.

### Cryo-EM sample preparation of PI3KC2α^ΔN^

Purified PI3KC2α^ΔN^ (0.8 mg ml^−1^ in 20 mM Tris-HCl, 100 mM NaCl at pH 7.4) were used for plunge-freezing. Double-application of 3 μl of diluted PI3KC2α^ΔN^ were applied onto freshly plasma-cleaned (NanoClean, model 1070, Fischione Instruments) QUANTIFOIL Holey Au-carbon-R2/2 specimen grids, and vitrified by plunge-freezing into liquid ethane using a Mark IV Vitrobot device (Thermo Fisher Scientific). Cryo-EM specimen prepared with different blotting conditions (blot force, blot time) were screened. Final datasets were collected from the cryo-EM grids with thinner uniform ice thickness and good particle orientation distribution.

### Cryo-EM single-particle data collection of PI3KC2α^ΔN^ specimen

The cryo-EM single-particle datasets of PI3KC2α^ΔN^ were collected without and with −30° stage-tilting on a Titan Krios cryo-transmission electron microscope (Thermo Fisher Scientific) operated at 300 kV and equipped with a K3 direct electron detector (Gatan, Inc.) device at a nominal magnification of ×105,000 yielding a pixel size at the specimen of 0.837 Å per pixel in counting mode (Table 2). Videos were collected with EPU, a data collecting automation software package (Thermo Fisher Scientific), with an imaging setting of total exposure equal to 60 electrons over 50 fractions in 3 s and a defocus range between −1.5 and −2.8 μm, at the Max Planck Institute of Biophysics, Frankfurt, Germany.

### Cryo-EM image processing

During data collection, all datasets were preprocessed ‘on-the-fly’ using cryoSPARC live^50^ running video motion correction, CTF estimation and automatic particle picking and stream 2D classification to estimate particle quality. As the streamed 2D classification showed particle features as expected, more data automated positions were defined. Once data collection was completed, all videos from both datasets were imported into cryoSPARC, processed with patch motion correction and CTF estimation, and auto-picked separately. The auto-picked particles were inspected and extracted with a box size of 260 pixels to perform particle cleaning using several rounds of 2D classification and 3D heterogeneous refinement (3D classification, if effective) with C1 symmetry, accordingly. The most homogenous particle sets after cleaning and separation from both datasets were merged, containing about 1 million particles, and taken to perform 3D homogeneous refinement and further 2D classifications as necessary. 3D variability was performed to show the heterogeneity of the PI3KC2α^ΔN^ specimen due to its flexibility. Fourier shell correlation (FSC) estimation was done both in cryosparc and Relion. Postprocessing and local resolution was estimated in RELION-3.0, and global directional resolution estimation was performed using 3DFSC (ref. ^51^). A detailed image processing pipeline is shown in Extended Data Fig. 5. Cryo-EM data were deposited in the PDB and are available under accession number EMD-12191. Original EM micrographs were deposited in the Electron Microscopy Public Image Archive (EMPIAR) (code EMPIAR-10665).

### ADP-Glo kinase assay of PI3KC2α

Purified PI3KC2α variants were prediluted to 0.5 mg ml^−1^ in SEC buffer used for purification. All variants were subsequently diluted to 20 μg ml^−1^ in kinase buffer (5 mM HEPES/KOH, pH 7.2, 25 mM KCl, 2.5 mM Mg (OAc)_2_, 150 mM K-glutamate, 10 μM CaCl_2_, 0.2% CHAPS). Native liver PI or PI 4-phosphate (PI(4)P) were dissolved to a concentration of 400 μM with kinase buffer by water-bath sonification and then supplied with 200 μM ATP. The buffer supplied with 200 μM ATP served as a negative control. Reactions were started by mixing 5 μl of protein stock with 5 μl of substrate solution and incubated for 20 m at room temperature. The reactions were stopped by adding 10 μl of ADP-Glo reagent (Promega). After 40 min of incubation, 20 μl of Kinase Detection Reagent were added. After 20 min incubation, luminescence was read with a TECAN plate reader. IC_50_ measurements of Torin-2 and PIK-90 were carried out using concentration series of these compounds.

### XL–MS analysis of PI3KC2α^ΔN^

Purified PI3KC2α^ΔN^ was buffer exchanged to crosslinking buffer (20 mM BisTris-propane, pH 7.5, 300 mM NaCl) by dialysis. 1.2 mg ml^−1^ PI3KCα^ΔN^ was diluted into 20 mM BisTris-propane, pH 7.5, to final concentration of 0.4 mg ml^−1^. Crosslinking was performed by adding 0.5 μl of disuccinimidyl sulfoxide (DSSO) (stock concentration 50 mM) to 50 μl diluted PI3KC2α^ΔN^. The reaction was performed twice for 20 min each. Samples were quenched by addition of 1 M Tris pH 7.5 (final concentration 20 mM) for 30 min. Crosslinked PI3KC2α^ΔN^ was separated by SDS–PAGE followed by in-gel tryptic digest. Crosslinked peptides were analyzed using a Thermo Scientific Dionex UltiMate 3000 system combined to an Orbitrap Fusion Lumos mass spectrometer. Cross-link acquisition was performed using a MS2-MS3 method. MS2 spectra were acquired on every selected MS1 precursor whereas MS3 acquisitions were triggered if a unique mass difference of 31.9721 was observed in the MS2 spectrum^52^. Data analysis was conducted using XlinkX standalone^52^ with the following parameters: minimum peptide length of 6; maximal peptide length of 35; missed cleavages of 3; fix modification: Cys carbamidomethyl of 57.021 Da; variable modification, Met oxidation of 15.995 Da; DSSO crosslinker 158.0038 Da (short arm 54.0106 Da and long arm 85.9824 Da); precursor mass tolerance of 10 ppm and fragment mass tolerance of 20 ppm. Results were reported at 1% false discovery rate (FDR) at the level of cross-link spectrum matches (CSM).

### Model of PI3KC2α^ΔN^

To build the model with PX and C2 domain, all of identified crosslinked sites between PX and C2 were introduced with 5–15 Å (Cβ to Cβ) distance restraints for docking of PX and C2 by HADDOCK. In total, 24 docked PX-C2 structures from six different clusters were generated. The PX-C2 domain together with PI3KC2α^core^ were used to fit into the EM map with Chimera. The best fitted structures were validated with the crosslinked sites between PI3KC2α^core^ and PX-C2 domain to obtain the final model.

### GST pull down assay

GST pull down assays were performed with immobilized GST-fused PX, C2 or PX-C2 of PI3KC2α as baits. Then 10 μg of immobilized GST fusion protein was used to capture 200 μg of PI3KC2α^ΔN+ΔPX−C2^ WT or PI3KC2α^ΔN+ΔPX−C2^ RBD mutant in 500 μl of binding buffer (20 mM Tris, pH 7.5, 100 mM NaCl). Samples were incubated on a rotating wheel for 1 h at 4 °C, unbound material was removed by washing three times and bound proteins were eluted with 2× SDS–PAGE sample buffer. Samples were analyzed by SDS–PAGE and Coomassie blue staining.

### Plasmid transfections

Cells were seeded and transfected with 50 μM siRNA on day 1 using jetPRIME (Polyplus Transfection) according to the manufacturer’s instructions by reverse transfection. A second round of knockdowns was performed on day 2. Cells were plated onto a Matrigel (BD Biosciences) -coated cover slides in the morning of day 3 and transfected with eGFP-PI3KC2α constructs using jetPRIME (Polyplus Transfection) according to the manufacturer’s instructions.

### Analysis of the localization of eGFP-PI3KC2α in metaphase-arrested cells

HeLa cells were synchronized in metaphase using 2 mM thymidine (Sigma-Aldrich) for 20 h, 30 μM deoxycitidine for 6 h (Sigma-Aldrich) and 50 ng ml^−1^ nocodazole (Sigma-Aldrich) for 12 h, followed by 2 h release in fresh medium in the presence of 20 mM MG132 (Calbiochem). HeLa cells were blocked in interphase by starvation for 16 h. Synchronized cells were transfected with plasmids encoding eGFP-PI3KC2α WT or mutants. For immunofluorescence, the cells were fixed by ice-cold methanol. HeLa cells were permeabilized with 0.1% Saponin for 10 s and then fixed in 2% paraformaldehyde (PFA) for 5 min. Staining for GFP, α-tubulin and 4,6-diamidino-2-phenylindole (DAPI) was performed, and the cells were examined with a Zeiss Observer-Z1 microscope, equipped with the Apotome, Leica TCS-II SP5 or a Leica TSC-II SP8 confocal microscope.

### Coimmunoprecipitation of TACC3 and PI3KC2*α*

HEK293T were transfected with plasmids encoding eGFP-PI3KC2α WT or mutants, mitotically arrested 24 h post-transfection and gathered as reported in ref. ^26^. The following primary antibodies were used: TACC3 (Rabbit, no. 8069, Cell Signaling) and homemade GFP (Rabbit, polyclonal). Membranes probed with the indicated antibodies were then incubated with HRP-conjugated anti-Rabbit IgG light chain (1:5,000, 211-032-171, Jackson ImmunoResearch) and developed with enhanced chemiluminescence (ECL, BD).

### PI(3,4)P_2_ detection at the plasma membrane

PI3KC2α KD Cos7 cells or PI3KC2α KD Cos7 cells re-expressing eGFP-PI3KC2α WT or mutants were grown on Matrigel (BD Biosciences)-coated eight-well glass bottom μ-slide (ibidi). Cos7 cells were washed with PBS containing 10 mM MgCl_2_ once and fixed in 2% PFA with 0.5% glutaraldehyde for 20 min at room temperature. Cells were washed three times with PBS and twice with PBS containing 50 mM NH_4_Cl. Cells were permeabilized with PBS containing 0.5% Saponin and 1% BSA for 30 min. PI(3,4)P2 antibody (Echlon catalog no. Z-P034b) and Goat anti-Mouse IgG (H + L) AF647 labeled secondary antibody (Thermo Fisher catalog no. A21237) were incubated for 2 and 1 h, respectively, in PBS buffer containing 1% BSA and 10% normal goat serum. Cells were analyzed by total internal reflection fluorescence microscopy (Nikon TI Eclipse, 488 and 561 nm laser, ×60 numerical aperture 1.49 objective and sCMOS Andor mNeo). Plasma membrane PI(3,4)P_2_ levels at clathrin-coated pits were quantified using ImageJ software.

### Transferrin uptake and surface labeling

Cos7 cells transfected with siRNA and/or PI3KC2α (WT or mutant)-encoding plasmids or pretreated for 4 h with 0.1% dimethylsulfoxide or 20 μM PITCOIN1 were starved in serum-free DMEM media for 1 h. For transferrin uptake, cells were incubated with 25 mg ml^−1^ Alexa647 labeled transferrin (Molecular Probes, Invitrogen) for 10 min at 37 °C in a humidity chamber. Cells were washed twice with ice-cold PBS supplied 10 mM MgCl_2_ and then acid washed twice at pH 5.3 (0.2 M sodium acetate, 200 mM sodium chloride) on ice for 2 min to remove surface-bound transferrin. Cells were then washed twice more with ice-cold PBS containing 10 mM MgCl_2_ and fixed with 4% PFA for 45 min at room temperature. For surface labeling, cells were incubated with 25 mg ml^−1^ Alexa647 labeled transferrin at 4 °C for 45 min and then washed three times with ice-cold PBS (10 mM MgCl_2_) on ice for 1 min. Cells were fixed with 4% PFA for 45 min at room temperature. Transferrin labeling was analyzed using the Nikon Eclipse Ti microscope and ImageJ software. Internalized transferrin per cell was quantified and normalized to the amount of surface-bound transferrin determined in the same experiment as a measure for the efficiency of internalization.

### Statistical analysis

All data are presented as mean ± s.e.m. and were obtained from ≥3 independent experiments with total sample numbers provided in the figure legends. Statistical significance was evaluated by Prism software (GraphPad), using one simple, two-tailed *t*-test with theoretical mean of 100 or one-way analysis of variance (ANOVA) test with Tukey’s multiple comparisons. Specific *P* values are indicated in the legends to figures. Significant differences were marked as *P < 0.05, **P < 0.01, ***P < 0.001 and ****P < 0.0001.

### Reporting Summary

Further information on research design is available in the Nature Research Reporting Summary linked to this article.

## Online content

Any methods, additional references, Nature Research reporting summaries, source data, extended data, supplementary information, acknowledgements, peer review information; details of author contributions and competing interests; and statements of data and code availability are available at 10.1038/s41594-022-00730-w.

## Supplementary information

Supplementary Information Supplementary Table 1 and PDB validation report.

Reporting Summary

Peer Review Information

Supplementary Table 1 Crosslinking results.

## Data Availability

Structural data were deposited in the PDB and are available under accession numbers 7BI2, 7BI4, 7BI6 and 7BI9 (X-ray crystallography) (Table 1) and EMD-12191 (cryo-EM) (Table 2). Original EM micrographs have been deposited in the EMPIAR and are available under the code EMPIAR-10665. All other data are available in the main manuscript, extended data, supplementary materials and in the source data. Materials and reagents are available from the corresponding authors upon request. Source data are provided with this paper.

## Source data

Source Data Fig. 2e Statistical source data for Fig. 2e.
Source Data Fig. 2f Statistical source data for Fig. 2f.
Source Data Fig. 5c Statistical source data for Fig. 5c.
Source Data Fig. 5e Statistical source data for Fig. 5e.
Source Data Fig. 5f Statistical source data for Fig. 5f.
Source Data Fig. 6a Statistical source data for Fig. 6a.
Source Data Fig. 6d Unprocessed blots for Fig. 6d.
Source Data Extended Data Fig. 1a Chromatography data for Extended Data Fig. 1a.
Source Data Extended Data Fig. 1b Uncropped gel for Extended Data Fig. 1b.
Source Data Extended Data Fig. 1c Statistical source data for Extended Data Fig. 1c.
Source Data Extended Data Fig. 3c Statistical source data for Extended Data Fig. 3c.
Source Data Extended Data Fig. 6d Statistical source data for Extended Data Fig. 6d.
Source Data Extended Data Fig. 6d Uncropped gel for Extended Data Fig. 6d.

## Extended data

are available for this paper at 10.1038/s41594-022-00730-w.
