# Supplementary material for: Structural basis of phosphatidylinositol 3-kinase C2α function
Source: Nat Struct Mol Biol. 2022 Mar 7;29(3):218–28. doi: 10.1038/s41594-022-00730-w (PMC8930771; doi:10.1038/s41594-022-00730-w)
Supplement: Supplementary file 1 — Supplementary Table 1 and PDB validation report. [file 41594_2022_730_MOESM1_ESM.pdf]

nature portfolio

https://doi.org/10.1038/s41594-022-00730-w

Supplementary information

Structural basis of phosphatidylinositol 3-kinase C2α function

In the format provided by the authors and unedited

Inventory of supplementary items contained in this file

Supplementary Table

Supplementary Table 1: Oligonucleotides

Supplementary Data

PDB validation report

Supplementary table 1. Oligonucleotides

Oligonucleotides	SOURCE	IDENTIFIER
Scrambled siRNA	Sigma	MISSION Universal Negative Control #1
PI3KC2α siRNA<br>GCACAAACCCAGGCUAUUU	Posor, et. al. 2013	
musPI3KC2a377_KasI_forward<br>GATCGGCGCCGTACAGAATGACGAGGTGGCAGCTTTTTG	This paper	
musPI3KC2a1539_XbaI_reverse<br>GATCTCTAGATTATTTCTCATCACGAAGTAAAGGGTGG	This paper	
musPI3KC2a1686_XbaI_reverse<br>GATCTCTAGATTATAGATACGTTGCCGCAGTCAGCTG	This paper	
musPI3KC2a1400_XbaI_reverse<br>GATCTCTAGATTAGCCAGAAAAACGTAGCTGAGCAAGGTTATG	This paper	
MusPI3KC2a_1409BamHI_forward<br>GATCGGATCCCTTTCATTCTCACCGAAAACATACTC	This paper	
MusPI3KC2a_1686NotI_reverse<br>GATCGCGGCCGCTTATAGATACGTTGCCGCAGTCAGCTG	This paper	
MusPI3KC2a_1539NotI_reverse<br>GATCGCGGCCGCTTATTTCTCATCACGAAGTAAAGGGTGG	This paper	
MusPI3KC2a1561_BamHI_forwar<br>GATCGGATCCGGAGCAGTGAAGTTATCTGTTTCTTAC	This paper	
MusPI3KC2a_533-544/GSGS_forward<br>AGAACCGTTTAAATCCACAGGTGCTTCATCATC	This paper	
MusPI3KC2a_533-544/GSGS_reverse<br>GGCTCTGTCATGACAAGACACCCTGTTGAAG	This paper	
musPI3KC2a1384_XbaI_ reverse<br>GATCTCTAGATTAGGCAATGCTTCCCAAACTTGACTCAATC	This paper	
musPI3KC2a550-665/SAGAGSGA_forward<br>CGCACCCGCAGAGTGTCTTGTCATGACAGAGCCAG	This paper	
musPI3KC2a550-665/SAGAGSGA _reverse<br>GGTAGTGCCGGTTCTACAGGCTGTCCCCGAGG	This paper	
musPI3KC2a545_EcoRI_ forward<br>GATCGAATTCGGCTCTGTCATGACAAGACAC	This paper	
musPI3KC2a668_NotI_ reverse<br>GATCGCGGCCGCTTAGCCTGTAGAACACCTACTAG	This paper	
hPI3KC2a1283-1284A2_forward<br>TGCGAAGCTGCCAAACATCTGTGC	This paper	
hPI3KC2a1283-1284A2_reverse<br>GCGGGATCGGGCTCCTTTTGT	This paper	
hPI3KC2a_H1391A_forward<br>TGCGAAGCTGCCAAACATCTGTGC	This paper	
hPI3KC2a_H1391A_reverse<br>GCGGGATCGGGCTCCTTTTGT	This paper	
mPI3KC2aK1283A_ forward<br>CGAAGCTACCAAACATCTGTGCATG	This paper	
mPI3KC2aK1283A_ reverse<br>CAAGGGACCGAGCTCCTTTTGTGC	This paper	
mPI3KC2a1283-1284A2_ forward<br>TGCGAAGCTACCAAACATCTGTGCATG	This paper	
mPI3KC2a1283-1284A2_ reverse<br>GCGGACCGAGCTCCTTTTGTGCTTAC	This paper	

musPI3KC2aR1284A_ forward<br>CTTTGAAGCTACCAAACATCTGTGC	This paper	
musPI3KC2aR1284A_ reverse<br>CGGACCGAGCTCCTTTTGTGC	This paper	
mPI3KC2a426_428A2_ forward<br>ACTGCCACACTGGCATTCTCCCCACAC	This paper	
mPI3KC2a426_428A2_ reverse<br>CGCCATTGAAATTGAAGGGCTTCAACT	This paper	
mPI3KC2a458_460_461A3_ forward<br>TACGGCGCAAAGGGCTTGCATTATAATTATTTCTAC	This paper	
mC2a458_460_461A3_ reverse<br>GCAGCTGACTTGAATCAAGTGGATGTTGGCAG	This paper	
hPI3KC2a426_428A2_forward<br>ACTGCCACACTAGCATTTTCTCCGCATATGT	This paper	
hPI3KC2a426_428A2_ reverse<br>CGCCATTGACATTGAAGGATTTCAGCTACC	This paper	
hPI3KC2a458_460_461A3_ forward<br>TACGGCGCAAAGGGCTTGCATTATAATGATTTCTAC	This paper	
hPI3C2a458_460_461A3_ reverse<br>GCAGCTGACTTGAATCAAGTAGATGTTGGCAG	This paper	
mC2a1391A_ forward<br>CAATGAAGAAATTAAACTTTGTGGCAATGCTTC	This paper	
mC2a1391A_ reverse<br>CTAACCTTGCTCAGCTACGTTTTTCTG	This paper	

Full wwPDB X-ray Structure Validation Report (i)

Jan 18, 2021 – 12:49 PM GMT

PDB ID	:	7BI2
Title	:	PI3KC2aDeltaN and DeltaC-C2
Deposited on	:	2021-01-12
Resolution	:	3.25 Å(reported)

CONFIDENTIAL VALIDATION REPORT

This is a Full wwPDB X-ray Structure Validation Report.

This report is produced by the wwPDB biocuration pipeline after annotation of the structure.

We welcome your comments at *validation@mail.wwpdb.org*
A user guide is available at
https://www.wwpdb.org/validation/2017/XrayValidationReportHelp
with specific help available everywhere you see the (i) symbol.

The following versions of software and data (see references (i)) were used in the production of this report:

MolProbity	:	4.02b-467
Mogul	:	1.8.5 (274361), CSD as541be (2020)
Xtriage (Phenix)	:	1.13
EDS	:	2.16
buster-report	:	1.1.7 (2018)
Percentile statistics	:	20191225.v01 (using entries in the PDB archive December 25th 2019)
Refmac	:	5.8.0158
CCP4	:	7.0.044 (Gargrove)
Ideal geometry (proteins)	:	Engh & Huber (2001)
Ideal geometry (DNA, RNA)	:	Parkinson et al. (1996)
Validation Pipeline (wwPDB-VP)	:	2.16

1 Overall quality at a glance (i)

The following experimental techniques were used to determine the structure:
X-RAY DIFFRACTION

The reported resolution of this entry is 3.25 Å.

Percentile scores (ranging between 0-100) for global validation metrics of the entry are shown in the following graphic. The table shows the number of entries on which the scores are based.

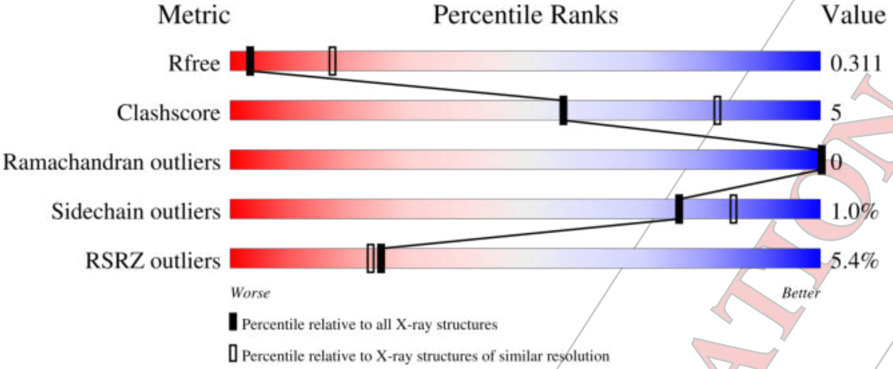

Metric	Whole archive (#Entries)	Similar resolution (#Entries, resolution range(Å))
R_{free}	130704	1191 (3.30-3.22)
Clashscore	141614	1251 (3.30-3.22)
Ramachandran outliers	138981	1229 (3.30-3.22)
Sidechain outliers	138945	1228 (3.30-3.22)
RSRZ outliers	127900	1154 (3.30-3.22)

The table below summarises the geometric issues observed across the polymeric chains and their fit to the electron density. The red, orange, yellow and green segments of the lower bar indicate the fraction of residues that contain outliers for >=3, 2, 1 and 0 types of geometric quality criteria respectively. A grey segment represents the fraction of residues that are not modelled. The numeric value for each fraction is indicated below the corresponding segment, with a dot representing fractions <=5% The upper red bar (where present) indicates the fraction of residues that have poor fit to the electron density. The numeric value is given above the bar.

Mol	Chain	Length	Quality of chain
1	A	1157	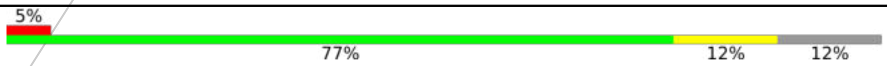

The following table lists non-polymeric compounds, carbohydrate monomers and non-standard residues in protein, DNA, RNA chains that are outliers for geometric or electron-density-fit criteria:

Mol	Type	Chain	Res	Chirality	Geometry	Clashes	Electron density
2	EDO	A	1201	-	-	-	X

Continued on next page...

CONFIDENTIAL VALIDATION REPORT

Continued from previous page...

Mol	Type	Chain	Res	Chirality	Geometry	Clashes	Electron density
3	17G	A	1202	-	-	-	X
4	IOD	A	1205	-	-	X	-
4	IOD	A	1206	-	-	X	-
4	IOD	A	1207	-	-	-	X

CONFIDENTIAL VALIDATION REPORT

2 Entry composition (i)

There are 5 unique types of molecules in this entry. The entry contains 8284 atoms, of which 0 are hydrogens and 0 are deuteriums.

In the tables below, the ZeroOcc column contains the number of atoms modelled with zero occupancy, the AltConf column contains the number of residues with at least one atom in alternate conformation and the Trace column contains the number of residues modelled with at most 2 atoms.

- Molecule 1 is a protein called Phosphatidylinositol 4-phosphate 3-kinase C2 domain-containing subunit alpha,Phosphatidylinositol 4-phosphate 3-kinase C2 domain-containing subunit alpha.

Mol	Chain	Residues	Atoms					ZeroOcc	AltConf	Trace
			Total	C	N	O	S			
1	A	1021	8224	5297	1388	1493	46	0	1	0

There are 7 discrepancies between the modelled and reference sequences:

Chain	Residue	Modelled	Actual	Comment	Reference
A	1	GLY	-	expression tag	UNP Q61194
A	2	ALA	-	expression tag	UNP Q61194
A	159	GLY	-	linker	UNP Q61194
A	160	SER	-	linker	UNP Q61194
A	161	GLY	-	linker	UNP Q61194
A	162	SER	-	linker	UNP Q61194
A	286	GLY	ALA	conflict	UNP Q61194

- Molecule 2 is 1,2-ETHANEDIOL (three-letter code: EDO) (formula: $C_2H_6O_2$).

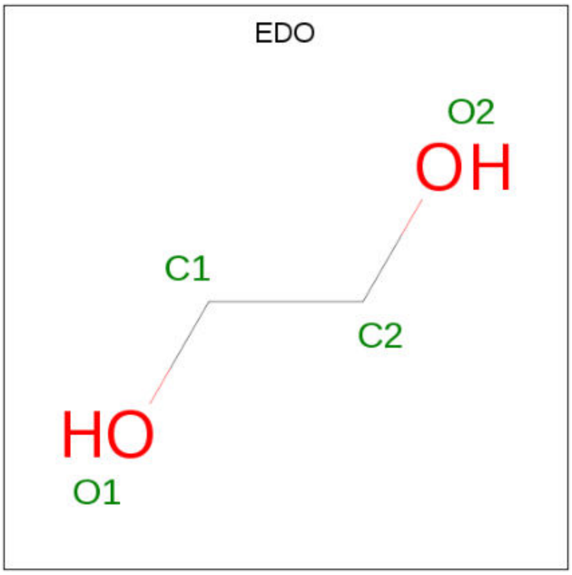

CONFIDENTIAL VALIDATION REPORT

Mol	Chain	Residues	Atoms			ZeroOcc	AltConf
			Total	C	O		
2	A	1	4	2	2	0	0

- Molecule 3 is 9-(6-aminopyridin-3-yl)-1-[3-(trifluoromethyl)phenyl]benzo[h][1,6]naphthyridin-2(1H)-one (three-letter code: 17G) (formula: $C_{24}H_{15}F_3N_4O$).

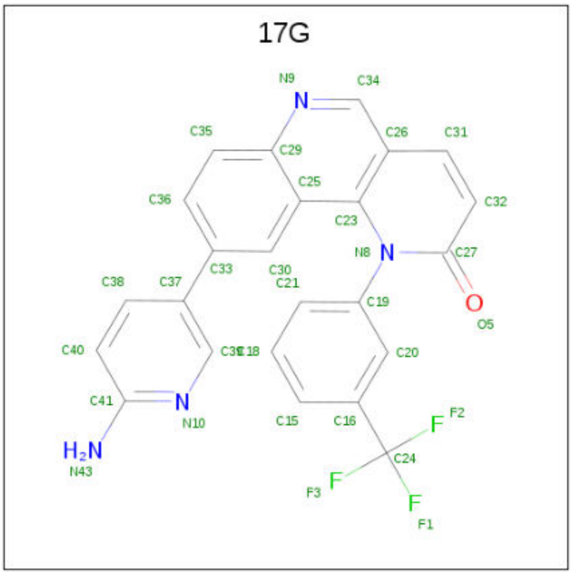

Mol	Chain	Residues	Atoms					ZeroOcc	AltConf
			Total	C	F	N	O		
3	A	1	32	24	3	4	1	0	0

- Molecule 4 is IODIDE ION (three-letter code: IOD) (formula: I).

Mol	Chain	Residues	Atoms		ZeroOcc	AltConf
			Total	I		
4	A	5	5	5	0	0

- Molecule 5 is water.

Mol	Chain	Residues	Atoms		ZeroOcc	AltConf
			Total	O		
5	A	19	19	19	0	0

CONFIDENTIAL VALIDATION REPORT

3 Residue-property plots (i)

These plots are drawn for all protein, RNA, DNA and oligosaccharide chains in the entry. The first graphic for a chain summarises the proportions of the various outlier classes displayed in the second graphic. The second graphic shows the sequence view annotated by issues in geometry and electron density. Residues are color-coded according to the number of geometric quality criteria for which they contain at least one outlier: green 0, yellow 1, orange 2 and red 3 or more. A red dot above a residue indicates a poor fit to the electron density (RSRZ > 2). Stretches of 2 or more consecutive residues without any outlier are shown as a green connector. Residues present in the sample, but not in the model, are shown in grey.

- Molecule 1: Phosphatidylinositol 4-phosphate 3-kinase C2 domain-containing subunit alpha,Phosphatidylinositol 4-phosphate 3-kinase C2 domain-containing subunit alpha

Chain A:

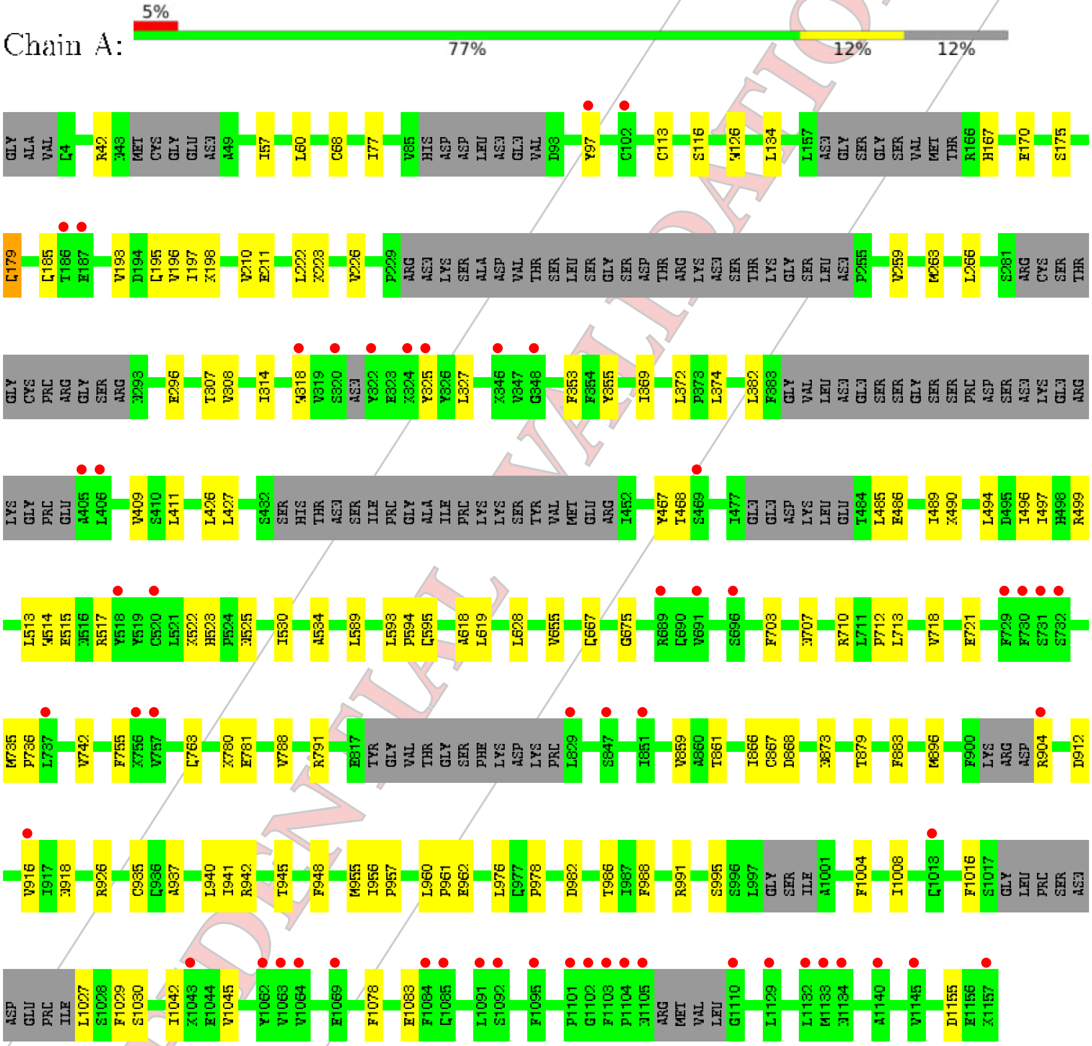

CONFIDENTIAL VALIDATION REPORT

4 Data and refinement statistics (i)

Property	Value	Source
Space group	P 21 21 21	Depositor
Cell constants<br>a, b, c, α, β, γ	82.56Å 115.89Å 144.37Å<br>90.00° 90.00° 90.00°	Depositor
Resolution (Å)	49.20 – 3.25<br>49.20 – 3.25	Depositor<br>EDS
% Data completeness<br>(in resolution range)	99.1 (49.20-3.25)<br>99.1 (49.20-3.25)	Depositor<br>EDS
R_{merge}	(Not available)	Depositor
R_{sym}	(Not available)	Depositor
$< I/\sigma(I) >$ [1]	1.15 (at 3.25Å)	Xtriage
Refinement program	REFMAC 5.8.0258	Depositor
R, R_{free}	0.266 , 0.312<br>0.263 , 0.311	Depositor<br>DCC
R_{free} test set	1113 reflections (5.00%)	wwPDB-VP
Wilson B-factor (Å^2)	109.8	Xtriage
Anisotropy	0.419	Xtriage
Bulk solvent k_{sol}(e/Å^3), B_{sol}(Å^2)	0.27 , 62.7	EDS
L-test for twinning[2]	$< \|L\| >$ = 0.47, $< L^2 >$ = 0.30	Xtriage
Estimated twinning fraction	No twinning to report.	Xtriage
F_o,F_c correlation	0.93	EDS
Total number of atoms	8284	wwPDB-VP
Average B, all atoms (Å^2)	137.0	wwPDB-VP

Xtriage's analysis on translational NCS is as follows: *The largest off-origin peak in the Patterson function is 3.92% of the height of the origin peak. No significant pseudotranslation is detected.*

[1] Intensities estimated from amplitudes.
[2] Theoretical values of $< |L| >$, $< L^2 >$ for acentric reflections are 0.5, 0.333 respectively for untwinned datasets, and 0.375, 0.2 for perfectly twinned datasets.

CONFIDENTIAL VALIDATION REPORT

5 Model quality (i)

5.1 Standard geometry (i)

Bond lengths and bond angles in the following residue types are not validated in this section: 17G, IOD, EDO

The Z score for a bond length (or angle) is the number of standard deviations the observed value is removed from the expected value. A bond length (or angle) with $|Z| > 5$ is considered an outlier worth inspection. RMSZ is the root-mean-square of all Z scores of the bond lengths (or angles).

Mol	Chain	Bond lengths		Bond angles	
		RMSZ	$\#|Z|>5$	RMSZ	$\#|Z|>5$
1	A	0.64	0/8400	0.70	0/11357

There are no bond length outliers.

There are no bond angle outliers.

There are no chirality outliers.

There are no planarity outliers.

5.2 Too-close contacts (i)

In the following table, the Non-H and H(model) columns list the number of non-hydrogen atoms and hydrogen atoms in the chain respectively. The H(added) column lists the number of hydrogen atoms added and optimized by MolProbity. The Clashes column lists the number of clashes within the asymmetric unit, whereas Symm-Clashes lists symmetry-related clashes.

Mol	Chain	Non-H	H(model)	H(added)	Clashes	Symm-Clashes
1	A	8224	0	8295	80	0
2	A	4	0	6	0	0
3	A	32	0	15	2	0
4	A	5	0	0	5	0
5	A	19	0	0	0	0
All	All	8284	0	8316	82	0

The all-atom clashscore is defined as the number of clashes found per 1000 atoms (including hydrogen atoms). The all-atom clashscore for this structure is 5.

All (82) close contacts within the same asymmetric unit are listed below, sorted by their clash magnitude.

CONFIDENTIAL VALIDATION REPORT

Atom-1	Atom-2	Interatomic distance (Å)	Clash overlap (Å)
1:A:307:THR:OG1	4:A:1203:IOD:I	2.74	0.75
1:A:60:LEU:O	4:A:1205:IOD:I	2.79	0.71
1:A:409:VAL:HG21	1:A:427:LEU:HB3	1.78	0.66
1:A:57:ILE:O	4:A:1205:IOD:I	2.84	0.65
1:A:523:HIS:HB3	4:A:1206:IOD:I	2.68	0.64
3:A:1202:17G:C19	3:A:1202:17G:H8	2.29	0.62
1:A:513:LEU:HD21	1:A:530:ILE:HG12	1.83	0.61
1:A:318:TRP:O	1:A:325:TYR:OH	2.21	0.58
1:A:867:CYS:SG	1:A:896:MET:HB2	2.44	0.58
1:A:525:ASN:HB2	4:A:1206:IOD:I	2.74	0.58
1:A:935:CYS:HB3	1:A:986:THR:HG22	1.86	0.57
1:A:222:LEU:HD22	1:A:266:LEU:HD11	1.87	0.57
1:A:593:LEU:N	1:A:594:PRO:HD2	2.20	0.56
1:A:918:ASN:HB2	1:A:926:ARG:HG3	1.88	0.56
1:A:514:TRP:CD1	1:A:534:ALA:HA	2.42	0.54
1:A:496:ILE:HA	1:A:499:ARG:HD3	1.89	0.54
1:A:995:SER:HB2	1:A:1008:ILE:HG13	1.88	0.54
1:A:961:PRO:O	1:A:962:GLU:HB2	2.07	0.54
1:A:374:LEU:HB2	1:A:467:TYR:CE2	2.42	0.54
1:A:411:LEU:HD12	1:A:427:LEU:HD21	1.89	0.53
1:A:193:VAL:HG21	1:A:226:VAL:HG21	1.91	0.53
1:A:486:GLU:HB2	1:A:489:ILE:HD11	1.91	0.53
1:A:763:GLN:HA	1:A:960:LEU:HD11	1.92	0.51
1:A:494:LEU:HA	1:A:497:ILE:HG12	1.93	0.50
1:A:42:ARG:NH1	1:A:780:LYS:O	2.44	0.50
1:A:496:ILE:HG22	1:A:513:LEU:HD12	1.92	0.50
1:A:113:CYS:SG	1:A:116:SER:HB2	2.51	0.50
1:A:942:ARG:O	1:A:945:THR:HG23	2.12	0.50
1:A:485:LEU:HB3	1:A:490:LYS:HE3	1.94	0.49
1:A:1027:LEU:HD23	1:A:1030:SER:HB3	1.94	0.49
1:A:327:LEU:HD23	1:A:382:LEU:HA	1.95	0.49
1:A:193:VAL:HG13	1:A:222:LEU:HD21	1.95	0.48
1:A:703:PHE:O	1:A:707:ASN:HB2	2.13	0.48
1:A:314:ILE:HG22	1:A:355:TYR:O	2.13	0.48
1:A:593:LEU:HD21	1:A:628:LEU:HG	1.96	0.48
1:A:68:CYS:SG	1:A:77:ILE:HG12	2.54	0.47
1:A:619:LEU:HD21	1:A:655:VAL:HG22	1.96	0.47
1:A:945:THR:HG22	1:A:976:LEU:HD13	1.97	0.46
1:A:861:THR:HA	1:A:866:ILE:CG2	2.46	0.46
1:A:982:ASP:O	1:A:986:THR:HG23	2.15	0.45
1:A:618:ALA:HB2	1:A:628:LEU:HD12	1.99	0.45
1:A:226:VAL:HG22	1:A:259:VAL:HG22	1.97	0.45

Continued on next page...

CONFIDENTIAL VALIDATION REPORT

Continued from previous page...

Atom-1	Atom-2	Interatomic distance (Å)	Clash overlap (Å)
1:A:60:LEU:HD12	1:A:60:LEU:N	2.31	0.45
1:A:496:ILE:CG2	1:A:513:LEU:HD12	2.47	0.45
1:A:721:GLU:O	1:A:742:VAL:HG12	2.16	0.45
1:A:1004:PHE:O	1:A:1008:ILE:HG12	2.16	0.45
1:A:175:SER:O	1:A:179:GLN:NE2	2.50	0.45
1:A:781:GLU:HG3	1:A:940:LEU:HD22	1.98	0.45
1:A:515:GLU:OE1	1:A:515:GLU:HA	2.16	0.44
1:A:713:LEU:HD21	1:A:791:ARG:HB2	2.00	0.44
1:A:912:ASP:O	1:A:916:VAL:HG23	2.17	0.44
1:A:195:GLN:HA	1:A:198:LYS:HD2	2.00	0.43
1:A:222:LEU:HD22	1:A:266:LEU:CD1	2.48	0.43
1:A:589:LEU:CD2	1:A:593:LEU:HD13	2.49	0.43
1:A:514:TRP:O	1:A:517:ARG:HB2	2.19	0.43
1:A:988:PHE:O	1:A:991:ARG:HG2	2.19	0.43
1:A:308:VAL:HG23	1:A:308:VAL:O	2.20	0.42
1:A:595:GLN:NE2	1:A:955:MET:SD	2.92	0.42
1:A:1042:ILE:HG21	1:A:1045:VAL:HG23	2.01	0.42
1:A:1078:PHE:HD1	1:A:1155:ASP:HA	1.84	0.42
1:A:859:VAL:HG21	1:A:937:ALA:HB3	2.01	0.42
1:A:941:ILE:HG23	1:A:948:PHE:CE1	2.55	0.42
1:A:945:THR:HG21	1:A:978:PRO:HG3	2.00	0.42
1:A:788:VAL:HB	1:A:883:PHE:HB3	2.01	0.42
1:A:956:ILE:N	1:A:957:PRO:CD	2.83	0.42
1:A:211:GLU:OE1	1:A:353:PHE:HB2	2.20	0.41
1:A:735:MET:N	1:A:736:PRO:HD3	2.35	0.41
1:A:513:LEU:CD2	1:A:530:ILE:HG12	2.49	0.41
1:A:861:THR:HA	1:A:866:ILE:HG22	2.01	0.41
1:A:868:ASP:O	1:A:873:ASN:ND2	2.53	0.41
1:A:522:LYS:HG3	1:A:523:HIS:CD2	2.55	0.41
1:A:97:TYR:HB3	1:A:134:LEU:HD11	2.03	0.41
1:A:210:VAL:HG11	1:A:426:LEU:HD21	2.03	0.41
1:A:1029:PHE:CD1	1:A:1083:GLU:HG2	2.56	0.41
1:A:197:ILE:HD13	1:A:223:LYS:HE2	2.02	0.41
1:A:296:GLU:OE2	1:A:468:THR:OG1	2.37	0.41
1:A:710:ARG:HG2	1:A:718:VAL:HG22	2.02	0.41
1:A:667:GLN:HE21	1:A:712:PRO:HA	1.85	0.40
3:A:1202:17G:H9	3:A:1202:17G:H11	1.83	0.40
1:A:369:ILE:O	1:A:372:LEU:HB2	2.22	0.40
1:A:675:GLY:HA2	1:A:755:PHE:HE2	1.86	0.40
1:A:193:VAL:O	1:A:196:VAL:HB	2.21	0.40

There are no symmetry-related clashes.

CONFIDENTIAL VALIDATION REPORT

5.3 Torsion angles (i)

5.3.1 Protein backbone (i)

In the following table, the Percentiles column shows the percent Ramachandran outliers of the chain as a percentile score with respect to all X-ray entries followed by that with respect to entries of similar resolution.

The Analysed column shows the number of residues for which the backbone conformation was analysed, and the total number of residues.

Mol	Chain	Analysed	Favoured	Allowed	Outliers	Percentiles
1	A	992/1157 (86%)	945 (95%)	47 (5%)	0	100 100

There are no Ramachandran outliers to report.

5.3.2 Protein sidechains (i)

In the following table, the Percentiles column shows the percent sidechain outliers of the chain as a percentile score with respect to all X-ray entries followed by that with respect to entries of similar resolution.

The Analysed column shows the number of residues for which the sidechain conformation was analysed, and the total number of residues.

Mol	Chain	Analysed	Rotameric	Outliers	Percentiles
1	A	920/1036 (89%)	911 (99%)	9 (1%)	76 85

All (9) residues with a non-rotameric sidechain are listed below:

Mol	Chain	Res	Type
1	A	126	TRP
1	A	167	HIS
1	A	170	GLU
1	A	179	GLN
1	A	185	GLN
1	A	263	MET
1	A	879	THR
1	A	904	ARG
1	A	1016	PHE

Sometimes sidechains can be flipped to improve hydrogen bonding and reduce clashes. All (3) such sidechains are listed below:

CONFIDENTIAL VALIDATION REPORT

Mol	Chain	Res	Type
1	A	109	GLN
1	A	523	HIS
1	A	623	GLN

5.3.3 RNA (i)

There are no RNA molecules in this entry.

5.4 Non-standard residues in protein, DNA, RNA chains (i)

There are no non-standard protein/DNA/RNA residues in this entry.

5.5 Carbohydrates (i)

There are no monosaccharides in this entry.

5.6 Ligand geometry (i)

Of 7 ligands modelled in this entry, 5 are monoatomic - leaving 2 for Mogul analysis.

In the following table, the Counts columns list the number of bonds (or angles) for which Mogul statistics could be retrieved, the number of bonds (or angles) that are observed in the model and the number of bonds (or angles) that are defined in the Chemical Component Dictionary. The Link column lists molecule types, if any, to which the group is linked. The Z score for a bond length (or angle) is the number of standard deviations the observed value is removed from the expected value. A bond length (or angle) with $|Z| > 2$ is considered an outlier worth inspection. RMSZ is the root-mean-square of all Z scores of the bond lengths (or angles).

Mol	Type	Chain	Res	Link	Bond lengths			Bond angles		
					Counts	RMSZ	$\#\|Z\| > 2$	Counts	RMSZ	$\#\|Z\| > 2$
3	17G	A	1202	-	35,36,36	2.90	8 (22%)	49,54,54	1.92	8 (16%)
2	EDO	A	1201	-	3,3,3	0.08	0	2,2,2	0.22	0

In the following table, the Chirals column lists the number of chiral outliers, the number of chiral centers analysed, the number of these observed in the model and the number defined in the Chemical Component Dictionary. Similar counts are reported in the Torsion and Rings columns. '-' means no outliers of that kind were identified.

Mol	Type	Chain	Res	Link	Chirals	Torsions	Rings
3	17G	A	1202	-	-	0/14/14/14	0/5/5/5
2	EDO	A	1201	-	-	0/1/1/1	-

CONFIDENTIAL VALIDATION REPORT

All (8) bond length outliers are listed below:

Mol	Chain	Res	Type	Atoms	Z	Observed(Å)	Ideal(Å)
3	A	1202	17G	C23-N8	11.56	1.57	1.38
3	A	1202	17G	C27-N8	5.60	1.47	1.37
3	A	1202	17G	C31-C26	5.25	1.54	1.41
3	A	1202	17G	C34-N9	5.06	1.36	1.30
3	A	1202	17G	C31-C32	4.88	1.46	1.36
3	A	1202	17G	C41-N43	3.67	1.46	1.35
3	A	1202	17G	C37-C33	3.54	1.57	1.49
3	A	1202	17G	O5-C27	-2.07	1.19	1.24

All (8) bond angle outliers are listed below:

Mol	Chain	Res	Type	Atoms	Z	Observed(^{o})	Ideal(^{o})
3	A	1202	17G	C36-C33-C37	-6.11	110.77	121.36
3	A	1202	17G	C19-N8-C23	4.97	124.38	118.76
3	A	1202	17G	C30-C33-C37	4.24	130.73	121.05
3	A	1202	17G	C27-N8-C19	-3.96	114.54	118.69
3	A	1202	17G	C31-C26-C34	-3.77	115.87	122.63
3	A	1202	17G	C26-C34-N9	-3.64	120.10	125.05
3	A	1202	17G	C31-C26-C23	2.96	122.75	117.94
3	A	1202	17G	C37-C39-N10	-2.11	120.84	124.32

There are no chirality outliers.

There are no torsion outliers.

There are no ring outliers.

1 monomer is involved in 2 short contacts:

Mol	Chain	Res	Type	Clashes	Symm-Clashes
3	A	1202	17G	2	0

The following is a two-dimensional graphical depiction of Mogul quality analysis of bond lengths, bond angles, torsion angles, and ring geometry for all instances of the Ligand of Interest. In addition, ligands with molecular weight > 250 and outliers as shown on the validation Tables will also be included. For torsion angles, if less then 5% of the Mogul distribution of torsion angles is within 10 degrees of the torsion angle in question, then that torsion angle is considered an outlier. Any bond that is central to one or more torsion angles identified as an outlier by Mogul will be highlighted in the graph. For rings, the root-mean-square deviation (RMSD) between the ring in question and similar rings identified by Mogul is calculated over all ring torsion angles. If the average RMSD is greater than 60 degrees and the minimal RMSD between the ring in question and any Mogul-identified rings is also greater than 60 degrees, then that ring is considered an outlier. The outliers are highlighted in purple. The color gray indicates Mogul did not find sufficient

CONFIDENTIAL VALIDATION REPORT

equivalents in the CSD to analyse the geometry.

Ligand 17G A 1202

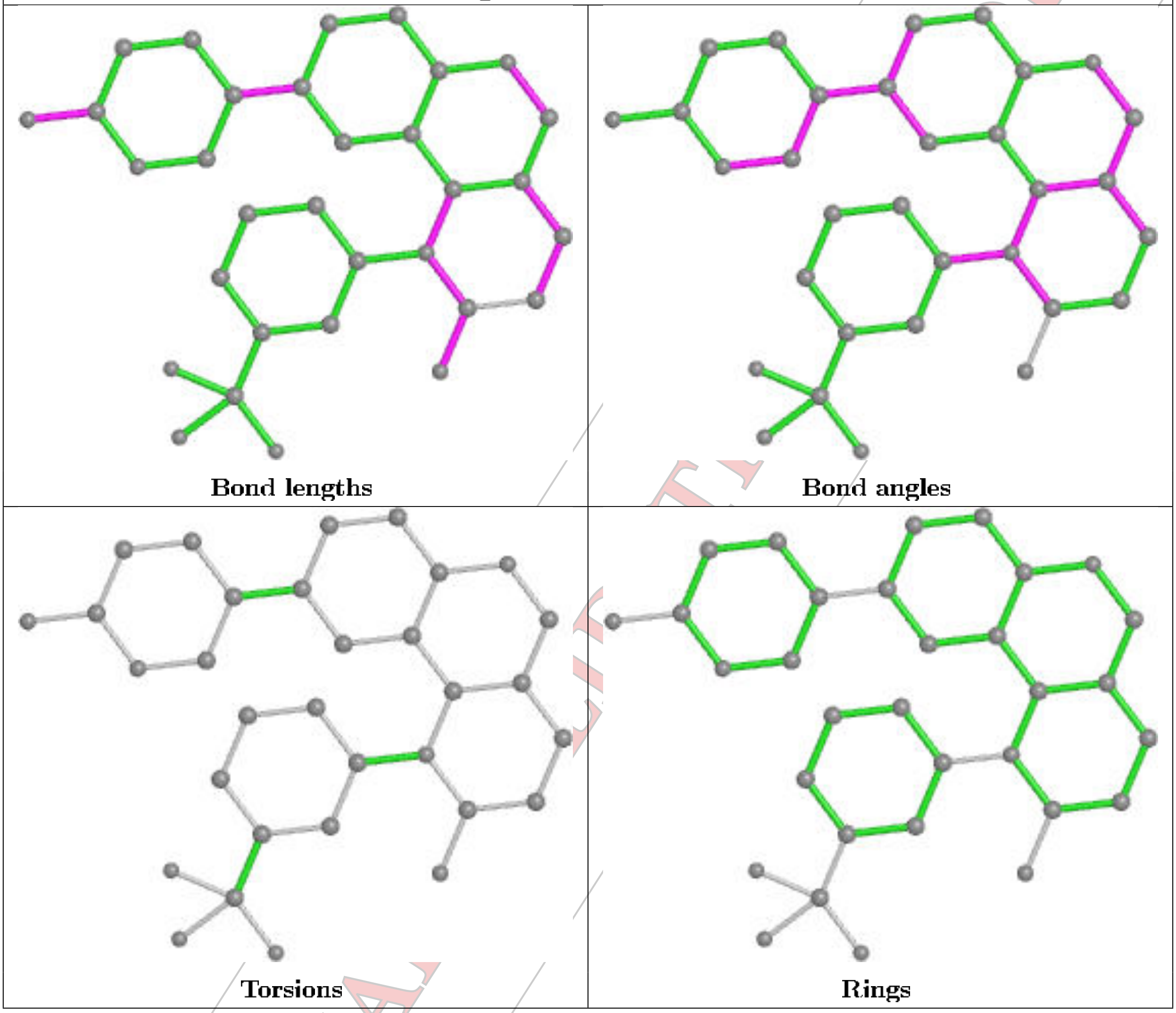

5.7 Other polymers (i)

There are no such residues in this entry.

5.8 Polymer linkage issues (i)

There are no chain breaks in this entry.

CONFIDENTIAL REPORT

6 Fit of model and data (i)

6.1 Protein, DNA and RNA chains (i)

In the following table, the column labelled '#RSRZ> 2' contains the number (and percentage) of RSRZ outliers, followed by percent RSRZ outliers for the chain as percentile scores relative to all X-ray entries and entries of similar resolution. The OWAB column contains the minimum, median, 95th percentile and maximum values of the occupancy-weighted average B-factor per residue. The column labelled 'Q< 0.9' lists the number of (and percentage) of residues with an average occupancy less than 0.9.

Mol	Chain	Analysed	<RSRZ>	#RSRZ>2			OWAB(Å^2)	Q<0.9
1	A	1021/1157 (88%)	0.18	55 (5%)	25	24	76, 130, 193, 268	0

All (55) RSRZ outliers are listed below:

Mol	Chain	Res	Type	RSRZ
1	A	1103	PHE	5.6
1	A	1110	GLY	4.7
1	A	1102	GLY	4.6
1	A	1133	MET	4.0
1	A	737	LEU	3.9
1	A	1091	LEU	3.8
1	A	520	CYS	3.7
1	A	732	SER	3.7
1	A	1104	PRO	3.7
1	A	186	THR	3.6
1	A	916	VAL	3.4
1	A	1134	ASN	3.4
1	A	1084	PHE	3.4
1	A	689	ARG	3.4
1	A	325	TYR	3.3
1	A	1101	PRO	3.3
1	A	187	GLU	3.2
1	A	756	LYS	3.1
1	A	1043	LYS	2.9
1	A	469	SER	2.9
1	A	1092	SER	2.9
1	A	322	TYR	2.9
1	A	829	LEU	2.9
1	A	1095	PHE	2.9
1	A	1069	GLU	2.7
1	A	1062	TYR	2.7
1	A	1063	VAL	2.7

Continued on next page...

CONFIDENTIAL VALIDATION REPORT

Continued from previous page...

Mol	Chain	Res	Type	RSRZ
1	A	904	ARG	2.7
1	A	1129	LEU	2.7
1	A	1132	LEU	2.7
1	A	851	ILE	2.6
1	A	757	VAL	2.6
1	A	405	ALA	2.6
1	A	1140	ALA	2.6
1	A	1064	VAL	2.5
1	A	324	LYS	2.5
1	A	731	SER	2.5
1	A	320	SER	2.5
1	A	1105	ASN	2.5
1	A	346	LYS	2.4
1	A	406	LEU	2.4
1	A	348	GLY	2.3
1	A	696	SER	2.2
1	A	729	PHE	2.2
1	A	1085	GLN	2.2
1	A	97	TYR	2.1
1	A	518	TYR	2.1
1	A	691	VAL	2.1
1	A	730	PHE	2.1
1	A	1157	LYS	2.1
1	A	318	TRP	2.1
1	A	1145	VAL	2.0
1	A	102	CYS	2.0
1	A	1013	GLN	2.0
1	A	847	SER	2.0

6.2 Non-standard residues in protein, DNA, RNA chains (i)

There are no non-standard protein/DNA/RNA residues in this entry.

6.3 Carbohydrates (i)

There are no monosaccharides in this entry.

6.4 Ligands (i)

In the following table, the Atoms column lists the number of modelled atoms in the group and the number defined in the chemical component dictionary. The B-factors column lists the minimum,

CONFIDENTIAL VALIDATION REPORT

median, 95th percentile and maximum values of B factors of atoms in the group. The column labelled 'Q< 0.9' lists the number of atoms with occupancy less than 0.9.

Mol	Type	Chain	Res	Atoms	RSCC	RSR	B-factors(Å^2)	Q<0.9
4	IOD	A	1204	1/1	0.40	0.22	296,296,296,296	0
4	IOD	A	1206	1/1	0.43	0.19	336,336,336,336	0
2	EDO	A	1201	4/4	0.67	0.49	155,166,171,171	0
4	IOD	A	1207	1/1	0.68	1.01	545,545,545,545	0
4	IOD	A	1205	1/1	0.71	0.22	324,324,324,324	0
3	17G	A	1202	32/32	0.76	0.53	190,221,241,250	0
4	IOD	A	1203	1/1	0.82	0.32	278,278,278,278	0

The following is a graphical depiction of the model fit to experimental electron density of all instances of the Ligand of Interest. In addition, ligands with molecular weight > 250 and outliers as shown on the geometry validation Tables will also be included. Each fit is shown from different orientation to approximate a three-dimensional view.

Electron density around 17G A 1202:

2mF$_o$-DF$_c$ (at 0.7 rmsd) in gray
mF$_o$-DF$_c$ (at 3 rmsd) in purple (negative)
and green (positive)

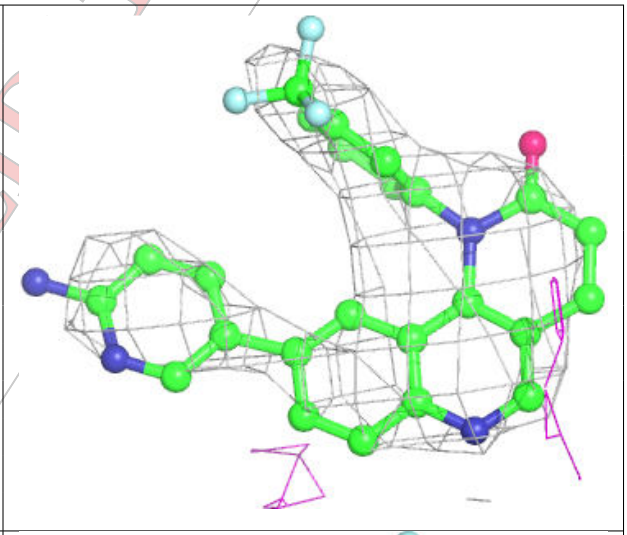

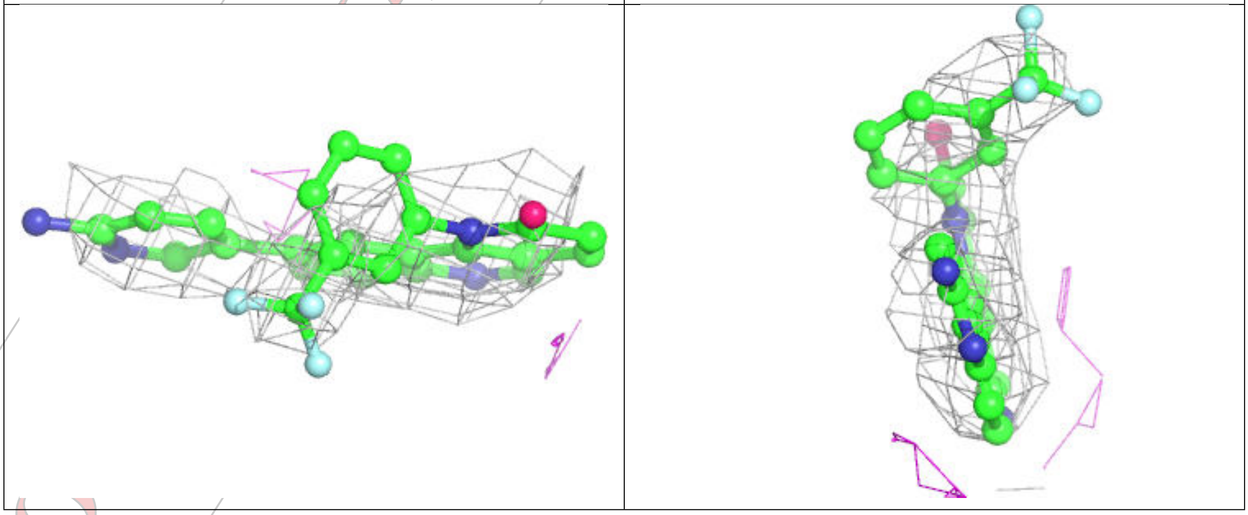

6.5 Other polymers (i)

There are no such residues in this entry.

CONFIDENTIAL VALIDATION REPORT

Full wwPDB X-ray Structure Validation Report (i)

Jan 14, 2021 – 05:31 PM GMT

PDB ID	:	7BI4
Title	:	PI3KC2a core apo
Deposited on	:	2021-01-12
Resolution	:	2.42 Å(reported)

This is a Full wwPDB X-ray Structure Validation Report.

This report is produced by the wwPDB biocuration pipeline after annotation of the structure.

We welcome your comments at *validation@mail.wwpdb.org*
A user guide is available at
https://www.wwpdb.org/validation/2017/XrayValidationReportHelp
with specific help available everywhere you see the (i) symbol.

CONFIDENTIAL VALIDATION REPORT

The following versions of software and data (see references (i)) were used in the production of this report:

MolProbity	:	4.02b-467
Mogul	:	1.8.5 (274361), CSD as541be (2020)
Xtriage (Phenix)	:	1.13
EDS	:	2.16
Percentile statistics	:	20191225.v01 (using entries in the PDB archive December 25th 2019)
Refmac	:	5.8.0158
CCP4	:	7.0.044 (Gargrove)
Ideal geometry (proteins)	:	Engh & Huber (2001)
Ideal geometry (DNA, RNA)	:	Parkinson et al. (1996)
Validation Pipeline (wwPDB-VP)	:	2.16

1 Overall quality at a glance (i)

The following experimental techniques were used to determine the structure:
X-RAY DIFFRACTION

The reported resolution of this entry is 2.42 Å.

Percentile scores (ranging between 0-100) for global validation metrics of the entry are shown in the following graphic. The table shows the number of entries on which the scores are based.

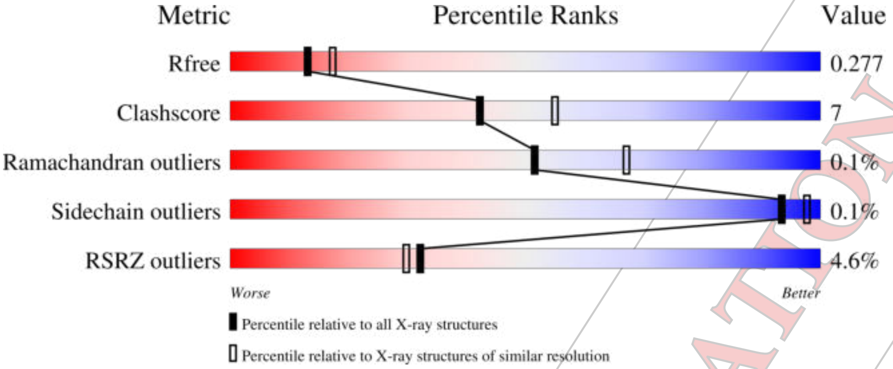

Metric	Whole archive (#Entries)	Similar resolution (#Entries, resolution range(Å))
R_{free}	130704	4647 (2.44-2.40)
Clashscore	141614	5161 (2.44-2.40)
Ramachandran outliers	138981	5073 (2.44-2.40)
Sidechain outliers	138945	5074 (2.44-2.40)
RSRZ outliers	127900	4543 (2.44-2.40)

The table below summarises the geometric issues observed across the polymeric chains and their fit to the electron density. The red, orange, yellow and green segments of the lower bar indicate the fraction of residues that contain outliers for >=3, 2, 1 and 0 types of geometric quality criteria respectively. A grey segment represents the fraction of residues that are not modelled. The numeric value for each fraction is indicated below the corresponding segment, with a dot representing fractions <=5% The upper red bar (where present) indicates the fraction of residues that have poor fit to the electron density. The numeric value is given above the bar.

Mol	Chain	Length	Quality of chain
1	A	910	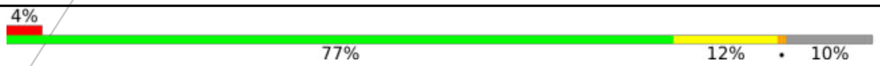

CONFIDENTIAL VALIDATION REPORT

2 Entry composition (i)

There are 4 unique types of molecules in this entry. The entry contains 6652 atoms, of which 0 are hydrogens and 0 are deuteriums.

In the tables below, the ZeroOcc column contains the number of atoms modelled with zero occupancy, the AltConf column contains the number of residues with at least one atom in alternate conformation and the Trace column contains the number of residues modelled with at most 2 atoms.

- Molecule 1 is a protein called Phosphatidylinositol 4-phosphate 3-kinase C2 domain-containing subunit alpha,Phosphatidylinositol 4-phosphate 3-kinase C2 domain-containing subunit alpha.

Mol	Chain	Residues	Atoms					ZeroOcc	AltConf	Trace
			Total	C	N	O	S			
1	A	822	6554	4209	1100	1204	41	0	2	0

There are 23 discrepancies between the modelled and reference sequences:

Chain	Residue	Modelled	Actual	Comment	Reference
A	1	GLY	-	expression tag	UNP Q61194
A	2	ALA	-	expression tag	UNP Q61194
A	267	GLY	-	linker	UNP Q61194
A	268	SER	-	linker	UNP Q61194
A	269	GLY	-	linker	UNP Q61194
A	270	SER	-	linker	UNP Q61194
A	271	VAL	-	linker	UNP Q61194
A	272	MET	-	linker	UNP Q61194
A	273	THR	-	linker	UNP Q61194
A	274	ARG	-	linker	UNP Q61194
A	275	HIS	-	linker	UNP Q61194
A	276	SER	-	linker	UNP Q61194
A	277	ALA	-	linker	UNP Q61194
A	278	GLY	-	linker	UNP Q61194
A	279	ALA	-	linker	UNP Q61194
A	280	GLY	-	linker	UNP Q61194
A	281	SER	-	linker	UNP Q61194
A	282	GLY	-	linker	UNP Q61194
A	283	ALA	-	linker	UNP Q61194
A	286	GLY	ALA	conflict	UNP Q61194
A	353	ALA	PHE	engineered mutation	UNP Q61194
A	354	ALA	PHE	engineered mutation	UNP Q61194
A	427	ALA	LEU	engineered mutation	UNP Q61194

- Molecule 2 is 1,2-ETHANEDIOL (three-letter code: EDO) (formula: $C_2H_6O_2$).

CONFIDENTIAL VALIDATION REPORT

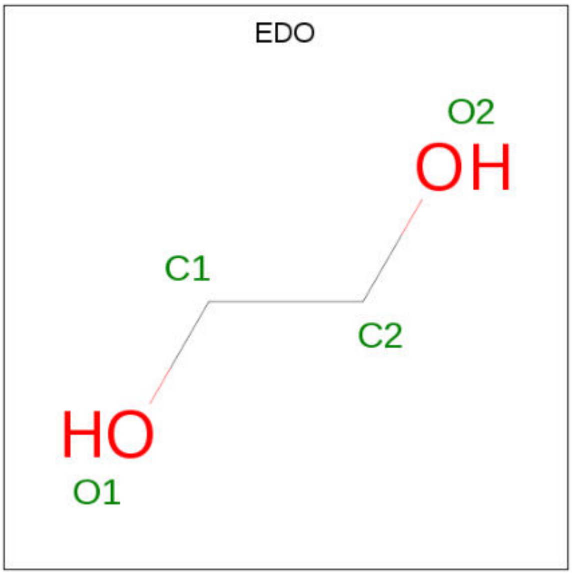

Mol	Chain	Residues	Atoms	ZeroOcc	AltConf
2	A	1	Total C O<br>4 2 2	0	0
2	A	1	Total C O<br>4 2 2	0	0

- Molecule 3 is SULFATE ION (three-letter code: SO4) (formula: O_4S).

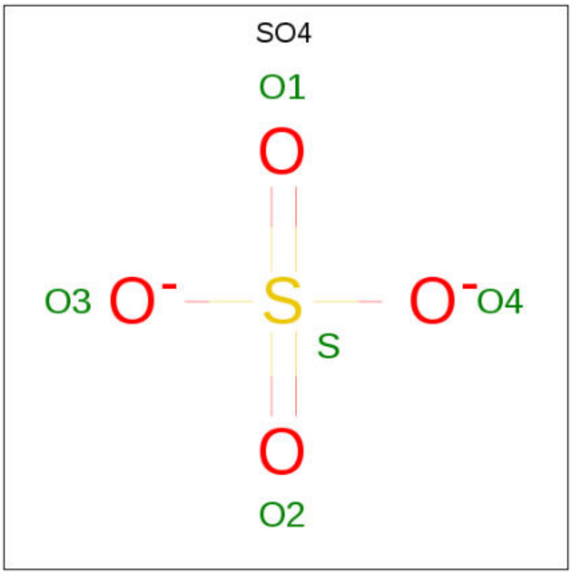

Mol	Chain	Residues	Atoms	ZeroOcc	AltConf
3	A	1	Total O S<br>5 4 1	0	0

- Molecule 4 is water.

CONFIDENTIAL VALIDATION REPORT

Mol	Chain	Residues	Atoms		ZeroOcc	AltConf
			Total	O		
4	A	85	85	85	0	0

CONFIDENTIAL VALIDATION REPORT

3 Residue-property plots (i)

These plots are drawn for all protein, RNA, DNA and oligosaccharide chains in the entry. The first graphic for a chain summarises the proportions of the various outlier classes displayed in the second graphic. The second graphic shows the sequence view annotated by issues in geometry and electron density. Residues are color-coded according to the number of geometric quality criteria for which they contain at least one outlier: green 0, yellow 1, orange 2 and red 3 or more. A red dot above a residue indicates a poor fit to the electron density (RSRZ > 2). Stretches of 2 or more consecutive residues without any outlier are shown as a green connector. Residues present in the sample, but not in the model, are shown in grey.

- Molecule 1: Phosphatidylinositol 4-phosphate 3-kinase C2 domain-containing subunit alpha,Phosphatidylinositol 4-phosphate 3-kinase C2 domain-containing subunit alpha

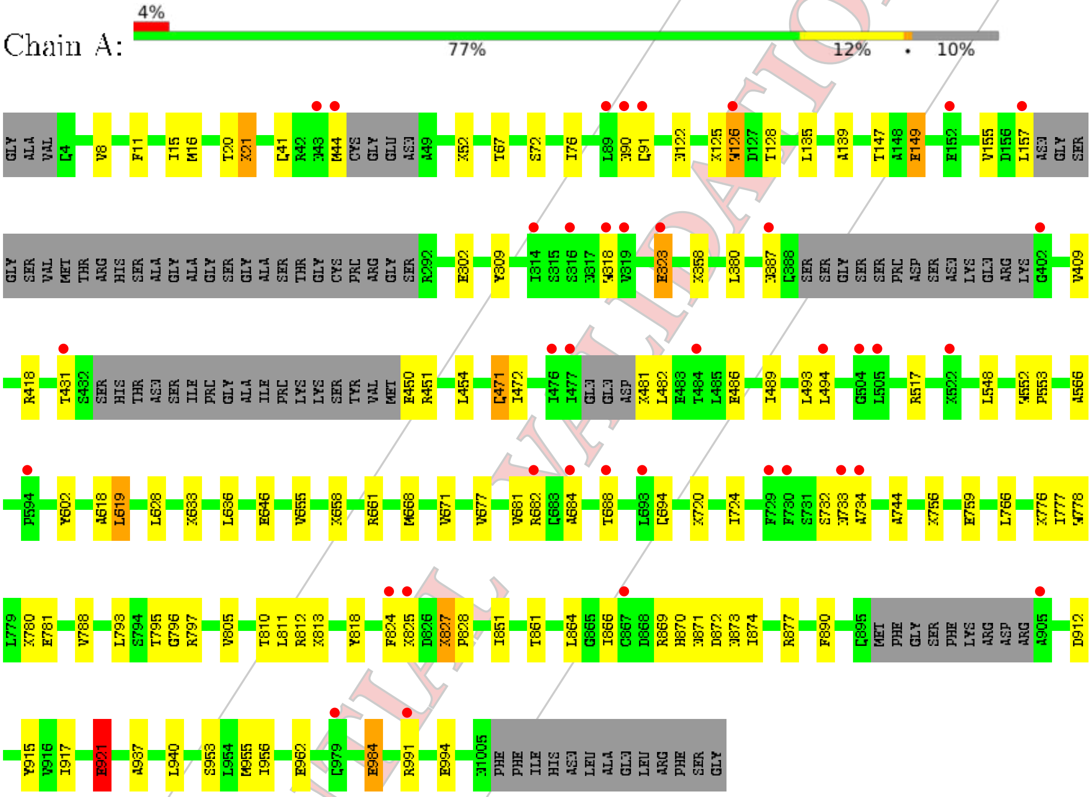

CONFIDENTIAL VALIDATION REPORT

4 Data and refinement statistics (i)

Property	Value	Source
Space group	P 21 21 21	Depositor
Cell constants<br>a, b, c, α, β, γ	135.19Å 151.57Å 56.04Å<br>90.00° 90.00° 90.00°	Depositor
Resolution (Å)	45.06 – 2.42<br>45.06 – 2.42	Depositor<br>EDS
% Data completeness<br>(in resolution range)	98.7 (45.06-2.42)<br>98.7 (45.06-2.42)	Depositor<br>EDS
R_{merge}	(Not available)	Depositor
R_{sym}	(Not available)	Depositor
$< I/\sigma(I) >$ [1]	1.07 (at 2.42Å)	Xtriage
Refinement program	PHENIX 1.18.2_3874	Depositor
R, R_{free}	0.229 , 0.277<br>0.230 , 0.277	Depositor<br>DCC
R_{free} test set	2100 reflections (4.72%)	wwPDB-VP
Wilson B-factor (Å^2)	61.1	Xtriage
Anisotropy	0.297	Xtriage
Bulk solvent k_{sol}(e/Å^3), B_{sol}(Å^2)	0.30 , 41.1	EDS
L-test for twinning[2]	$< \|L\| >$ = 0.50, $< L^2 >$ = 0.34	Xtriage
Estimated twinning fraction	No twinning to report.	Xtriage
F_o,F_c correlation	0.95	EDS
Total number of atoms	6652	wwPDB-VP
Average B, all atoms (Å^2)	71.0	wwPDB-VP

Xtriage's analysis on translational NCS is as follows: *The largest off-origin peak in the Patterson function is 4.59% of the height of the origin peak. No significant pseudotranslation is detected.*

[1] Intensities estimated from amplitudes.
[2] Theoretical values of $< |L| >$, $< L^2 >$ for acentric reflections are 0.5, 0.333 respectively for untwinned datasets, and 0.375, 0.2 for perfectly twinned datasets.

CONFIDENTIAL VALIDATION REPORT

5 Model quality (i)

5.1 Standard geometry (i)

Bond lengths and bond angles in the following residue types are not validated in this section: SO4, EDO

The Z score for a bond length (or angle) is the number of standard deviations the observed value is removed from the expected value. A bond length (or angle) with $|Z| > 5$ is considered an outlier worth inspection. RMSZ is the root-mean-square of all Z scores of the bond lengths (or angles).

Mol	Chain	Bond lengths		Bond angles	
		RMSZ	#|Z| >5	RMSZ	#|Z| >5
1	A	0.42	6/6693 (0.1%)	0.74	14/9057 (0.2%)

Chiral center outliers are detected by calculating the chiral volume of a chiral center and verifying if the center is modelled as a planar moiety or with the opposite hand.A planarity outlier is detected by checking planarity of atoms in a peptide group, atoms in a mainchain group or atoms of a sidechain that are expected to be planar.

Mol	Chain	#Chirality outliers	#Planarity outliers
1	A	0	2

All (6) bond length outliers are listed below:

Mol	Chain	Res	Type	Atoms	Z	Observed(Å)	Ideal(Å)
1	A	921	GLU	CD-OE2	6.46	1.32	1.25
1	A	302	GLU	CD-OE1	6.39	1.32	1.25
1	A	984	GLU	CD-OE2	5.56	1.31	1.25
1	A	149	GLU	CD-OE2	5.26	1.31	1.25
1	A	21	LYS	CE-NZ	-5.05	1.36	1.49
1	A	302	GLU	CB-CG	5.02	1.61	1.52

All (14) bond angle outliers are listed below:

Mol	Chain	Res	Type	Atoms	Z	Observed(^{o})	Ideal(^{o})
1	A	921	GLU	OE1-CD-OE2	-21.48	97.52	123.30
1	A	984	GLU	OE1-CD-OE2	-20.22	99.03	123.30
1	A	921	GLU	CG-CD-OE1	12.08	142.46	118.30
1	A	984	GLU	CG-CD-OE1	11.13	140.56	118.30
1	A	921	GLU	CG-CD-OE2	-8.85	100.61	118.30
1	A	984	GLU	CG-CD-OE2	-7.99	102.32	118.30
1	A	454	LEU	CB-CG-CD2	-7.92	97.53	111.00

Continued on next page...

CONFIDENTIAL VALIDATION REPORT

Continued from previous page...

Mol	Chain	Res	Type	Atoms	Z	Observed(°)	Ideal(°)
1	A	450	GLU	N-CA-C	-7.53	90.67	111.00
1	A	619	LEU	CB-CG-CD2	-7.27	98.64	111.00
1	A	126	TRP	N-CA-CB	-7.01	97.99	110.60
1	A	21	LYS	CB-CG-CD	-6.81	93.88	111.60
1	A	323	GLU	CA-CB-CG	6.57	127.86	113.40
1	A	418	ARG	CB-CG-CD	5.91	126.98	111.60
1	A	471	GLN	CA-CB-CG	-5.04	102.31	113.40

There are no chirality outliers.

All (2) planarity outliers are listed below:

Mol	Chain	Res	Type	Group
1	A	921	GLU	Sidechain
1	A	984	GLU	Sidechain

5.2 Too-close contacts (i)

In the following table, the Non-H and H(model) columns list the number of non-hydrogen atoms and hydrogen atoms in the chain respectively. The H(added) column lists the number of hydrogen atoms added and optimized by MolProbity. The Clashes column lists the number of clashes within the asymmetric unit, whereas Symm-Clashes lists symmetry-related clashes.

Mol	Chain	Non-H	H(model)	H(added)	Clashes	Symm-Clashes
1	A	6554	0	6624	91	0
2	A	8	0	12	1	0
3	A	5	0	0	0	0
4	A	85	0	0	3	0
All	All	6652	0	6636	91	0

The all-atom clashscore is defined as the number of clashes found per 1000 atoms (including hydrogen atoms). The all-atom clashscore for this structure is 7.

All (91) close contacts within the same asymmetric unit are listed below, sorted by their clash magnitude.

Atom-1	Atom-2	Interatomic distance (Å)	Clash overlap (Å)
1:A:658:LYS:HE2	1:A:661:ARG:HH21	1.36	0.89
1:A:52:LYS:HD3	1:A:67:THR:HG22	1.60	0.84
1:A:619:LEU:HD21	1:A:655:VAL:HG22	1.65	0.79
1:A:126:TRP:HB3	1:A:128:THR:HG23	1.68	0.75

Continued on next page...

CONFIDENTIAL VALIDATION REPORT

Continued from previous page...

Atom-1	Atom-2	Interatomic distance (Å)	Clash overlap (Å)
1:A:812:ARG:HB2	1:A:871:ASN:HB2	1.69	0.75
1:A:872:ASP:OD2	4:A:1201:HOH:O	2.05	0.74
1:A:16:MET:HE2	1:A:155:VAL:H	1.53	0.74
1:A:471:GLN:OE1	1:A:472:ILE:HD12	1.90	0.71
1:A:682:ARG:HH22	1:A:734:ALA:HA	1.56	0.70
1:A:471:GLN:HG3	1:A:472:ILE:N	2.07	0.68
1:A:451:ARG:HD2	1:A:451:ARG:O	1.93	0.68
1:A:962:GLU:N	1:A:962:GLU:OE1	2.30	0.64
1:A:866:ILE:HG13	1:A:869:ARG:HE	1.65	0.62
1:A:636:LEU:HD22	1:A:646:GLU:HG3	1.81	0.61
1:A:781:GLU:HG3	1:A:940:LEU:HD22	1.82	0.60
1:A:776:LYS:O	1:A:780:LYS:HG3	2.02	0.60
1:A:633:LYS:NZ	1:A:796:GLY:HA2	2.18	0.59
1:A:122:ASN:O	1:A:126:TRP:HB2	2.01	0.58
1:A:720:LYS:HD2	1:A:744:ALA:HA	1.85	0.58
1:A:866:ILE:HD11	1:A:869:ARG:HH21	1.68	0.58
1:A:126:TRP:N	1:A:126:TRP:HE3	2.01	0.58
1:A:684:ALA:HB1	1:A:688:THR:O	2.04	0.57
1:A:486:GLU:OE1	1:A:489:ILE:N	2.28	0.57
1:A:861:THR:HA	1:A:866:ILE:HG23	1.88	0.55
1:A:912:ASP:N	1:A:912:ASP:OD2	2.40	0.55
1:A:633:LYS:HE3	1:A:668:MET:HE1	1.87	0.55
1:A:323:GLU:HG2	1:A:387:ASN:HA	1.89	0.55
1:A:618:ALA:HB2	1:A:628:LEU:HD12	1.88	0.54
1:A:677:VAL:O	1:A:681:VAL:HG23	2.07	0.54
1:A:756:LYS:HD3	1:A:759:GLU:OE2	2.08	0.54
1:A:90:ASN:OD1	1:A:91:GLN:N	2.41	0.54
1:A:682:ARG:HH22	1:A:734:ALA:CA	2.21	0.53
1:A:11:PHE:O	1:A:15:ILE:HG12	2.07	0.53
1:A:126:TRP:CE3	1:A:126:TRP:N	2.77	0.53
1:A:323:GLU:HG2	1:A:387:ASN:HB3	1.91	0.53
1:A:72:SER:HB3	1:A:76:ILE:HD11	1.90	0.52
1:A:812:ARG:NH2	4:A:1201:HOH:O	2.43	0.51
1:A:818:TYR:CD2	1:A:828:PRO:HB3	2.45	0.51
1:A:451:ARG:NH1	4:A:1202:HOH:O	2.20	0.51
1:A:811:LEU:HD13	1:A:874:ILE:HG22	1.91	0.50
1:A:318:TRP:CZ2	1:A:431:THR:HG21	2.47	0.50
1:A:805:VAL:HG13	1:A:877:ARG:HH11	1.77	0.49
1:A:724:ILE:H	1:A:724:ILE:HD12	1.77	0.49
1:A:471:GLN:CG	1:A:472:ILE:N	2.76	0.49
1:A:824:PHE:O	1:A:825:LYS:HB2	2.13	0.48

Continued on next page...

CONFIDENTIAL VALIDATION REPORT

Continued from previous page...

Atom-1	Atom-2	Interatomic distance (Å)	Clash overlap (Å)
1:A:793:LEU:HD23	1:A:795:THR:HG22	1.94	0.47
1:A:810:THR:HG23	1:A:813:LYS:H	1.79	0.47
1:A:658:LYS:HE2	1:A:661:ARG:NH2	2.17	0.47
1:A:125:LYS:HB3	1:A:126:TRP:CE3	2.50	0.47
1:A:471:GLN:HG3	1:A:472:ILE:H	1.77	0.47
1:A:481:LYS:HB3	1:A:482:LEU:HD12	1.96	0.46
1:A:694:GLN:OE1	1:A:724:ILE:HG22	2.16	0.46
1:A:991:ARG:NH1	1:A:994:GLU:OE2	2.48	0.46
1:A:323:GLU:HG2	1:A:387:ASN:CB	2.45	0.45
1:A:451:ARG:C	1:A:451:ARG:HD2	2.36	0.45
1:A:810:THR:HG22	1:A:813:LYS:HE3	1.97	0.45
1:A:864:LEU:HB3	1:A:890:PHE:HE1	1.82	0.45
1:A:953:SER:HA	1:A:956:ILE:HG13	1.99	0.45
1:A:493:LEU:HD23	1:A:494:LEU:HD12	1.98	0.45
1:A:827:LYS:N	1:A:828:PRO:CD	2.80	0.45
1:A:16:MET:O	1:A:20:THR:HG23	2.17	0.44
1:A:668:MET:O	1:A:671:VAL:HG22	2.17	0.44
1:A:41:GLN:O	1:A:44:MET:HG2	2.18	0.44
1:A:851:ILE:HG12	1:A:917:ILE:HG23	1.99	0.44
1:A:309:TYR:CZ	1:A:358:LYS:HE3	2.52	0.43
1:A:21:LYS:HB3	1:A:21:LYS:HE2	1.52	0.43
1:A:766:LEU:HD13	1:A:955:MET:HG3	1.99	0.43
1:A:777:ILE:HA	1:A:780:LYS:HE3	2.01	0.43
1:A:805:VAL:HB	2:A:1102:EDO:H11	2.01	0.43
1:A:126:TRP:HH2	1:A:788:VAL:HG22	1.83	0.43
1:A:517:ARG:HD2	1:A:548:LEU:HD22	2.00	0.43
1:A:824:PHE:HD2	1:A:827:LYS:HB2	1.84	0.43
1:A:732:SER:OG	1:A:733:ASN:N	2.51	0.42
1:A:861:THR:HG23	1:A:866:ILE:O	2.20	0.42
1:A:323:GLU:HG2	1:A:387:ASN:CA	2.49	0.42
1:A:915:TYR:HE2	1:A:921:GLU:HG2	1.85	0.42
1:A:869:ARG:HD3	1:A:874:ILE:HD11	2.01	0.42
1:A:778:TRP:CE2	1:A:937:ALA:HB1	2.54	0.42
1:A:8:VAL:HG13	1:A:157:LEU:HD12	2.02	0.42
1:A:147:THR:HB	1:A:149:GLU:OE1	2.20	0.41
1:A:869:ARG:NH1	1:A:873:ASN:HB3	2.35	0.41
1:A:991:ARG:HA	1:A:991:ARG:HD2	1.75	0.41
1:A:827:LYS:N	1:A:828:PRO:HD2	2.36	0.41
1:A:566:ALA:HB2	1:A:602:TYR:CZ	2.55	0.41
1:A:870:HIS:HD2	1:A:872:ASP:H	1.69	0.41
1:A:41:GLN:HB3	1:A:44:MET:CE	2.51	0.41

Continued on next page...

CONFIDENTIAL VALIDATION REPORT

Continued from previous page...

Atom-1	Atom-2	Interatomic distance (Å)	Clash overlap (Å)
1:A:810:THR:CG2	1:A:813:LYS:HB2	2.51	0.41
1:A:552:TRP:HA	1:A:553:PRO:HD3	1.95	0.40
1:A:940:LEU:HA	1:A:940:LEU:HD23	1.89	0.40
1:A:380:LEU:HB2	1:A:409:VAL:HG22	2.02	0.40
1:A:135:LEU:HD12	1:A:139:ALA:HB3	2.04	0.40

There are no symmetry-related clashes.

5.3 Torsion angles (i)

5.3.1 Protein backbone (i)

In the following table, the Percentiles column shows the percent Ramachandran outliers of the chain as a percentile score with respect to all X-ray entries followed by that with respect to entries of similar resolution.

The Analysed column shows the number of residues for which the backbone conformation was analysed, and the total number of residues.

Mol	Chain	Analysed	Favoured	Allowed	Outliers	Percentiles	
1	A	810/910 (89%)	784 (97%)	25 (3%)	1 (0%)	51	67

All (1) Ramachandran outliers are listed below:

Mol	Chain	Res	Type
1	A	827	LYS

5.3.2 Protein sidechains (i)

In the following table, the Percentiles column shows the percent sidechain outliers of the chain as a percentile score with respect to all X-ray entries followed by that with respect to entries of similar resolution.

The Analysed column shows the number of residues for which the sidechain conformation was analysed, and the total number of residues.

Mol	Chain	Analysed	Rotameric	Outliers	Percentiles	
1	A	732/800 (92%)	731 (100%)	1 (0%)	93	98

All (1) residues with a non-rotameric sidechain are listed below:

CONFIDENTIAL VALIDATION REPORT

Mol	Chain	Res	Type
1	A	797	ARG

Sometimes sidechains can be flipped to improve hydrogen bonding and reduce clashes. All (5) such sidechains are listed below:

Mol	Chain	Res	Type
1	A	571	GLN
1	A	672	GLN
1	A	683	GLN
1	A	870	HIS
1	A	873	ASN

5.3.3 RNA (i)

There are no RNA molecules in this entry.

5.4 Non-standard residues in protein, DNA, RNA chains (i)

There are no non-standard protein/DNA/RNA residues in this entry.

5.5 Carbohydrates (i)

There are no monosaccharides in this entry.

5.6 Ligand geometry (i)

3 ligands are modelled in this entry.

In the following table, the Counts columns list the number of bonds (or angles) for which Mogul statistics could be retrieved, the number of bonds (or angles) that are observed in the model and the number of bonds (or angles) that are defined in the Chemical Component Dictionary. The Link column lists molecule types, if any, to which the group is linked. The Z score for a bond length (or angle) is the number of standard deviations the observed value is removed from the expected value. A bond length (or angle) with $|Z| > 2$ is considered an outlier worth inspection. RMSZ is the root-mean-square of all Z scores of the bond lengths (or angles).

Mol	Type	Chain	Res	Link	Bond lengths			Bond angles		
					Counts	RMSZ	$\#\|Z\| > 2$	Counts	RMSZ	$\#\|Z\| > 2$
2	EDO	A	1102	-	3,3,3	0.44	0	2,2,2	0.29	0
2	EDO	A	1101	-	3,3,3	0.43	0	2,2,2	0.37	0
3	SO4	A	1103	-	4,4,4	0.13	0	6,6,6	0.05	0

CONFIDENTIAL VALIDATION REPORT

In the following table, the Chirals column lists the number of chiral outliers, the number of chiral centers analysed, the number of these observed in the model and the number defined in the Chemical Component Dictionary. Similar counts are reported in the Torsion and Rings columns. '-' means no outliers of that kind were identified.

Mol	Type	Chain	Res	Link	Chirals	Torsions	Rings
2	EDO	A	1102	-	-	0/1/1/1	-
2	EDO	A	1101	-	-	0/1/1/1	-

There are no bond length outliers.

There are no bond angle outliers.

There are no chirality outliers.

There are no torsion outliers.

There are no ring outliers.

1 monomer is involved in 1 short contact:

Mol	Chain	Res	Type	Clashes	Symm-Clashes
2	A	1102	EDO	1	0

5.7 Other polymers (i)

There are no such residues in this entry.

5.8 Polymer linkage issues (i)

There are no chain breaks in this entry.

CONFIDENTIAL VALIDATION REPORT

6 Fit of model and data (i)

6.1 Protein, DNA and RNA chains (i)

In the following table, the column labelled '#RSRZ> 2' contains the number (and percentage) of RSRZ outliers, followed by percent RSRZ outliers for the chain as percentile scores relative to all X-ray entries and entries of similar resolution. The OWAB column contains the minimum, median, 95th percentile and maximum values of the occupancy-weighted average B-factor per residue. The column labelled 'Q< 0.9' lists the number of (and percentage) of residues with an average occupancy less than 0.9.

Mol	Chain	Analysed	<RSRZ>	#RSRZ>2	OWAB(Å^2)	Q<0.9
1	A	822/910 (90%)	0.28	38 (4%) 32 30	39, 66, 112, 148	0

All (38) RSRZ outliers are listed below:

Mol	Chain	Res	Type	RSRZ
1	A	402	GLY	5.2
1	A	476	ILE	4.8
1	A	44	MET	4.2
1	A	733	ASN	3.5
1	A	905	ALA	3.5
1	A	90	ASN	3.3
1	A	682	ARG	3.3
1	A	484	THR	3.1
1	A	693	LEU	3.0
1	A	734	ALA	3.0
1	A	91	GLN	3.0
1	A	477	ILE	2.9
1	A	323	GLU	2.8
1	A	729	PHE	2.7
1	A	979	GLN	2.7
1	A	684	ALA	2.7
1	A	314	ILE	2.6
1	A	867	CYS	2.6
1	A	522	LYS	2.5
1	A	89	LEU	2.5
1	A	825	LYS	2.5
1	A	43	ASN	2.5
1	A	387	ASN	2.5
1	A	152	GLU	2.4
1	A	494	LEU	2.4
1	A	688	THR	2.3
1	A	824	PHE	2.2

Continued on next page...

CONFIDENTIAL VALIDATION REPORT

Continued from previous page...

Mol	Chain	Res	Type	RSRZ
1	A	318	TRP	2.2
1	A	316	SER	2.2
1	A	594	PRO	2.2
1	A	504	GLY	2.1
1	A	431	THR	2.1
1	A	991	ARG	2.1
1	A	319	VAL	2.1
1	A	126	TRP	2.1
1	A	730	PHE	2.1
1	A	505	LEU	2.0
1	A	157	LEU	2.0

6.2 Non-standard residues in protein, DNA, RNA chains (i)

There are no non-standard protein/DNA/RNA residues in this entry.

6.3 Carbohydrates (i)

There are no monosaccharides in this entry.

6.4 Ligands (i)

In the following table, the Atoms column lists the number of modelled atoms in the group and the number defined in the chemical component dictionary. The B-factors column lists the minimum, median, 95th percentile and maximum values of B factors of atoms in the group. The column labelled 'Q< 0.9' lists the number of atoms with occupancy less than 0.9.

Mol	Type	Chain	Res	Atoms	RSCC	RSR	B-factors($Å^2$)	Q<0.9
2	EDO	A	1101	4/4	0.83	0.37	68,70,77,82	0
3	SO4	A	1103	5/5	0.86	0.32	117,118,126,128	0
2	EDO	A	1102	4/4	0.88	0.46	95,96,105,111	0

6.5 Other polymers (i)

There are no such residues in this entry.

CONFIDENTIAL VALIDATION REPORT

Full wwPDB X-ray Structure Validation Report (i)

Jan 14, 2021 – 05:36 PM GMT

PDB ID	:	7BI6
Title	:	PI3KC2a core in complex with ATP
Deposited on	:	2021-01-12
Resolution	:	2.75 Å(reported)

This is a Full wwPDB X-ray Structure Validation Report.

This report is produced by the wwPDB biocuration pipeline after annotation of the structure.

We welcome your comments at *validation@mail.wwpdb.org*
A user guide is available at
https://www.wwpdb.org/validation/2017/XrayValidationReportHelp
with specific help available everywhere you see the (i) symbol.

CONFIDENTIAL VALIDATION REPORT

The following versions of software and data (see references (i)) were used in the production of this report:

MolProbity	:	4.02b-467
Mogul	:	1.8.5 (274361), CSD as541be (2020)
Xtriage (Phenix)	:	1.13
EDS	:	2.16
buster-report	:	1.1.7 (2018)
Percentile statistics	:	20191225.v01 (using entries in the PDB archive December 25th 2019)
Refmac	:	5.8.0158
CCP4	:	7.0.044 (Gargrove)
Ideal geometry (proteins)	:	Engh & Huber (2001)
Ideal geometry (DNA, RNA)	:	Parkinson et al. (1996)
Validation Pipeline (wwPDB-VP)	:	2.16

1 Overall quality at a glance (i)

The following experimental techniques were used to determine the structure:
X-RAY DIFFRACTION

The reported resolution of this entry is 2.75 Å.

Percentile scores (ranging between 0-100) for global validation metrics of the entry are shown in the following graphic. The table shows the number of entries on which the scores are based.

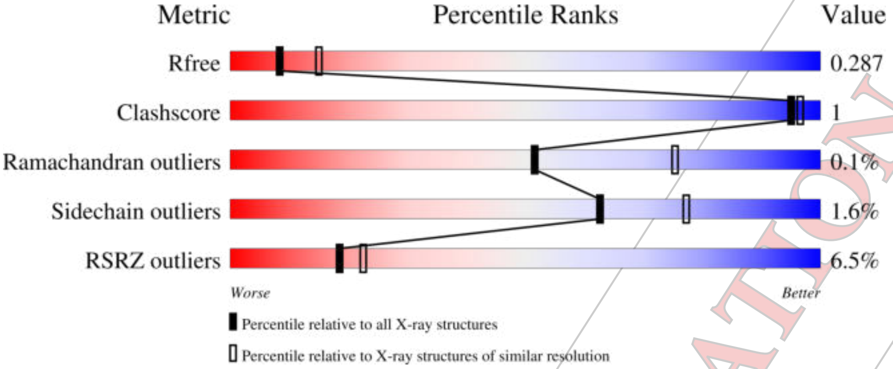

Metric	Whole archive (#Entries)	Similar resolution (#Entries, resolution range(Å))
R_{free}	130704	1235 (2.78-2.74)
Clashscore	141614	1277 (2.78-2.74)
Ramachandran outliers	138981	1257 (2.78-2.74)
Sidechain outliers	138945	1257 (2.78-2.74)
RSRZ outliers	127900	1207 (2.78-2.74)

The table below summarises the geometric issues observed across the polymeric chains and their fit to the electron density. The red, orange, yellow and green segments of the lower bar indicate the fraction of residues that contain outliers for >=3, 2, 1 and 0 types of geometric quality criteria respectively. A grey segment represents the fraction of residues that are not modelled. The numeric value for each fraction is indicated below the corresponding segment, with a dot representing fractions <=5% The upper red bar (where present) indicates the fraction of residues that have poor fit to the electron density. The numeric value is given above the bar.

Mol	Chain	Length	Quality of chain
1	A	910	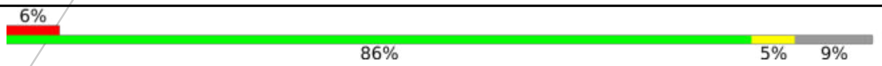

The following table lists non-polymeric compounds, carbohydrate monomers and non-standard residues in protein, DNA, RNA chains that are outliers for geometric or electron-density-fit criteria:

Mol	Type	Chain	Res	Chirality	Geometry	Clashes	Electron density
4	EDO	A	1105	-	-	-	X

CONFIDENTIAL VALIDATION REPORT

2 Entry composition (i)

There are 6 unique types of molecules in this entry. The entry contains 6747 atoms, of which 0 are hydrogens and 0 are deuteriums.

In the tables below, the ZeroOcc column contains the number of atoms modelled with zero occupancy, the AltConf column contains the number of residues with at least one atom in alternate conformation and the Trace column contains the number of residues modelled with at most 2 atoms.

- Molecule 1 is a protein called Phosphatidylinositol 4-phosphate 3-kinase C2 domain-containing subunit alpha,Phosphatidylinositol 4-phosphate 3-kinase C2 domain-containing subunit alpha.

Mol	Chain	Residues	Atoms					ZeroOcc	AltConf	Trace
			Total	C	N	O	S			
1	A	829	6611	4244	1109	1217	41	0	5	0

There are 23 discrepancies between the modelled and reference sequences:

Chain	Residue	Modelled	Actual	Comment	Reference
A	1	GLY	-	expression tag	UNP Q61194
A	2	ALA	-	expression tag	UNP Q61194
A	159	GLY	-	linker	UNP Q61194
A	268	SER	-	linker	UNP Q61194
A	269	GLY	-	linker	UNP Q61194
A	270	SER	-	linker	UNP Q61194
A	271	VAL	-	linker	UNP Q61194
A	272	MET	-	linker	UNP Q61194
A	273	THR	-	linker	UNP Q61194
A	274	ARG	-	linker	UNP Q61194
A	275	HIS	-	linker	UNP Q61194
A	276	SER	-	linker	UNP Q61194
A	277	ALA	-	linker	UNP Q61194
A	278	GLY	-	linker	UNP Q61194
A	279	ALA	-	linker	UNP Q61194
A	280	GLY	-	linker	UNP Q61194
A	281	SER	-	linker	UNP Q61194
A	282	GLY	-	linker	UNP Q61194
A	283	ALA	-	linker	UNP Q61194
A	286	GLY	ALA	conflict	UNP Q61194
A	353	ALA	PHE	engineered mutation	UNP Q61194
A	354	ALA	PHE	engineered mutation	UNP Q61194
A	427	ALA	LEU	engineered mutation	UNP Q61194

- Molecule 2 is ADENOSINE-5'-TRIPHOSPHATE (three-letter code: ATP) (formula:

CONFIDENTIAL VALIDATION REPORT

$C_{10}H_{16}N_5O_{13}P_3$).

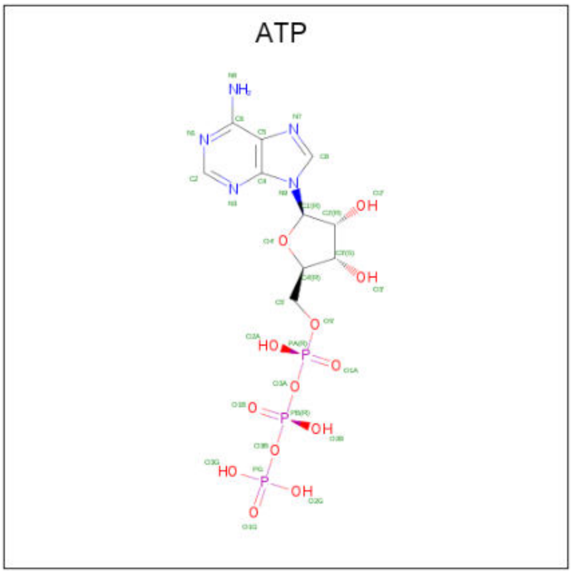

Mol	Chain	Residues	Atoms					ZeroOcc	AltConf
			Total	C	N	O	P		
2	A	1	31	10	5	13	3	0	0

- Molecule 3 is SULFATE ION (three-letter code: SO4) (formula: O_4S).

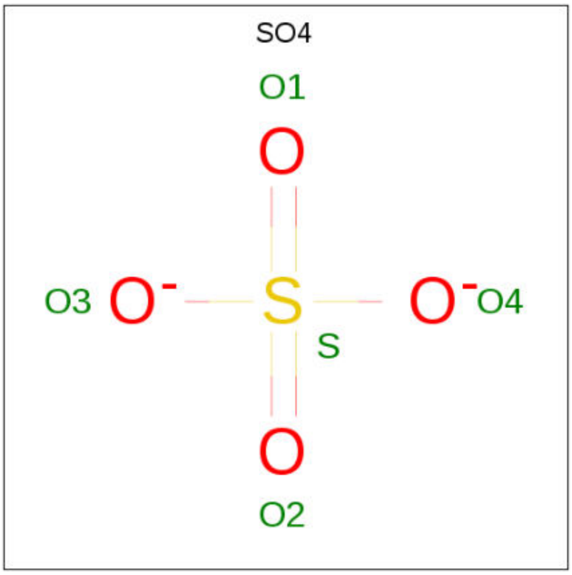

Mol	Chain	Residues	Atoms			ZeroOcc	AltConf
			Total	O	S		
3	A	1	5	4	1	0	0
3	A	1	5	4	1	0	0

CONFIDENTIAL VALIDATION REPORT

- Molecule 4 is 1,2-ETHANEDIOL (three-letter code: EDO) (formula: $C_2H_6O_2$).

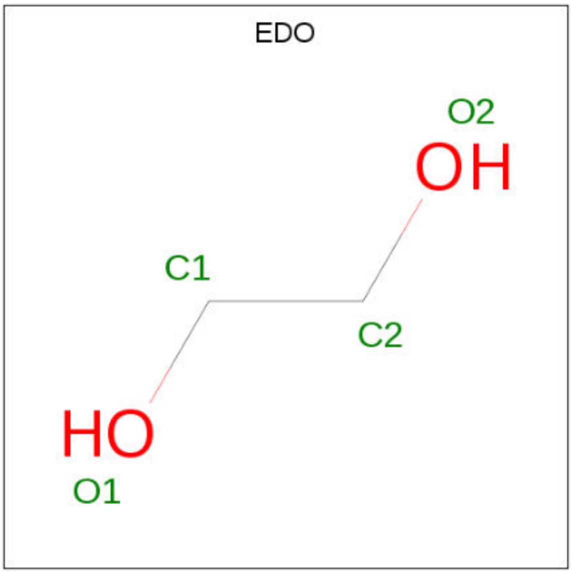

Mol	Chain	Residues	Atoms	ZeroOcc	AltConf
4	A	1	Total C O<br>4 2 2	0	0
4	A	1	Total C O<br>4 2 2	0	0

- Molecule 5 is MAGNESIUM ION (three-letter code: MG) (formula: Mg).

Mol	Chain	Residues	Atoms	ZeroOcc	AltConf
5	A	1	Total Mg<br>1 1	0	0

- Molecule 6 is water.

Mol	Chain	Residues	Atoms	ZeroOcc	AltConf
6	A	86	Total O<br>86 86	0	0

CONFIDENTIAL VALIDATION REPORT

3 Residue-property plots (i)

These plots are drawn for all protein, RNA, DNA and oligosaccharide chains in the entry. The first graphic for a chain summarises the proportions of the various outlier classes displayed in the second graphic. The second graphic shows the sequence view annotated by issues in geometry and electron density. Residues are color-coded according to the number of geometric quality criteria for which they contain at least one outlier: green = 0, yellow = 1, orange = 2 and red = 3 or more. A red dot above a residue indicates a poor fit to the electron density (RSRZ > 2). Stretches of 2 or more consecutive residues without any outlier are shown as a green connector. Residues present in the sample, but not in the model, are shown in grey.

• Molecule 1: Phosphatidylinositol 4-phosphate 3-kinase C2 domain-containing subunit alpha,Phosphatidylinositol 4-phosphate 3-kinase C2 domain-containing subunit alpha

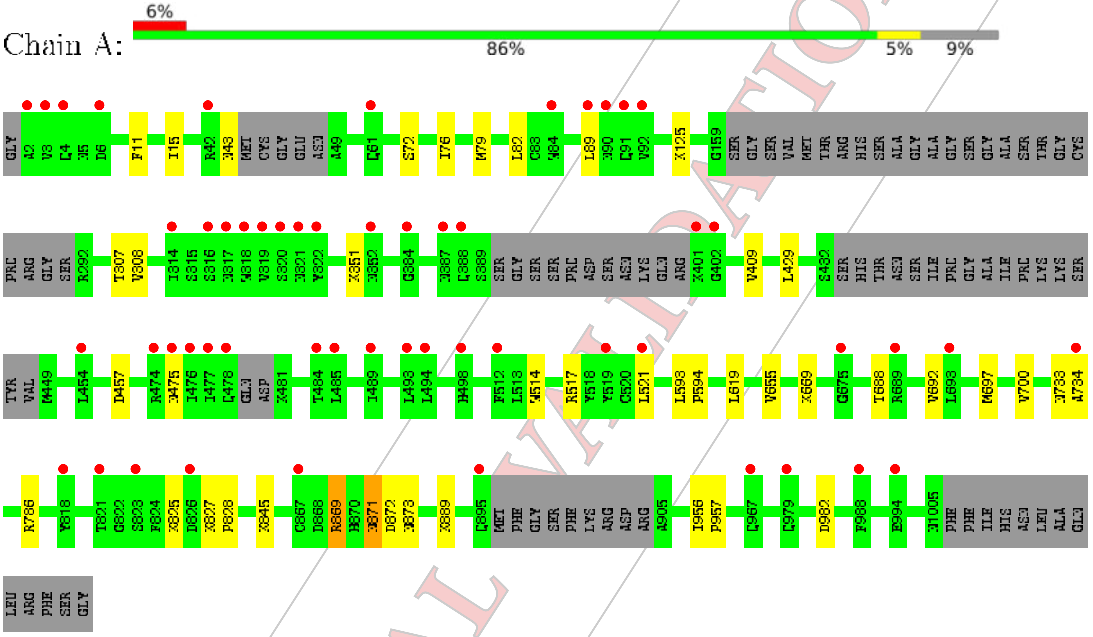

CONFIDENTIAL VALIDATION REPORT

4 Data and refinement statistics (i)

Property	Value	Source
Space group	P 21 21 21	Depositor
Cell constants<br>a, b, c, α, β, γ	56.12Å 133.37Å 152.71Å<br>90.00° 90.00° 90.00°	Depositor
Resolution (Å)	47.56 – 2.75<br>47.56 – 2.75	Depositor<br>EDS
% Data completeness<br>(in resolution range)	98.8 (47.56-2.75)<br>98.9 (47.56-2.75)	Depositor<br>EDS
R_{merge}	(Not available)	Depositor
R_{sym}	(Not available)	Depositor
$< I/\sigma(I) >$ [1]	1.06 (at 2.73Å)	Xtriage
Refinement program	REFMAC 5.8.0258	Depositor
R, R_{free}	0.244 , 0.289<br>0.245 , 0.287	Depositor<br>DCC
R_{free} test set	1521 reflections (5.00%)	wwPDB-VP
Wilson B-factor (Å^2)	68.7	Xtriage
Anisotropy	0.429	Xtriage
Bulk solvent k_{sol}(e/Å^3), B_{sol}(Å^2)	0.30 , 36.0	EDS
L-test for twinning[2]	$< \|L\| >$ = 0.49, $< L^2 >$ = 0.33	Xtriage
Estimated twinning fraction	No twinning to report.	Xtriage
F_o,F_c correlation	0.93	EDS
Total number of atoms	6747	wwPDB-VP
Average B, all atoms (Å^2)	79.0	wwPDB-VP

Xtriage's analysis on translational NCS is as follows: *The largest off-origin peak in the Patterson function is 4.55% of the height of the origin peak. No significant pseudotranslation is detected.*

[1] Intensities estimated from amplitudes.
[2] Theoretical values of $< |L| >$, $< L^2 >$ for acentric reflections are 0.5, 0.333 respectively for untwinned datasets, and 0.375, 0.2 for perfectly twinned datasets.

CONFIDENTIAL VALIDATION REPORT

5 Model quality (i)

5.1 Standard geometry (i)

Bond lengths and bond angles in the following residue types are not validated in this section: MG, SO4, ATP, EDO

The Z score for a bond length (or angle) is the number of standard deviations the observed value is removed from the expected value. A bond length (or angle) with $|Z| > 5$ is considered an outlier worth inspection. RMSZ is the root-mean-square of all Z scores of the bond lengths (or angles).

Mol	Chain	Bond lengths		Bond angles	
		RMSZ	$\#\|Z\| > 5$	RMSZ	$\#\|Z\| > 5$
1	A	0.66	0/6760	0.70	0/9149

There are no bond length outliers.

There are no bond angle outliers.

There are no chirality outliers.

There are no planarity outliers.

5.2 Too-close contacts (i)

In the following table, the Non-H and H(model) columns list the number of non-hydrogen atoms and hydrogen atoms in the chain respectively. The H(added) column lists the number of hydrogen atoms added and optimized by MolProbity. The Clashes column lists the number of clashes within the asymmetric unit, whereas Symm-Clashes lists symmetry-related clashes.

Mol	Chain	Non-H	H(model)	H(added)	Clashes	Symm-Clashes
1	A	6611	0	6672	16	0
2	A	31	0	12	0	0
3	A	10	0	0	0	0
4	A	8	0	12	0	0
5	A	1	0	0	0	0
6	A	86	0	0	0	0
All	All	6747	0	6696	16	0

The all-atom clashscore is defined as the number of clashes found per 1000 atoms (including hydrogen atoms). The all-atom clashscore for this structure is 1.

All (16) close contacts within the same asymmetric unit are listed below, sorted by their clash magnitude.

CONFIDENTIAL VALIDATION REPORT

Atom-1	Atom-2	Interatomic distance (Å)	Clash overlap (Å)
1:A:514:TRP:O	1:A:517:ARG:NE	2.35	0.59
1:A:72:SER:HB2	1:A:76:ILE:HD11	1.87	0.57
1:A:871:ASN:HD22	1:A:872:ASP:N	2.08	0.52
1:A:956:ILE:N	1:A:957:PRO:HD2	2.27	0.49
1:A:409:VAL:HG23	1:A:429:LEU:HB3	1.94	0.49
1:A:125:LYS:HD3	1:A:786:ARG:HA	1.95	0.48
1:A:827:LYS:N	1:A:828:PRO:CD	2.77	0.47
1:A:307:THR:HG23	1:A:457:ASP:HB2	1.98	0.45
1:A:11:PHE:O	1:A:15:ILE:HG12	2.18	0.44
1:A:593:LEU:N	1:A:594:PRO:CD	2.81	0.44
1:A:79:MET:HA	1:A:82:LEU:HB2	2.01	0.43
1:A:688:THR:HG22	1:A:692:VAL:HG23	2.01	0.43
1:A:697:MET:HE1	1:A:700:VAL:HG21	2.01	0.42
1:A:619:LEU:HD21	1:A:655:VAL:HG22	2.01	0.42
1:A:733:ASN:O	1:A:734:ALA:HB3	2.19	0.41
1:A:869:ARG:NH1	1:A:873:ASN:HB3	2.35	0.40

There are no symmetry-related clashes.

5.3 Torsion angles (i)

5.3.1 Protein backbone (i)

In the following table, the Percentiles column shows the percent Ramachandran outliers of the chain as a percentile score with respect to all X-ray entries followed by that with respect to entries of similar resolution.

The Analysed column shows the number of residues for which the backbone conformation was analysed, and the total number of residues.

Mol	Chain	Analysed	Favoured	Allowed	Outliers	Percentiles	
1	A	820/910 (90%)	775 (94%)	44 (5%)	1 (0%)	51	75

All (1) Ramachandran outliers are listed below:

Mol	Chain	Res	Type
1	A	308	VAL

CONFIDENTIAL VALIDATION REPORT

5.3.2 Protein sidechains (i)

In the following table, the Percentiles column shows the percent sidechain outliers of the chain as a percentile score with respect to all X-ray entries followed by that with respect to entries of similar resolution.

The Analysed column shows the number of residues for which the sidechain conformation was analysed, and the total number of residues.

Mol	Chain	Analysed	Rotameric	Outliers	Percentiles	
1	A	738/800 (92%)	726 (98%)	12 (2%)	62	77

All (12) residues with a non-rotameric sidechain are listed below:

Mol	Chain	Res	Type
1	A	43	ASN
1	A	89	LEU
1	A	351	LYS
1	A	475	ASN
1	A	521	LEU
1	A	669	LYS
1	A	825	LYS
1	A	845	LYS
1	A	869	ARG
1	A	871	ASN
1	A	889	LYS
1	A	982	ASP

Sometimes sidechains can be flipped to improve hydrogen bonding and reduce clashes. All (5) such sidechains are listed below:

Mol	Chain	Res	Type
1	A	43	ASN
1	A	80	GLN
1	A	475	ASN
1	A	667	GLN
1	A	871	ASN

5.3.3 RNA (i)

There are no RNA molecules in this entry.

CONFIDENTIAL VALIDATION REPORT

5.4 Non-standard residues in protein, DNA, RNA chains (i)

There are no non-standard protein/DNA/RNA residues in this entry.

5.5 Carbohydrates (i)

There are no monosaccharides in this entry.

5.6 Ligand geometry (i)

Of 6 ligands modelled in this entry, 1 is monoatomic - leaving 5 for Mogul analysis.

In the following table, the Counts columns list the number of bonds (or angles) for which Mogul statistics could be retrieved, the number of bonds (or angles) that are observed in the model and the number of bonds (or angles) that are defined in the Chemical Component Dictionary. The Link column lists molecule types, if any, to which the group is linked. The Z score for a bond length (or angle) is the number of standard deviations the observed value is removed from the expected value. A bond length (or angle) with $|Z| > 2$ is considered an outlier worth inspection. RMSZ is the root-mean-square of all Z scores of the bond lengths (or angles).

Mol	Type	Chain	Res	Link	Bond lengths			Bond angles		
					Counts	RMSZ	$\#\|Z\| > 2$	Counts	RMSZ	$\#\|Z\| > 2$
3	SO4	A	1103	-	4,4,4	0.39	0	6,6,6	0.05	0
2	ATP	A	1101	5	26,33,33	0.65	0	31,52,52	0.74	1 (3%)
4	EDO	A	1105	-	3,3,3	0.05	0	2,2,2	0.25	0
3	SO4	A	1102	-	4,4,4	0.39	0	6,6,6	0.05	0
4	EDO	A	1104	-	3,3,3	0.06	0	2,2,2	0.19	0

In the following table, the Chirals column lists the number of chiral outliers, the number of chiral centers analysed, the number of these observed in the model and the number defined in the Chemical Component Dictionary. Similar counts are reported in the Torsion and Rings columns. '-' means no outliers of that kind were identified.

Mol	Type	Chain	Res	Link	Chirals	Torsions	Rings
2	ATP	A	1101	5	-	0/18/38/38	0/3/3/3
4	EDO	A	1105	-	-	1/1/1/1	-
4	EDO	A	1104	-	-	0/1/1/1	-

There are no bond length outliers.

All (1) bond angle outliers are listed below:

Mol	Chain	Res	Type	Atoms	Z	Observed(°)	Ideal(°)
2	A	1101	ATP	C5-C6-N6	2.29	123.83	120.35

CONFIDENTIAL VALIDATION REPORT

There are no chirality outliers.

All (1) torsion outliers are listed below:

Mol	Chain	Res	Type	Atoms
4	A	1105	EDO	O1-C1-C2-O2

There are no ring outliers.

No monomer is involved in short contacts.

The following is a two-dimensional graphical depiction of Mogul quality analysis of bond lengths, bond angles, torsion angles, and ring geometry for all instances of the Ligand of Interest. In addition, ligands with molecular weight > 250 and outliers as shown on the validation Tables will also be included. For torsion angles, if less then 5% of the Mogul distribution of torsion angles is within 10 degrees of the torsion angle in question, then that torsion angle is considered an outlier. Any bond that is central to one or more torsion angles identified as an outlier by Mogul will be highlighted in the graph. For rings, the root-mean-square deviation (RMSD) between the ring in question and similar rings identified by Mogul is calculated over all ring torsion angles. If the average RMSD is greater than 60 degrees and the minimal RMSD between the ring in question and any Mogul-identified rings is also greater than 60 degrees, then that ring is considered an outlier. The outliers are highlighted in purple. The color gray indicates Mogul did not find sufficient equivalents in the CSD to analyse the geometry.

Ligand ATP A 1101

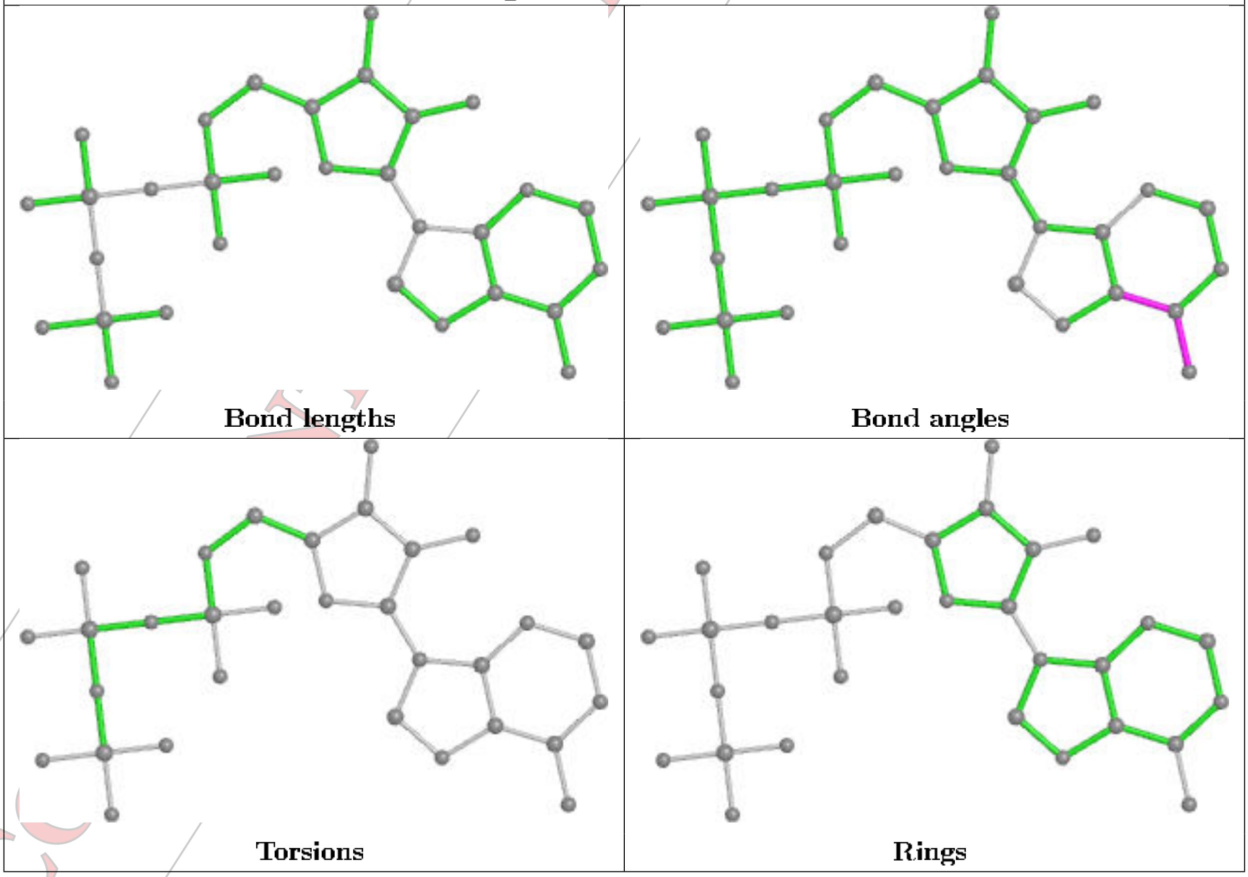

VALIDATION REPORT

5.7 Other polymers (i)

There are no such residues in this entry.

5.8 Polymer linkage issues (i)

There are no chain breaks in this entry.

CONFIDENTIAL VALIDATION REPORT

6 Fit of model and data (i)

6.1 Protein, DNA and RNA chains (i)

In the following table, the column labelled '#RSRZ> 2' contains the number (and percentage) of RSRZ outliers, followed by percent RSRZ outliers for the chain as percentile scores relative to all X-ray entries and entries of similar resolution. The OWAB column contains the minimum, median, 95th percentile and maximum values of the occupancy-weighted average B-factor per residue. The column labelled 'Q< 0.9' lists the number of (and percentage) of residues with an average occupancy less than 0.9.

Mol	Chain	Analysed	<RSRZ>	#RSRZ>2	OWAB(Å^2)	Q<0.9
1	A	829/910 (91%)	0.64	54 (6%) 18 22	50, 76, 115, 152	0

All (54) RSRZ outliers are listed below:

Mol	Chain	Res	Type	RSRZ
1	A	89	LEU	5.3
1	A	867	CYS	4.7
1	A	475	ASN	4.5
1	A	321	ASN	3.8
1	A	320	SER	3.7
1	A	675	GLY	3.5
1	A	734	ALA	3.5
1	A	476	ILE	3.4
1	A	90	ASN	3.4
1	A	818	TYR	3.4
1	A	498	HIS	3.4
1	A	494	LEU	3.2
1	A	693	LEU	3.2
1	A	352	ASN	3.2
1	A	521	LEU	3.2
1	A	319	VAL	3.1
1	A	318	TRP	3.1
1	A	489	ILE	3.1
1	A	92	VAL	3.0
1	A	689	ARG	2.9
1	A	979	GLN	2.9
1	A	988	PHE	2.9
1	A	387	ASN	2.9
1	A	478	GLN	2.7
1	A	388	GLN	2.7
1	A	6	ASP	2.6
1	A	3	VAL	2.6

Continued on next page...

CONFIDENTIAL VALIDATION REPORT

Continued from previous page...

Mol	Chain	Res	Type	RSRZ
1	A	91	GLN	2.5
1	A	314	ILE	2.5
1	A	401	LYS	2.5
1	A	474	ARG	2.5
1	A	967	GLN	2.5
1	A	477	ILE	2.5
1	A	2	ALA	2.5
1	A	322	TYR	2.5
1	A	402	GLY	2.4
1	A	61	GLN	2.4
1	A	384	GLY	2.4
1	A	821	THR	2.3
1	A	823	SER	2.3
1	A	84	TRP	2.3
1	A	994	GLU	2.3
1	A	493	LEU	2.2
1	A	42	ARG	2.2
1	A	4	GLN	2.2
1	A	485	LEU	2.2
1	A	317	ASN	2.2
1	A	512	PHE	2.2
1	A	826	ASP	2.1
1	A	484	THR	2.1
1	A	895	GLN	2.0
1	A	519	TYR	2.0
1	A	316	SER	2.0
1	A	454	LEU	2.0

6.2 Non-standard residues in protein, DNA, RNA chains (i)

There are no non-standard protein/DNA/RNA residues in this entry.

6.3 Carbohydrates (i)

There are no monosaccharides in this entry.

6.4 Ligands (i)

In the following table, the Atoms column lists the number of modelled atoms in the group and the number defined in the chemical component dictionary. The B-factors column lists the minimum,

CONFIDENTIAL VALIDATION REPORT

median, 95th percentile and maximum values of B factors of atoms in the group. The column labelled 'Q< 0.9' lists the number of atoms with occupancy less than 0.9.

Mol	Type	Chain	Res	Atoms	RSCC	RSR	B-factors($Å^2$)	Q<0.9
4	EDO	A	1105	4/4	0.80	0.47	87,87,88,88	0
5	MG	A	1106	1/1	0.84	0.17	70,70,70,70	0
3	SO4	A	1103	5/5	0.85	0.39	139,140,140,140	0
3	SO4	A	1102	5/5	0.85	0.23	138,139,139,139	0
2	ATP	A	1101	31/31	0.91	0.16	73,80,109,109	0
4	EDO	A	1104	4/4	0.91	0.17	79,79,80,80	0

The following is a graphical depiction of the model fit to experimental electron density of all instances of the Ligand of Interest. In addition, ligands with molecular weight > 250 and outliers as shown on the geometry validation Tables will also be included. Each fit is shown from different orientation to approximate a three-dimensional view.

Electron density around ATP A 1101:

$2mF_o$-DF_c (at 0.7 rmsd) in gray
mF_o-DF_c (at 3 rmsd) in purple (negative) and green (positive)

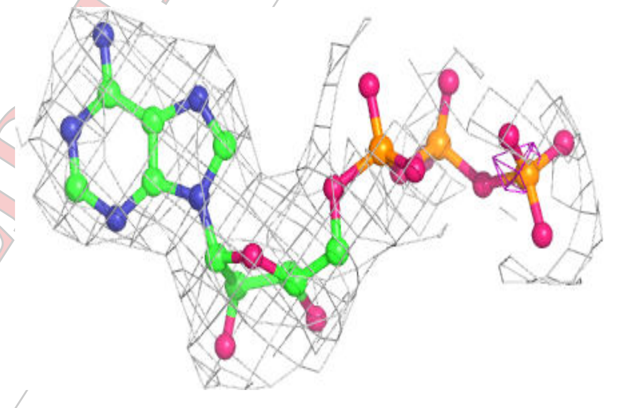

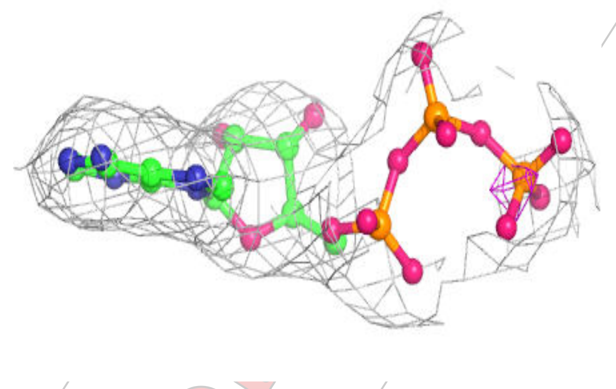

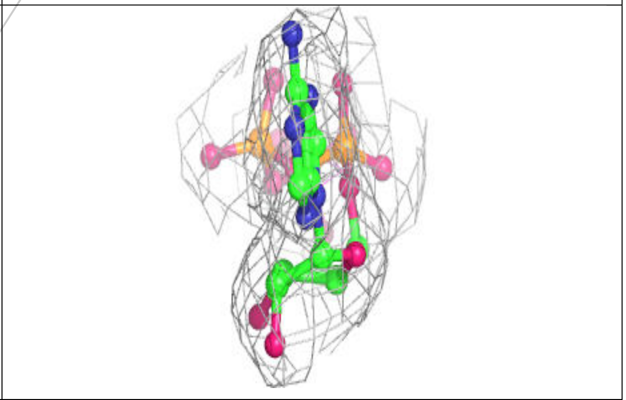

6.5 Other polymers ⓘ

There are no such residues in this entry.

CONFIDENTIAL VALIDATION REPORT

Full wwPDB X-ray Structure Validation Report (i)

Jan 15, 2021 – 06:08 PM GMT

PDB ID	:	7BI9
Title	:	PI3KC2a core in complex with PIK90
Deposited on	:	2021-01-12
Resolution	:	2.65 Å(reported)

This is a Full wwPDB X-ray Structure Validation Report.

This report is produced by the wwPDB biocuration pipeline after annotation of the structure.

We welcome your comments at *validation@mail.wwpdb.org*
A user guide is available at
https://www.wwpdb.org/validation/2017/XrayValidationReportHelp
with specific help available everywhere you see the (i) symbol.

CONFIDENTIAL VALIDATION REPORT

The following versions of software and data (see references (i)) were used in the production of this report:

MolProbity	:	4.02b-467
Mogul	:	1.8.5 (274361), CSD as541be (2020)
Xtriage (Phenix)	:	1.13
EDS	:	2.16
buster-report	:	1.1.7 (2018)
Percentile statistics	:	20191225.v01 (using entries in the PDB archive December 25th 2019)
Refmac	:	5.8.0158
CCP4	:	7.0.044 (Gargrove)
Ideal geometry (proteins)	:	Engh & Huber (2001)
Ideal geometry (DNA, RNA)	:	Parkinson et al. (1996)
Validation Pipeline (wwPDB-VP)	:	2.16

1 Overall quality at a glance (i)

The following experimental techniques were used to determine the structure:
X-RAY DIFFRACTION

The reported resolution of this entry is 2.65 Å.

Percentile scores (ranging between 0-100) for global validation metrics of the entry are shown in the following graphic. The table shows the number of entries on which the scores are based.

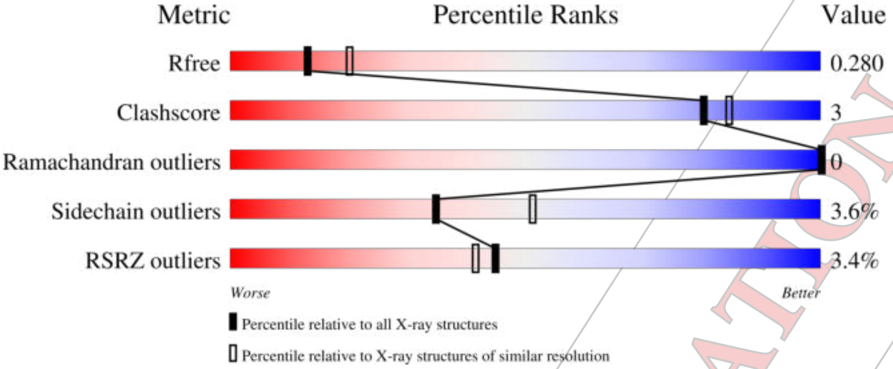

Metric	Whole archive (#Entries)	Similar resolution (#Entries, resolution range(Å))
R_{free}	130704	1332 (2.68-2.64)
Clashscore	141614	1374 (2.68-2.64)
Ramachandran outliers	138981	1349 (2.68-2.64)
Sidechain outliers	138945	1349 (2.68-2.64)
RSRZ outliers	127900	1318 (2.68-2.64)

The table below summarises the geometric issues observed across the polymeric chains and their fit to the electron density. The red, orange, yellow and green segments of the lower bar indicate the fraction of residues that contain outliers for >=3, 2, 1 and 0 types of geometric quality criteria respectively. A grey segment represents the fraction of residues that are not modelled. The numeric value for each fraction is indicated below the corresponding segment, with a dot representing fractions <=5% The upper red bar (where present) indicates the fraction of residues that have poor fit to the electron density. The numeric value is given above the bar.

Mol	Chain	Length	Quality of chain
1	A	910	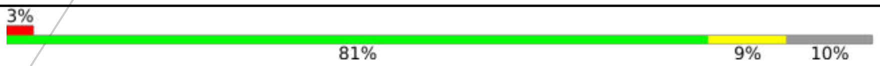

The following table lists non-polymeric compounds, carbohydrate monomers and non-standard residues in protein, DNA, RNA chains that are outliers for geometric or electron-density-fit criteria:

Mol	Type	Chain	Res	Chirality	Geometry	Clashes	Electron density
3	EDO	A	1102	-	-	-	X

Continued on next page...

CONFIDENTIAL VALIDATION REPORT

Continued from previous page...

Mol	Type	Chain	Res	Chirality	Geometry	Clashes	Electron density
3	EDO	A	1106	-	-	-	X

CONFIDENTIAL VALIDATION REPORT

2 Entry composition (i)

There are 4 unique types of molecules in this entry. The entry contains 6642 atoms, of which 0 are hydrogens and 0 are deuteriums.

In the tables below, the ZeroOcc column contains the number of atoms modelled with zero occupancy, the AltConf column contains the number of residues with at least one atom in alternate conformation and the Trace column contains the number of residues modelled with at most 2 atoms.

- Molecule 1 is a protein called Phosphatidylinositol 4-phosphate 3-kinase C2 domain-containing subunit alpha,Phosphatidylinositol 4-phosphate 3-kinase C2 domain-containing subunit alpha.

Mol	Chain	Residues	Atoms					ZeroOcc	AltConf	Trace
			Total	C	N	O	S			
1	A	821	6540	4201	1097	1201	41	0	1	0

There are 23 discrepancies between the modelled and reference sequences:

Chain	Residue	Modelled	Actual	Comment	Reference
A	1	GLY	-	expression tag	UNP Q61194
A	2	ALA	-	expression tag	UNP Q61194
A	267	GLY	-	linker	UNP Q61194
A	268	SER	-	linker	UNP Q61194
A	269	GLY	-	linker	UNP Q61194
A	270	SER	-	linker	UNP Q61194
A	271	VAL	-	linker	UNP Q61194
A	272	MET	-	linker	UNP Q61194
A	273	THR	-	linker	UNP Q61194
A	274	ARG	-	linker	UNP Q61194
A	275	HIS	-	linker	UNP Q61194
A	276	SER	-	linker	UNP Q61194
A	277	ALA	-	linker	UNP Q61194
A	278	GLY	-	linker	UNP Q61194
A	279	ALA	-	linker	UNP Q61194
A	280	GLY	-	linker	UNP Q61194
A	281	SER	-	linker	UNP Q61194
A	282	GLY	-	linker	UNP Q61194
A	283	ALA	-	linker	UNP Q61194
A	286	GLY	ALA	conflict	UNP Q61194
A	353	ALA	PHE	engineered mutation	UNP Q61194
A	354	ALA	PHE	engineered mutation	UNP Q61194
A	427	ALA	LEU	engineered mutation	UNP Q61194

- Molecule 2 is N-(2,3-DIHYDRO-7,8-DIMETHOXYIMIDAZO[1,2-C] QUINAZOLIN-5-YL)

CONFIDENTIAL VALIDATION REPORT

NICOTINAMIDE (three-letter code: 090) (formula: $C_{18}H_{16}N_5O_3$).

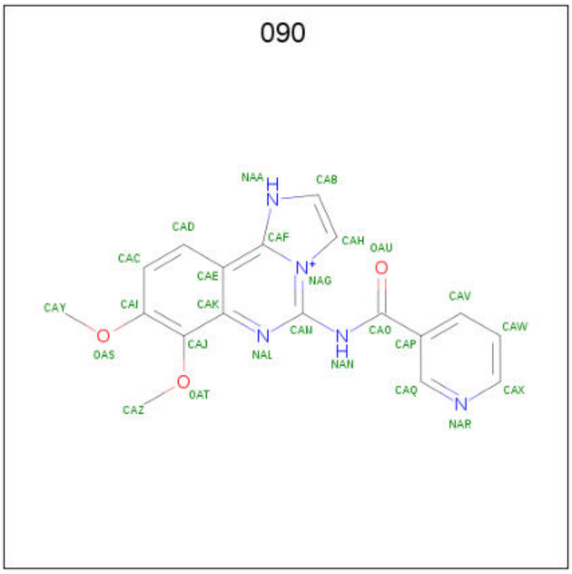

Mol	Chain	Residues	Atoms	ZeroOcc	AltConf
2	A	1	Total C N O<br>26 18 5 3	0	0

- Molecule 3 is 1,2-ETHANEDIOL (three-letter code: EDO) (formula: $C_2H_6O_2$).

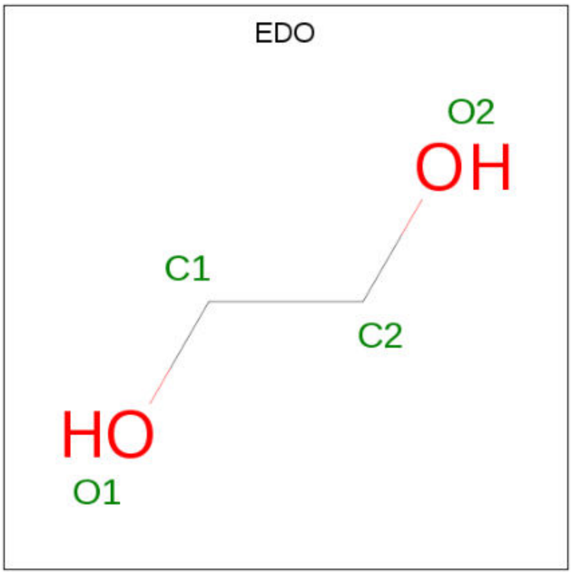

Mol	Chain	Residues	Atoms	ZeroOcc	AltConf
3	A	1	Total C O<br>4 2 2	0	0
3	A	1	Total C O<br>4 2 2	0	0

Continued on next page...

CONFIDENTIAL VALIDATION REPORT

Continued from previous page...

Mol	Chain	Residues	Atoms	ZeroOcc	AltConf
3	A	1	Total C O<br>4 2 2	0	0
3	A	1	Total C O<br>4 2 2	0	0
3	A	1	Total C O<br>4 2 2	0	0

- Molecule 4 is water.

Mol	Chain	Residues	Atoms	ZeroOcc	AltConf
4	A	56	Total O<br>56 56	0	0

CONFIDENTIAL VALIDATION REPORT

3 Residue-property plots (i)

These plots are drawn for all protein, RNA, DNA and oligosaccharide chains in the entry. The first graphic for a chain summarises the proportions of the various outlier classes displayed in the second graphic. The second graphic shows the sequence view annotated by issues in geometry and electron density. Residues are color-coded according to the number of geometric quality criteria for which they contain at least one outlier: green = 0, yellow = 1, orange = 2 and red = 3 or more. A red dot above a residue indicates a poor fit to the electron density (RSRZ > 2). Stretches of 2 or more consecutive residues without any outlier are shown as a green connector. Residues present in the sample, but not in the model, are shown in grey.

- Molecule 1: Phosphatidylinositol 4-phosphate 3-kinase C2 domain-containing subunit alpha,Phosphatidylinositol 4-phosphate 3-kinase C2 domain-containing subunit alpha

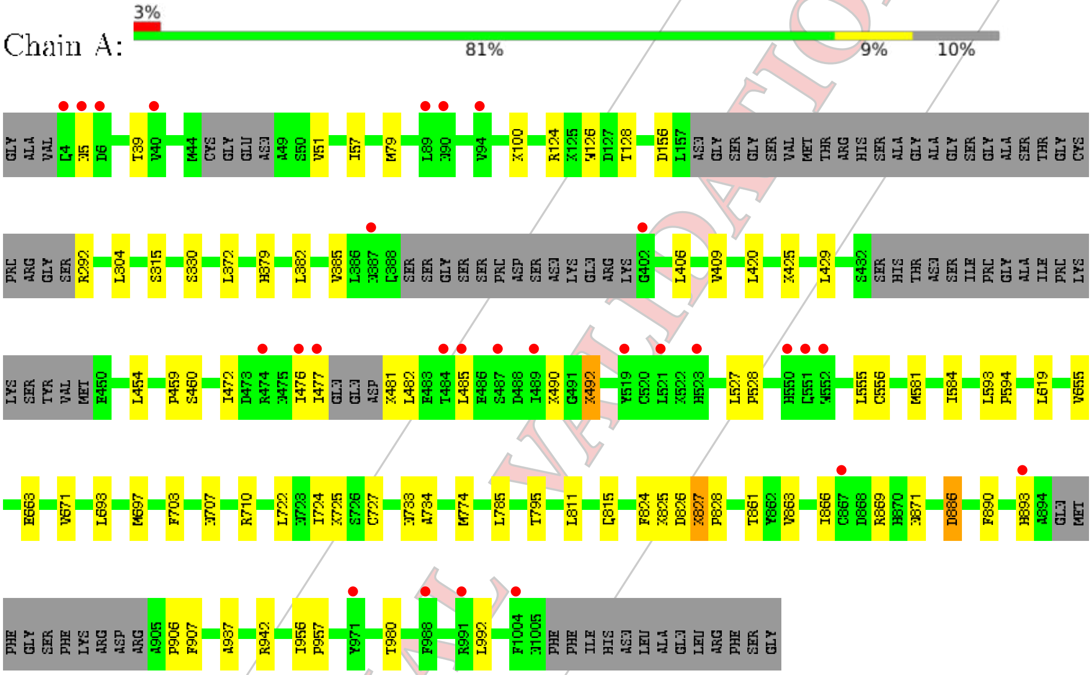

CONFIDENTIAL VALIDATION REPORT

4 Data and refinement statistics (i)

Property	Value	Source
Space group	P 21 21 21	Depositor
Cell constants<br>a, b, c, α, β, γ	55.94Å 135.40Å 151.60Å<br>90.00° 90.00° 90.00°	Depositor
Resolution (Å)	48.94 – 2.65<br>48.94 – 2.65	Depositor<br>EDS
% Data completeness<br>(in resolution range)	96.2 (48.94-2.65)<br>96.2 (48.94-2.65)	Depositor<br>EDS
R_{merge}	(Not available)	Depositor
R_{sym}	(Not available)	Depositor
$< I/\sigma(I) >$ [1]	1.12 (at 2.65Å)	Xtriage
Refinement program	REFMAC 5.8.0258	Depositor
R, R_{free}	0.233 , 0.280<br>0.235 , 0.280	Depositor<br>DCC
R_{free} test set	1655 reflections (5.00%)	wwPDB-VP
Wilson B-factor (Å^2)	70.0	Xtriage
Anisotropy	0.359	Xtriage
Bulk solvent k_{sol}(e/Å^3), B_{sol}(Å^2)	0.29 , 31.6	EDS
L-test for twinning[2]	$< \|L\| >$ = 0.49, $< L^2 >$ = 0.32	Xtriage
Estimated twinning fraction	No twinning to report.	Xtriage
F_o,F_c correlation	0.94	EDS
Total number of atoms	6642	wwPDB-VP
Average B, all atoms (Å^2)	80.0	wwPDB-VP

Xtriage's analysis on translational NCS is as follows: *The largest off-origin peak in the Patterson function is 4.57% of the height of the origin peak. No significant pseudotranslation is detected.*

[1] Intensities estimated from amplitudes.
[2] Theoretical values of $< |L| >$, $< L^2 >$ for acentric reflections are 0.5, 0.333 respectively for untwinned datasets, and 0.375, 0.2 for perfectly twinned datasets.

CONFIDENTIAL VALIDATION REPORT

5 Model quality (i)

5.1 Standard geometry (i)

Bond lengths and bond angles in the following residue types are not validated in this section: 090, EDO

The Z score for a bond length (or angle) is the number of standard deviations the observed value is removed from the expected value. A bond length (or angle) with $|Z| > 5$ is considered an outlier worth inspection. RMSZ is the root-mean-square of all Z scores of the bond lengths (or angles).

Mol	Chain	Bond lengths		Bond angles	
		RMSZ	#|Z| >5	RMSZ	#|Z| >5
1	A	0.65	0/6676	0.71	0/9034

There are no bond length outliers.

There are no bond angle outliers.

There are no chirality outliers.

There are no planarity outliers.

5.2 Too-close contacts (i)

In the following table, the Non-H and H(model) columns list the number of non-hydrogen atoms and hydrogen atoms in the chain respectively. The H(added) column lists the number of hydrogen atoms added and optimized by MolProbity. The Clashes column lists the number of clashes within the asymmetric unit, whereas Symm-Clashes lists symmetry-related clashes.

Mol	Chain	Non-H	H(model)	H(added)	Clashes	Symm-Clashes
1	A	6540	0	6610	36	0
2	A	26	0	16	0	0
3	A	20	0	30	3	0
4	A	56	0	0	0	0
All	All	6642	0	6656	36	0

The all-atom clashscore is defined as the number of clashes found per 1000 atoms (including hydrogen atoms). The all-atom clashscore for this structure is 3.

All (36) close contacts within the same asymmetric unit are listed below, sorted by their clash magnitude.

CONFIDENTIAL VALIDATION REPORT

Atom-1	Atom-2	Interatomic distance (Å)	Clash overlap (Å)
1:A:866:ILE:HG22	1:A:869:ARG:HD3	1.66	0.76
1:A:722:LEU:HD21	1:A:727:CYS:SG	2.28	0.73
1:A:472:ILE:HD12	1:A:472:ILE:H	1.58	0.68
1:A:51:VAL:HG11	1:A:124:ARG:HD3	1.76	0.67
1:A:420:LEU:HD23	3:A:1105:EDO:H11	1.79	0.64
1:A:866:ILE:CG2	1:A:869:ARG:HD3	2.30	0.60
1:A:906:PRO:HG3	1:A:992:LEU:HD22	1.85	0.59
1:A:485:LEU:HG	1:A:490:LYS:HE3	1.87	0.57
1:A:481:LYS:HG3	1:A:482:LEU:HD22	1.88	0.56
1:A:827:LYS:N	1:A:828:PRO:CD	2.69	0.55
1:A:827:LYS:N	1:A:828:PRO:HD2	2.22	0.55
1:A:671:VAL:HG11	1:A:795:THR:HB	1.89	0.54
1:A:619:LEU:HD21	1:A:655:VAL:HG13	1.90	0.53
1:A:861:THR:HG21	1:A:907:PHE:HB3	1.94	0.50
1:A:703:PHE:O	1:A:707:ASN:HB2	2.14	0.47
1:A:811:LEU:O	1:A:815:GLN:HG3	2.15	0.47
1:A:477:ILE:N	1:A:477:ILE:HD12	2.30	0.47
1:A:663:GLU:HG2	1:A:710:ARG:HD2	1.98	0.45
1:A:869:ARG:NH1	1:A:886:ASP:O	2.44	0.45
1:A:733:ASN:O	1:A:734:ALA:HB3	2.17	0.45
1:A:382:LEU:CD2	1:A:406:LEU:HD12	2.47	0.45
1:A:492:LYS:HD2	1:A:492:LYS:N	2.31	0.45
1:A:956:ILE:N	1:A:957:PRO:HD2	2.31	0.45
1:A:126:TRP:HB2	1:A:128:THR:HG23	2.00	0.44
1:A:581:MET:O	1:A:584:ILE:HG22	2.17	0.44
1:A:409:VAL:CG1	1:A:429:LEU:HB3	2.48	0.43
1:A:942:ARG:NH2	1:A:980:THR:O	2.52	0.43
1:A:527:LEU:HB3	1:A:528:PRO:HD3	2.00	0.43
1:A:304:LEU:HD13	1:A:372:LEU:HD11	2.02	0.42
1:A:459:PRO:O	3:A:1105:EDO:H21	2.19	0.41
1:A:693:LEU:HD23	1:A:697:MET:HG2	2.01	0.41
1:A:420:LEU:HD23	3:A:1105:EDO:C1	2.48	0.40
1:A:330:SER:OG	1:A:379:HIS:HB2	2.21	0.40
1:A:593:LEU:N	1:A:594:PRO:CD	2.84	0.40
1:A:774:MET:HE1	1:A:863:VAL:CG1	2.51	0.40
1:A:785:LEU:HD11	1:A:937:ALA:HB2	2.02	0.40

There are no symmetry-related clashes.

CONFIDENTIAL VALIDATION REPORT

5.3 Torsion angles (i)

5.3.1 Protein backbone (i)

In the following table, the Percentiles column shows the percent Ramachandran outliers of the chain as a percentile score with respect to all X-ray entries followed by that with respect to entries of similar resolution.

The Analysed column shows the number of residues for which the backbone conformation was analysed, and the total number of residues.

Mol	Chain	Analysed	Favoured	Allowed	Outliers	Percentiles	
1	A	808/910 (89%)	782 (97%)	26 (3%)	0	100	100

There are no Ramachandran outliers to report.

5.3.2 Protein sidechains (i)

In the following table, the Percentiles column shows the percent sidechain outliers of the chain as a percentile score with respect to all X-ray entries followed by that with respect to entries of similar resolution.

The Analysed column shows the number of residues for which the sidechain conformation was analysed, and the total number of residues.

Mol	Chain	Analysed	Rotameric	Outliers	Percentiles	
1	A	730/800 (91%)	704 (96%)	26 (4%)	35	51

All (26) residues with a non-rotameric sidechain are listed below:

Mol	Chain	Res	Type
1	A	5	ASN
1	A	39	THR
1	A	57	ILE
1	A	79	MET
1	A	100	LYS
1	A	156	ASP
1	A	292	ARG
1	A	315	SER
1	A	385	VAL
1	A	425	LYS
1	A	454	LEU
1	A	460	SER
1	A	476	ILE

Continued on next page...

CONFIDENTIAL VALIDATION REPORT

Continued from previous page...

Mol	Chain	Res	Type
1	A	492	LYS
1	A	555	LEU
1	A	556	CYS
1	A	724	ILE
1	A	725	LYS
1	A	824	PHE
1	A	825	LYS
1	A	826	ASP
1	A	827	LYS
1	A	871	ASN
1	A	886	ASP
1	A	890	PHE
1	A	893	HIS

Sometimes sidechains can be flipped to improve hydrogen bonding and reduce clashes. All (4) such sidechains are listed below:

Mol	Chain	Res	Type
1	A	41	GLN
1	A	109	GLN
1	A	312	HIS
1	A	944	GLN

5.3.3 RNA (i)

There are no RNA molecules in this entry.

5.4 Non-standard residues in protein, DNA, RNA chains (i)

There are no non-standard protein/DNA/RNA residues in this entry.

5.5 Carbohydrates (i)

There are no monosaccharides in this entry.

5.6 Ligand geometry (i)

6 ligands are modelled in this entry.

CONFIDENTIAL VALIDATION REPORT

In the following table, the Counts columns list the number of bonds (or angles) for which Mogul statistics could be retrieved, the number of bonds (or angles) that are observed in the model and the number of bonds (or angles) that are defined in the Chemical Component Dictionary. The Link column lists molecule types, if any, to which the group is linked. The Z score for a bond length (or angle) is the number of standard deviations the observed value is removed from the expected value. A bond length (or angle) with $|Z| > 2$ is considered an outlier worth inspection. RMSZ is the root-mean-square of all Z scores of the bond lengths (or angles).

Mol	Type	Chain	Res	Link	Bond lengths			Bond angles		
					Counts	RMSZ	$\#\|Z\| > 2$	Counts	RMSZ	$\#\|Z\| > 2$
3	EDO	A	1103	-	3,3,3	0.05	0	2,2,2	0.23	0
3	EDO	A	1104	-	3,3,3	0.06	0	2,2,2	0.20	0
2	090	A	1101	-	25,29,29	1.92	6 (24%)	31,41,41	4.97	9 (29%)
3	EDO	A	1105	-	3,3,3	0.10	0	2,2,2	0.37	0
3	EDO	A	1106	-	3,3,3	0.08	0	2,2,2	0.24	0
3	EDO	A	1102	-	3,3,3	0.06	0	2,2,2	0.21	0

In the following table, the Chirals column lists the number of chiral outliers, the number of chiral centers analysed, the number of these observed in the model and the number defined in the Chemical Component Dictionary. Similar counts are reported in the Torsion and Rings columns. '-' means no outliers of that kind were identified.

Mol	Type	Chain	Res	Link	Chirals	Torsions	Rings
3	EDO	A	1103	-	-	1/1/1/1	-
3	EDO	A	1104	-	-	0/1/1/1	-
2	090	A	1101	-	-	2/12/12/12	0/4/4/4
3	EDO	A	1105	-	-	1/1/1/1	-
3	EDO	A	1106	-	-	0/1/1/1	-
3	EDO	A	1102	-	-	1/1/1/1	-

All (6) bond length outliers are listed below:

Mol	Chain	Res	Type	Atoms	Z	Observed(Å)	Ideal(Å)
2	A	1101	090	CAE-CAK	-6.06	1.37	1.41
2	A	1101	090	CAO-NAN	3.70	1.45	1.35
2	A	1101	090	CAF-NAA	3.00	1.36	1.33
2	A	1101	090	CAM-NAN	2.40	1.45	1.36
2	A	1101	090	OAU-CAO	-2.21	1.18	1.23
2	A	1101	090	OAS-CAI	2.02	1.40	1.37

All (9) bond angle outliers are listed below:

Mol	Chain	Res	Type	Atoms	Z	Observed(°)	Ideal(°)
2	A	1101	090	OAT-CAJ-CAK	-17.77	89.94	120.20

Continued on next page...

CONFIDENTIAL VALIDATION REPORT

Continued from previous page...

Mol	Chain	Res	Type	Atoms	Z	Observed(°)	Ideal(°)
2	A	1101	090	OAS-CAI-CAJ	-12.49	98.98	116.49
2	A	1101	090	OAT-CAJ-CAI	12.01	149.50	120.84
2	A	1101	090	OAS-CAI-CAC	9.46	140.58	124.37
2	A	1101	090	CAE-CAK-NAL	-3.17	118.95	123.33
2	A	1101	090	CAD-CAE-CAF	-3.05	118.21	122.55
2	A	1101	090	CAJ-CAK-NAL	2.82	123.33	118.98
2	A	1101	090	CAY-OAS-CAI	-2.52	113.72	117.53
2	A	1101	090	CAD-CAE-CAK	2.22	121.19	117.59

There are no chirality outliers.

All (5) torsion outliers are listed below:

Mol	Chain	Res	Type	Atoms
2	A	1101	090	NAL-CAM-NAN-CAO
2	A	1101	090	NAG-CAM-NAN-CAO
3	A	1103	EDO	O1-C1-C2-O2
3	A	1105	EDO	O1-C1-C2-O2
3	A	1102	EDO	O1-C1-C2-O2

There are no ring outliers.

1 monomer is involved in 3 short contacts:

Mol	Chain	Res	Type	Clashes	Symm-Clashes
3	A	1105	EDO	3	0

The following is a two-dimensional graphical depiction of Mogul quality analysis of bond lengths, bond angles, torsion angles, and ring geometry for all instances of the Ligand of Interest. In addition, ligands with molecular weight > 250 and outliers as shown on the validation Tables will also be included. For torsion angles, if less then 5% of the Mogul distribution of torsion angles is within 10 degrees of the torsion angle in question, then that torsion angle is considered an outlier. Any bond that is central to one or more torsion angles identified as an outlier by Mogul will be highlighted in the graph. For rings, the root-mean-square deviation (RMSD) between the ring in question and similar rings identified by Mogul is calculated over all ring torsion angles. If the average RMSD is greater than 60 degrees and the minimal RMSD between the ring in question and any Mogul-identified rings is also greater than 60 degrees, then that ring is considered an outlier. The outliers are highlighted in purple. The color gray indicates Mogul did not find sufficient equivalents in the CSD to analyse the geometry.

CONFIDENTIAL VALIDATION REPORT

Ligand 090 A 1101

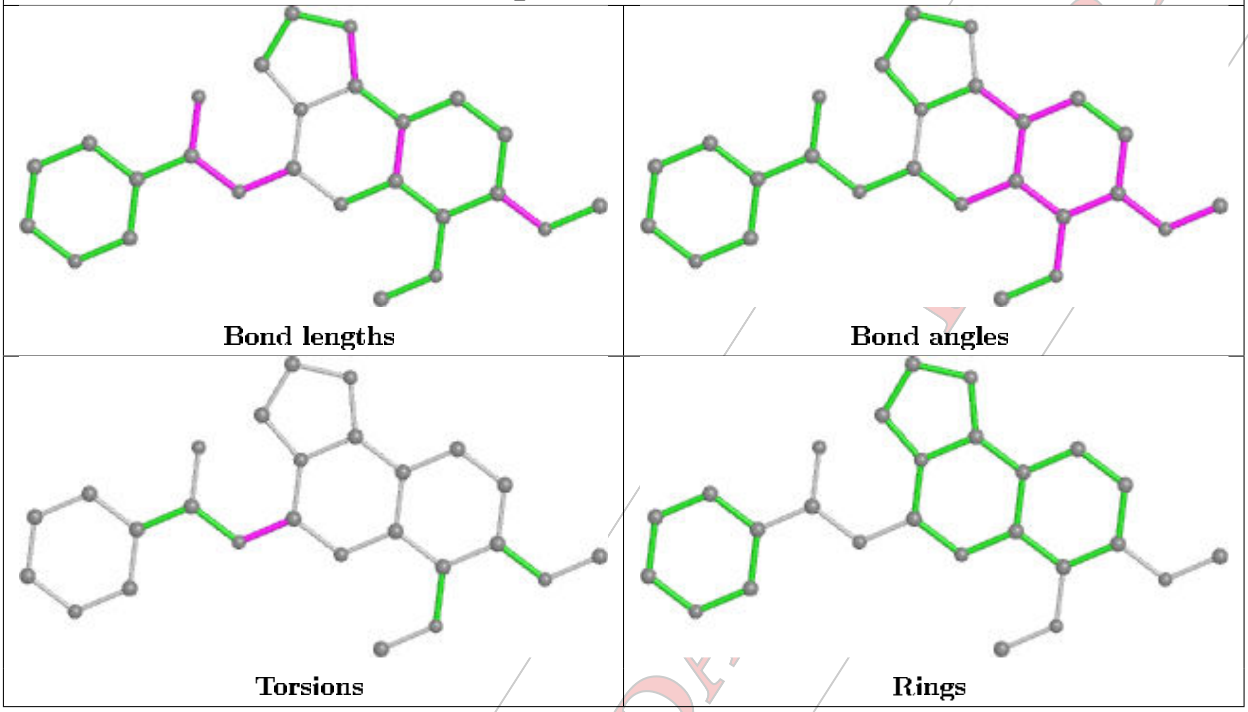

Bond lengths | Bond angles | Torsions | Rings

5.7 Other polymers (i)

There are no such residues in this entry.

5.8 Polymer linkage issues (i)

There are no chain breaks in this entry.

CONFIDENTIAL VALIDATION REPORT

6 Fit of model and data (i)

6.1 Protein, DNA and RNA chains (i)

In the following table, the column labelled '#RSRZ> 2' contains the number (and percentage) of RSRZ outliers, followed by percent RSRZ outliers for the chain as percentile scores relative to all X-ray entries and entries of similar resolution. The OWAB column contains the minimum, median, 95th percentile and maximum values of the occupancy-weighted average B-factor per residue. The column labelled 'Q< 0.9' lists the number of (and percentage) of residues with an average occupancy less than 0.9.

Mol	Chain	Analysed	<RSRZ>	#RSRZ>2	OWAB(Å^2)	Q<0.9
1	A	821/910 (90%)	0.31	28 (3%) 45 41	51, 76, 118, 159	0

All (28) RSRZ outliers are listed below:

Mol	Chain	Res	Type	RSRZ
1	A	485	LEU	4.6
1	A	893	HIS	3.8
1	A	476	ILE	3.3
1	A	991	ARG	3.3
1	A	489	ILE	3.3
1	A	89	LEU	3.2
1	A	988	PHE	3.1
1	A	1004	PHE	3.1
1	A	477	ILE	2.9
1	A	487	SER	2.8
1	A	523	HIS	2.8
1	A	90	ASN	2.6
1	A	551	GLN	2.5
1	A	519	TYR	2.5
1	A	4	GLN	2.5
1	A	484	THR	2.4
1	A	550	HIS	2.4
1	A	867	CYS	2.4
1	A	94	VAL	2.4
1	A	6	ASP	2.3
1	A	402	GLY	2.3
1	A	5	ASN	2.2
1	A	552	TRP	2.1
1	A	387	ASN	2.1
1	A	474	ARG	2.1
1	A	521	LEU	2.0
1	A	971	TYR	2.0

Continued on next page...

CONFIDENTIAL VALIDATION REPORT

Continued from previous page...

Mol	Chain	Res	Type	RSRZ
1	A	40	VAL	2.0

6.2 Non-standard residues in protein, DNA, RNA chains (i)

There are no non-standard protein/DNA/RNA residues in this entry.

6.3 Carbohydrates (i)

There are no monosaccharides in this entry.

6.4 Ligands (i)

In the following table, the Atoms column lists the number of modelled atoms in the group and the number defined in the chemical component dictionary. The B-factors column lists the minimum, median, 95th percentile and maximum values of B factors of atoms in the group. The column labelled 'Q< 0.9' lists the number of atoms with occupancy less than 0.9.

Mol	Type	Chain	Res	Atoms	RSCC	RSR	B-factors(Å^2)	Q<0.9
3	EDO	A	1106	4/4	0.58	0.45	110,110,110,111	0
3	EDO	A	1102	4/4	0.65	0.48	98,99,100,102	0
3	EDO	A	1105	4/4	0.66	0.26	92,92,92,93	0
3	EDO	A	1103	4/4	0.83	0.40	87,87,87,87	0
2	090	A	1101	26/26	0.86	0.23	94,99,104,106	0
3	EDO	A	1104	4/4	0.88	0.23	94,95,95,95	0

The following is a graphical depiction of the model fit to experimental electron density of all instances of the Ligand of Interest. In addition, ligands with molecular weight > 250 and outliers as shown on the geometry validation Tables will also be included. Each fit is shown from different orientation to approximate a three-dimensional view.

CONFIDENTIAL VALIDATION REPORT

Electron density around 090 A 1101:

2mF$_o$-DF$_c$ (at 0.7 rmsd) in gray
mF$_o$-DF$_c$ (at 3 rmsd) in purple (negative) and green (positive)

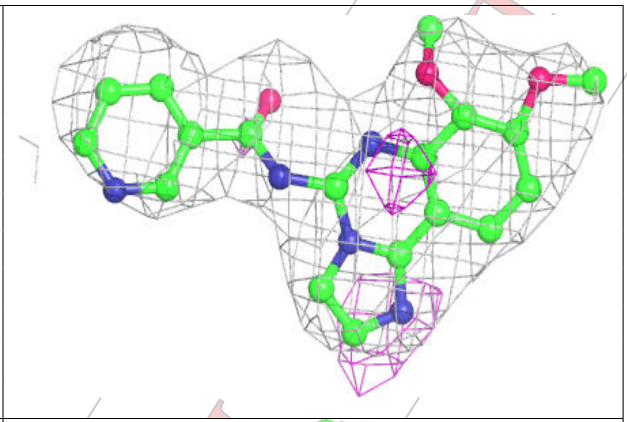

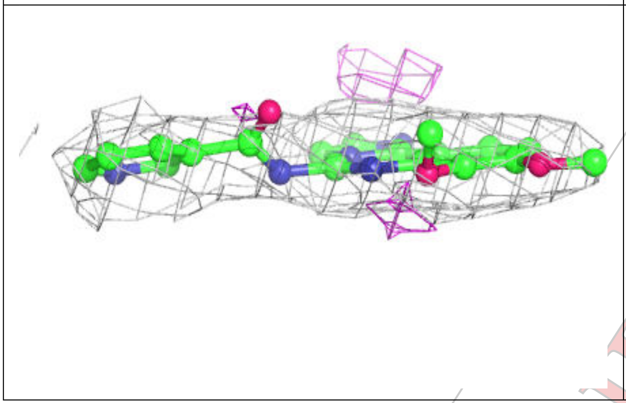

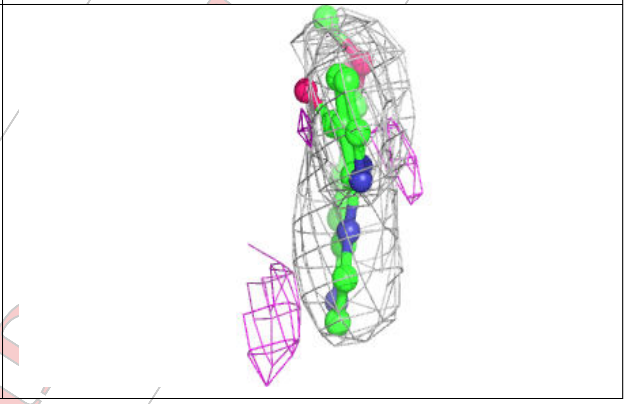

6.5 Other polymers (i)

There are no such residues in this entry.

CONFIDENTIAL

Full wwPDB EM Validation Report (i)

Jan 18, 2021 – 03:41 PM GMT

EMDB ID	:	EMD-12191
Title	:	Cryo-EM structure of the Phosphatidylinositol 3-kinase type 2a (PI3KC2a) of the class II PI3K family
Deposited on	:	2021-01-12
Resolution	:	4.40 Å(reported)

This is a Full wwPDB EM Validation Report.

This report is produced by the wwPDB biocuration pipeline after annotation of the structure.

We welcome your comments at *validation@mail.wwpdb.org*
A user guide is available at
https://www.wwpdb.org/validation/2017/EMMapValidationReportHelp
with specific help available everywhere you see the (i) symbol.

CONFIDENTIAL VALIDATION REPORT

The following versions of software and data (see references (i)) were used in the production of this report:

EMDB validation analysis	:	0.0.0.dev61
Validation Pipeline (wwPDB-VP)	:	2.16

1 Experimental information (i)

Property	Value	Source
EM reconstruction method	SINGLE PARTICLE	Depositor
Imposed symmetry	POINT, C1	Depositor
Number of particles used	601000	Depositor
Resolution determination method	FSC 0.143 CUT-OFF	Depositor
CTF correction method	PHASE FLIPPING ONLY; CTF estimation was performed after motion correction. And Ctf local refine was performed after 3D reconstruction, in cryospare.	Depositor
Microscope	FEI TITAN KRIOS	Depositor
Voltage (kV)	300	Depositor
Electron dose $(e^-/\AA^2)$	60	Depositor
Minimum defocus (nm)	1200	Depositor
Maximum defocus (nm)	3300	Depositor
Magnification	105000	Depositor
Image detector	GATAN K3 (6k x 4k)	Depositor
Maximum map value	0.235	Depositor
Minimum map value	-0.098	Depositor
Average map value	0.000	Depositor
Map value standard deviation	0.010	Depositor
Recommended contour level	0.05	Depositor
Map size (Å)	217.62001, 217.62001, 217.62001	wwPDB
Map dimensions	260, 260, 260	wwPDB
Map angles (°)	90.0, 90.0, 90.0	wwPDB
Pixel spacing (Å)	0.837, 0.837, 0.837	Depositor

CONFIDENTIAL VALIDATION REPORT

2 Map visualisation (i)

This section contains visualisations of the EMDB entry EMD-12191. These allow visual inspection of the internal detail of the map and identification of artifacts.

Images derived from a raw map, generated by summing the deposited half-maps, are presented below the corresponding image components of the primary map to allow further visual inspection and comparison with those of the primary map.

2.1 Orthogonal projections (i)

2.1.1 Primary map

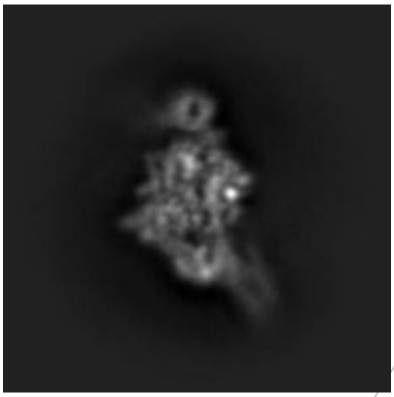

X

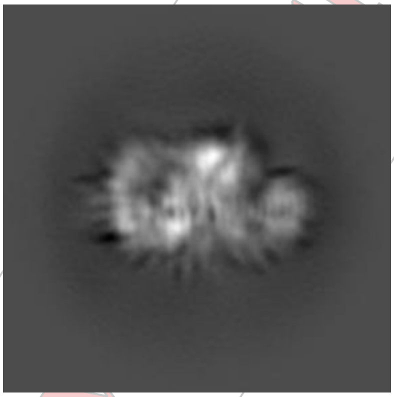

Y

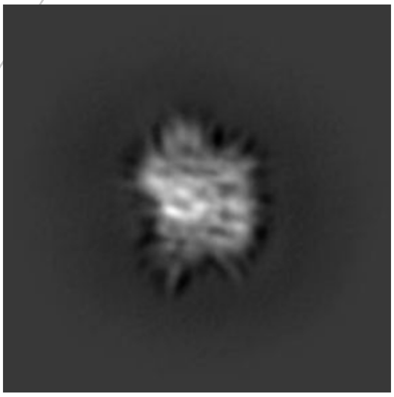

Z

2.1.2 Raw map

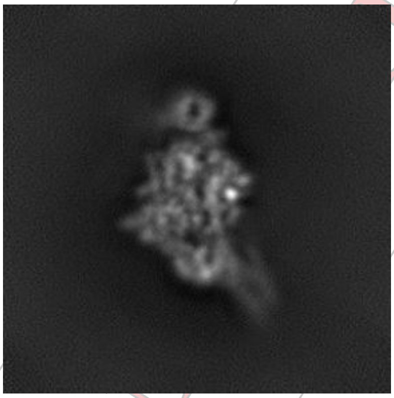

X

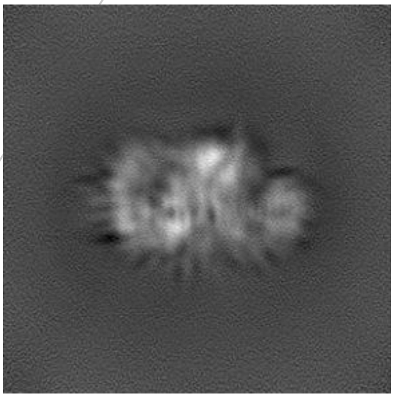

Y

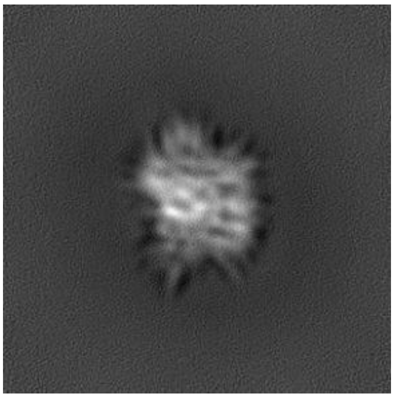

Z

The images above show the map projected in three orthogonal directions.

CONFIDENTIAL VALIDATION REPORT

2.2 Central slices (i)

2.2.1 Primary map

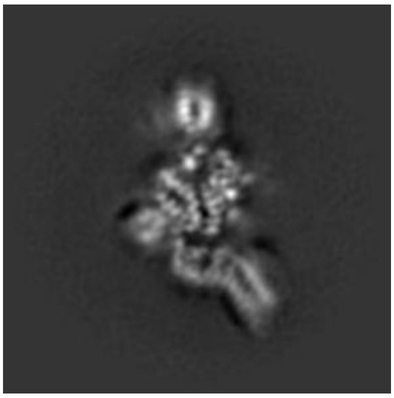

X Index: 130

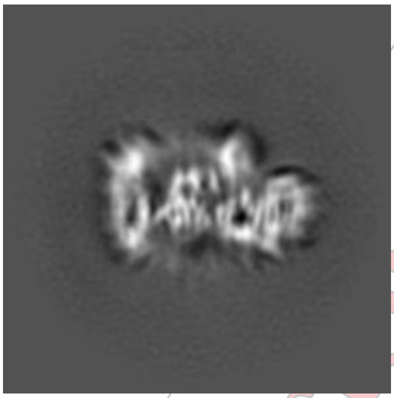

Y Index: 130

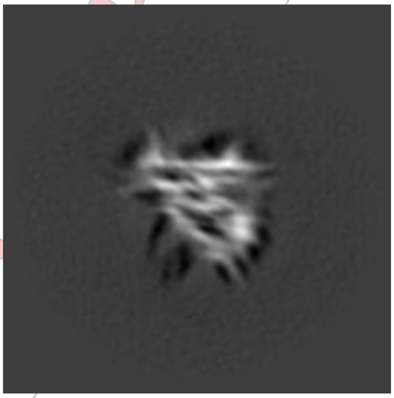

Z Index: 130

2.2.2 Raw map

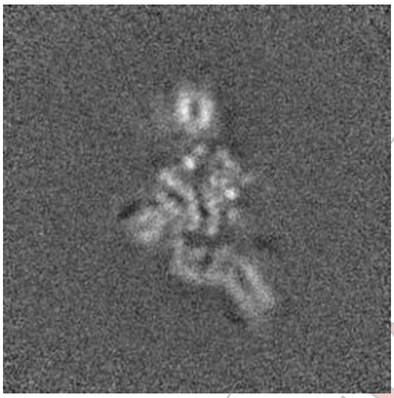

X Index: 130

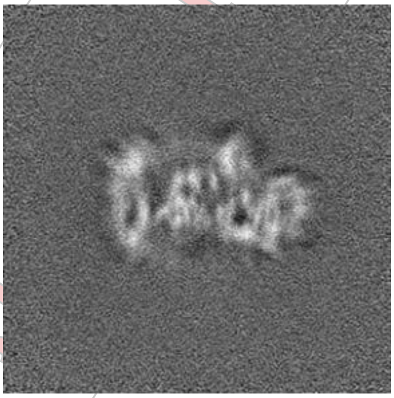

Y Index: 130

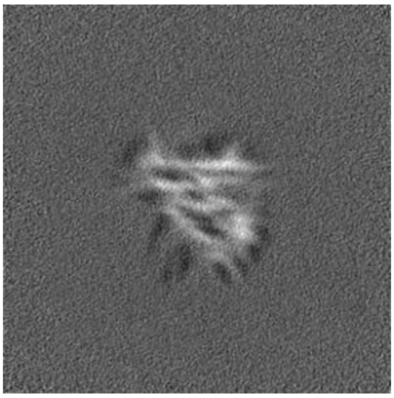

Z Index: 130

The images above show central slices of the map in three orthogonal directions.

CONFIDENTIAL VALIDATION REPORT

2.3 Largest variance slices (i)

2.3.1 Primary map

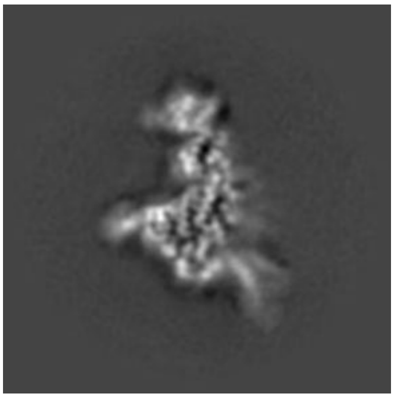
X Index: 112

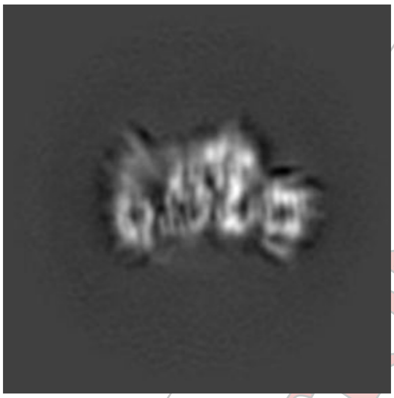
Y Index: 123

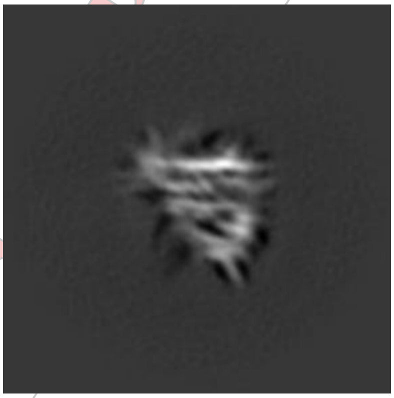
Z Index: 133

2.3.2 Raw map

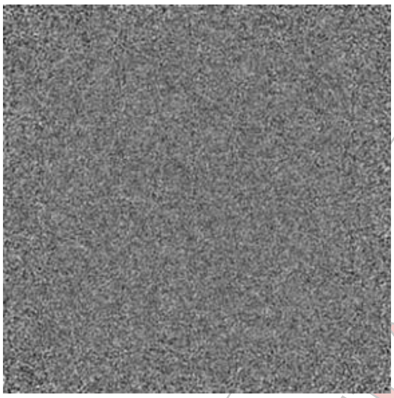
X Index: 0

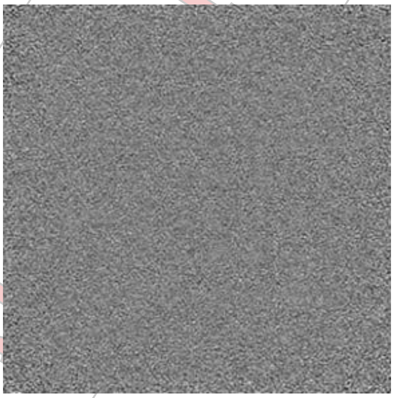
Y Index: 0

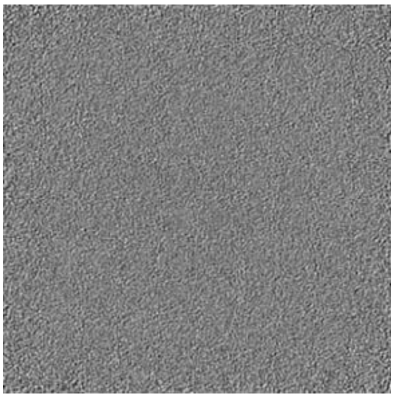
Z Index: 0

The images above show the largest variance slices of the map in three orthogonal directions.

CONFIDENTIAL VALIDATION REPORT

2.4 Orthogonal surface views (i)

2.4.1 Primary map

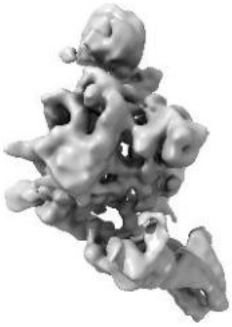
X

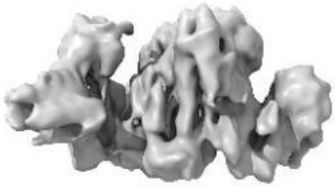
Y

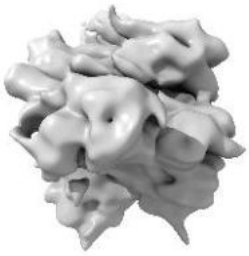
Z

The images above show the 3D surface view of the map at the recommended contour level 0.05. These images, in conjunction with the slice images, may facilitate assessment of whether an appropriate contour level has been provided.

2.4.2 Raw map

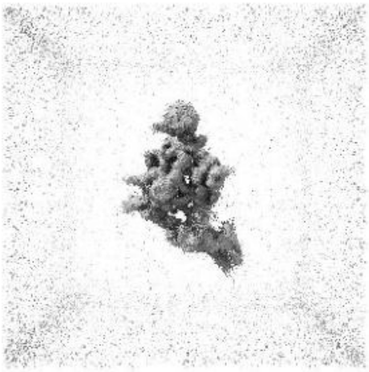
X

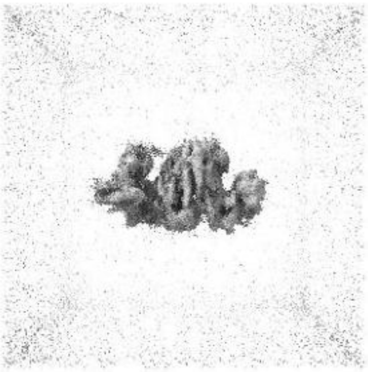
Y

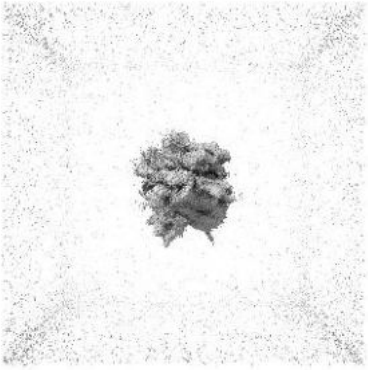
Z

These images show the 3D surface of the raw map. The raw map's contour level was selected so that its surface encloses the same volume as the primary map does at its recommended contour level.

CONFIDENTIAL VALIDATION REPORT

2.5 Mask visualisation (i)

This section shows the 3D surface view of the primary map at 50% transparency overlaid with the specified mask at 0% transparency

A mask typically either:

- Encompasses the whole structure
- Separates out a domain, a functional unit, a monomer or an area of interest from a larger structure

2.5.1 D_1292113384_em-mask-volume_P1.map.V2 (i)

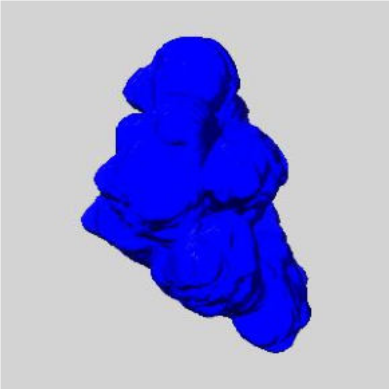

X

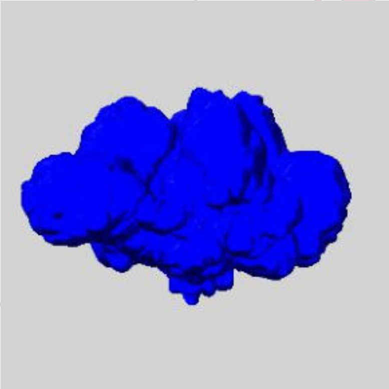

Y

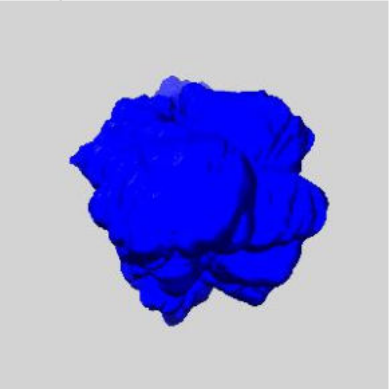

Z

CONFIDENTIAL VALIDATION REPORT

3 Map analysis ⓘ

This section contains the results of statistical analysis of the map.

3.1 Map-value distribution ⓘ

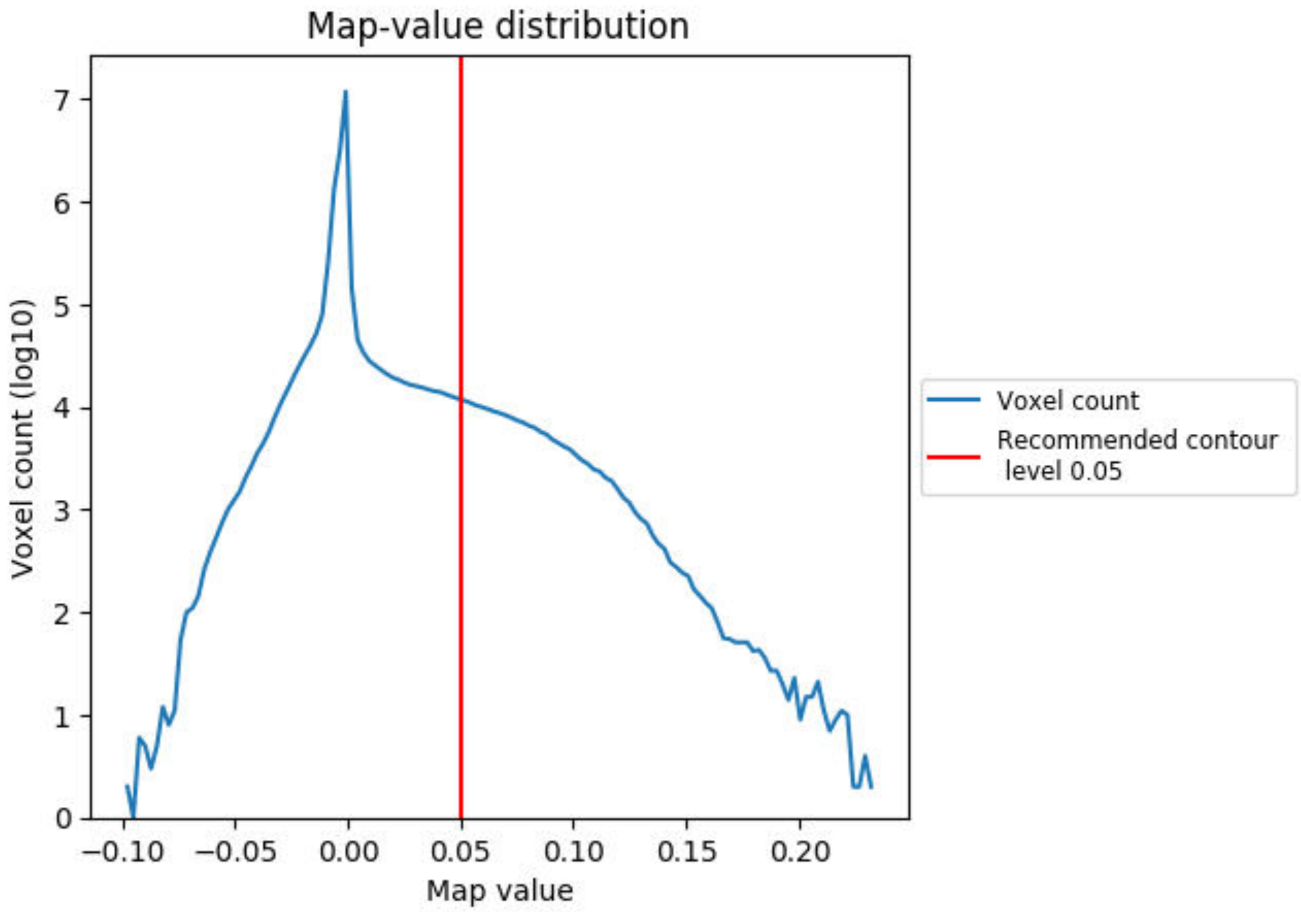

The map-value distribution is plotted in 128 intervals along the x-axis. The y-axis is logarithmic. A spike in this graph at zero usually indicates that the volume has been masked.

CONFIDENTIAL REPORT

3.2 Volume estimate (i)

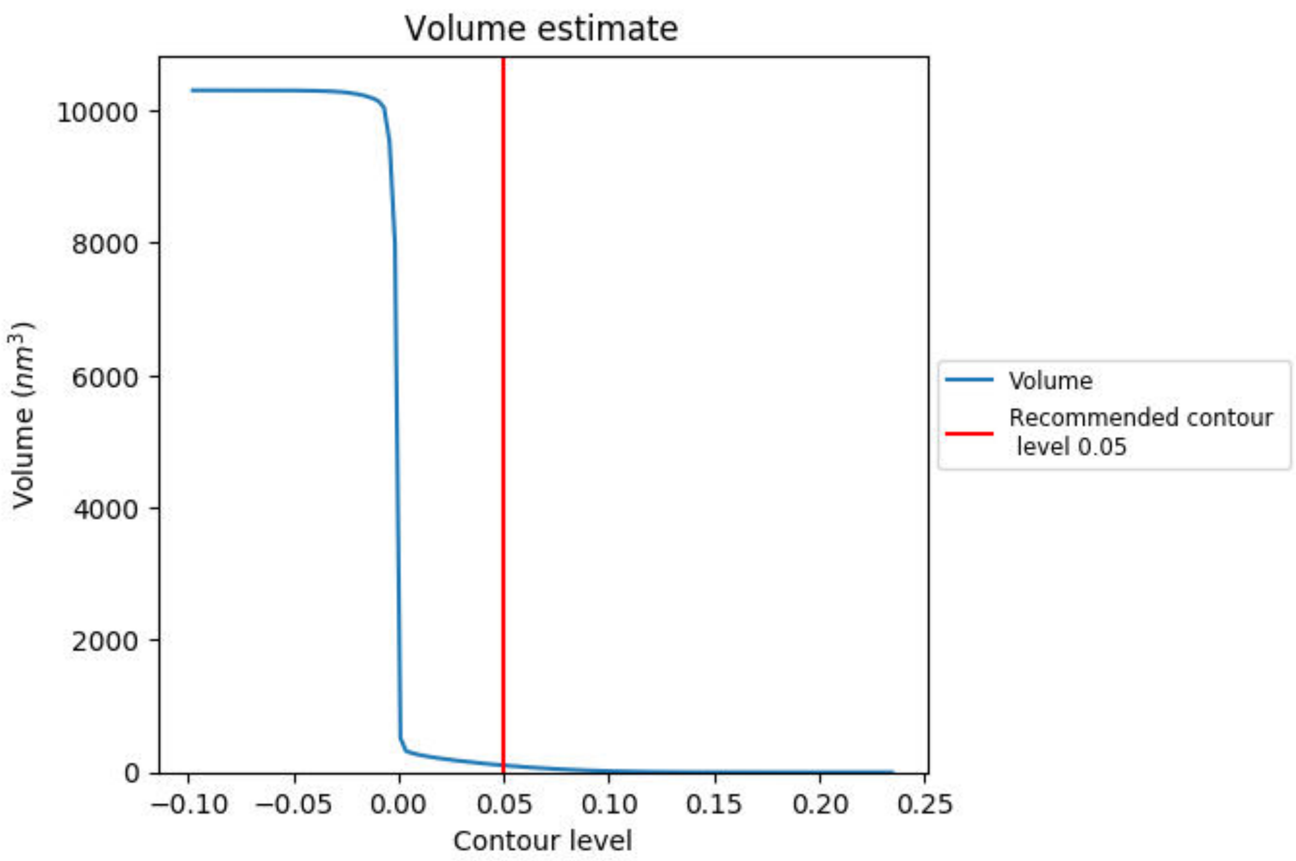

The volume at the recommended contour level is 104 nm^3; this corresponds to an approximate mass of 94 kDa.

The volume estimate graph shows how the enclosed volume varies with the contour level. The recommended contour level is shown as a vertical line and the intersection between the line and the curve gives the volume of the enclosed surface at the given level.

CONFIDENTIAL REPORT

3.3 Rotationally averaged power spectrum (i)

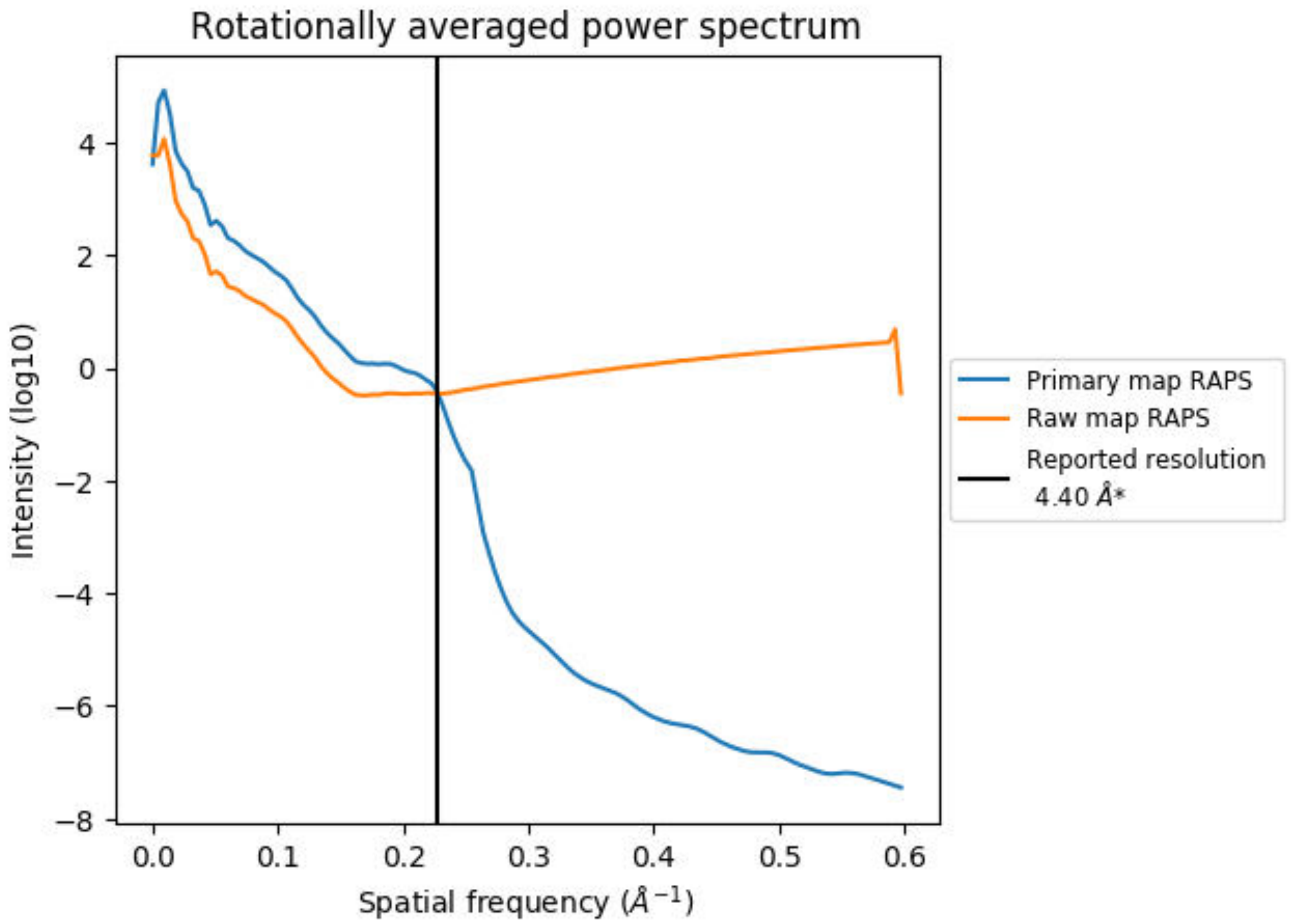

*Reported resolution corresponds to spatial frequency of 0.227 Å^{-1}

CONFIDENTIAL REPORT

4 Fourier-Shell correlation ⓘ

Fourier-Shell Correlation (FSC) is the most commonly used method to estimate the resolution of single-particle and subtomogram-averaged maps. The shape of the curve depends on the imposed symmetry, mask and whether or not the two 3D reconstructions used were processed from a common reference. The reported resolution is shown as a black line. A curve is displayed for the half-bit criterion in addition to lines showing the 0.143 gold standard cut-off and 0.5 cut-off.

4.1 FSC ⓘ

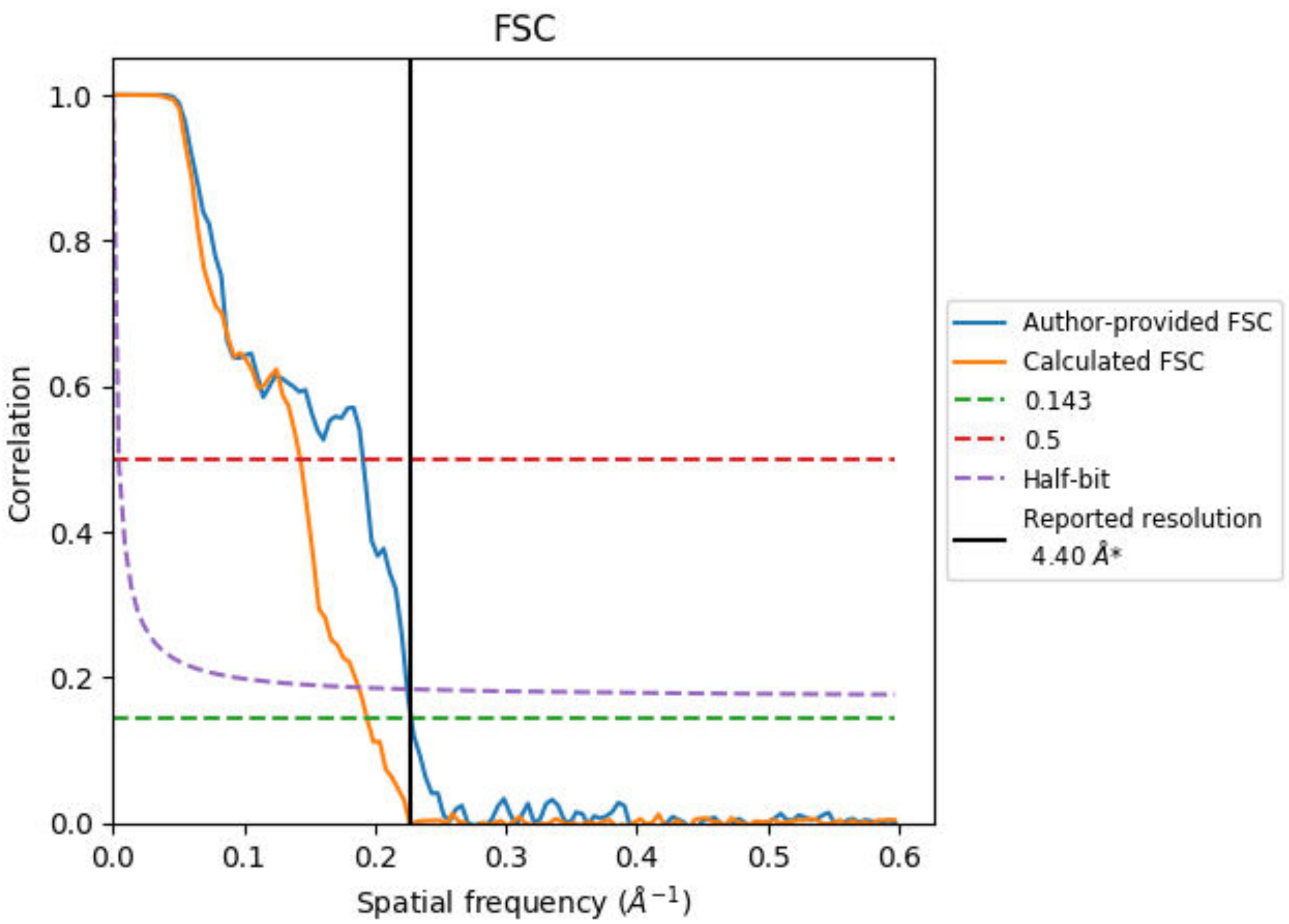

*Reported resolution corresponds to spatial frequency of 0.227 Å^{-1}

CONFIDENTIAL REPORT

4.2 Resolution estimates (i)

Resolution estimate (Å)	Estimation criterion (FSC cut-off)		
	0.143	0.5	Half-bit
Reported by author	4.40	-	-
Author-provided FSC curve	4.48	5.37	4.54
Calculated*	5.28	7.23	5.46

*Resolution estimate based on FSC curve calculated by comparison of deposited half-maps. The value from deposited half-maps intersecting FSC 0.143 CUT-OFF 5.28 differs from the reported value 4.4 by more than 10 %

CONFIDENTIAL VALIDATION REPORT
