# Supplementary material for: Structural basis of phosphatidylinositol 3-kinase C2α function
Source: Nat Struct Mol Biol. 2022 Mar 7;29(3):218–28. doi: 10.1038/s41594-022-00730-w (PMC8930771; doi:10.1038/s41594-022-00730-w)
Supplement: Supplementary file 2 — Reporting Summary [file 41594_2022_730_MOESM2_ESM.pdf]

nature research

Corresponding author(s): Wen-Ting Lo & Volker Haucke

Last updated by author(s): Dec, 09, 2021

Reporting Summary

Nature Research wishes to improve the reproducibility of the work that we publish. This form provides structure for consistency and transparency in reporting. For further information on Nature Research policies, see our Editorial Policies and the Editorial Policy Checklist.

Statistics

For all statistical analyses, confirm that the following items are present in the figure legend, table legend, main text, or Methods section.

n/a	Confirmed	
☐	☒	The exact sample size (*n*) for each experimental group/condition, given as a discrete number and unit of measurement
☐	☒	A statement on whether measurements were taken from distinct samples or whether the same sample was measured repeatedly
☐	☒	The statistical test(s) used AND whether they are one- or two-sided<br>*Only common tests should be described solely by name; describe more complex techniques in the Methods section.*
☒	☐	A description of all covariates tested
☐	☒	A description of any assumptions or corrections, such as tests of normality and adjustment for multiple comparisons
☐	☒	A full description of the statistical parameters including central tendency (e.g. means) or other basic estimates (e.g. regression coefficient) AND variation (e.g. standard deviation) or associated estimates of uncertainty (e.g. confidence intervals)
☐	☒	For null hypothesis testing, the test statistic (e.g. *F*, *t*, *r*) with confidence intervals, effect sizes, degrees of freedom and *P* value noted<br>*Give P values as exact values whenever suitable.*
☒	☐	For Bayesian analysis, information on the choice of priors and Markov chain Monte Carlo settings
☒	☐	For hierarchical and complex designs, identification of the appropriate level for tests and full reporting of outcomes
☒	☐	Estimates of effect sizes (e.g. Cohen's *d*, Pearson's *r*), indicating how they were calculated

Our web collection on statistics for biologists contains articles on many of the points above.

Software and code

Policy information about availability of computer code

Data collection	Data for immunofluorescence images were acquired using FIJI Version : 2.1.0/1.53c<br>Quantiative values from gel were acquired using imageJ Version: 1.53
Data analysis	All statistical tests were performed using Graphpad Prism9<br>X-ray diffraction data were processed using XDSAPP (Sparta et al., 2016)<br>Crystal structure determination/ refinement software package: PHENIX (1.18.2), Refmac (version 5.5 and higher) in CCP4 package (version 7.1), and coot (1.0.0).<br>EM particle autopicked used Gsutomatch-0.53<br>EM 2D class averages: RELION-2.0 and higher<br>Cross-linking MS/MS data were analyzed by using XlinkX standalone<br>Structures fitting to EM map were performed with Chimera (1.15.0)

For manuscripts utilizing custom algorithms or software that are central to the research but not yet described in published literature, software must be made available to editors and reviewers. We strongly encourage code deposition in a community repository (e.g. GitHub). See the Nature Research guidelines for submitting code & software for further information.

April 2020

Data

Policy information about availability of data

All manuscripts must include a data availability statement. This statement should provide the following information, where applicable:

- Accession codes, unique identifiers, or web links for publicly available datasets
- A list of figures that have associated raw data
- A description of any restrictions on data availability

PDB coordination in this study have been deposited in PDB data bank (pdb code: 7BI2, 7BI4, 7BI6, 7BI9)
Original EM micrographs were deposited in the EMPIAR (code: EMPIAR-10665)
CryoEM data were deposited in the PDB data bank (pdb code: EMD-12191)
Cross-linked peptides were provides in Supplementary Table 3.xlsx of the manuscript
All of statistical source data and unprocessed western blots were available in source data section of the manuscript

Field-specific reporting

Please select the one below that is the best fit for your research. If you are not sure, read the appropriate sections before making your selection.

☒ Life sciences ☐ Behavioural & social sciences ☐ Ecological, evolutionary & environmental sciences

For a reference copy of the document with all sections, see nature.com/documents/nr-reporting-summary-flat.pdf

Life sciences study design

All studies must disclose on these points even when the disclosure is negative.

Sample size	Sample sizes were not chosen based on pre-specified effect size but selected based on commonly adopted standards in the field, resulting in statistically meaningful comparison. Sample size were corresponding to 20 images per sample for microscopy-based quantifications (Posor et al. 2013). Multiple independent experiments were carried out as detailed in the figure legends and Data reproducibility section within methods.
Data exclusions	No samples were excluded from analysis.
Replication	All experiments were carried out under standard and clearly defined conditions, and were replicated successfully by at least one researcher and all attempts of replication were successful. The number of replicates of each experiment is specified in the corresponding figure legend and data and reproducibility section within the Methods.
Randomization	No animals have been used for this study, and No randomization was needed for the experiment with cultured cell line as cells were passaged in the same step from one parental cell dish for all groups in each experiment.
Blinding	Immunofluorescence images were captured blindly by selecting cells in the DAPI channel or in the GFP channel (when performing knocked down and re-expressed experiments).

Reporting for specific materials, systems and methods

We require information from authors about some types of materials, experimental systems and methods used in many studies. Here, indicate whether each material, system or method listed is relevant to your study. If you are not sure if a list item applies to your research, read the appropriate section before selecting a response.

Materials & experimental systems

n/a	Involved in the study	
☐	☒	Antibodies
☐	☒	Eukaryotic cell lines
☒	☐	Palaeontology and archaeology
☒	☐	Animals and other organisms
☒	☐	Human research participants
☒	☐	Clinical data
☒	☐	Dual use research of concern

Methods

n/a	Involved in the study	
☒	☐	ChIP-seq
☒	☐	Flow cytometry
☒	☐	MRI-based neuroimaging

Antibodies

Antibodies used	Primary antibody:<br>PI(3,4)P2 antibody (mouse, dilution 1:150, Echlon Cat# Z-P034b)

	anti-GFP antibodies (Rabbit, Gulluni et al., 2017, Cancer Cell, 1:2000), TACC3 antibody (Rabbit, Cell Signaling #8069, 1: 1000)<br>Secondary antibody:<br>HRP-conjugated anti-Rabbit IgG light chain (1:5000, Jackson ImmunoResearch, 211-032-171)<br>Goat anti-mouse IgG (H+L) AF647 (1:400, Thermo Fisher, #A21237)
Validation	PI(3,4)P2 antibody and TACC3 antibody were validated by the manufacture's website<br>anti-GFP antibody was validated in Gulluni et al., 2017

Eukaryotic cell lines

Policy information about cell lines

Cell line source(s)	Cos7 and HeLa cells were obtained from ATCC.
Authentication	Cell lines from ATCC are regularly authenticated by STR profiling and were used by us without further authentication.
Mycoplasma contamination	Cell lines were regularly tested for mycoplasm contamination and were not contaminated
Commonly misidentified lines<br>(See ICLAC register)	No commonly misidentified cell lines were used in the study.
