# Supplementary figures and images for: Structural basis of phosphatidylinositol 3-kinase C2α function
Source: Nat Struct Mol Biol. 2022 Mar 7;29(3):218–28. doi: 10.1038/s41594-022-00730-w (PMC8930771; doi:10.1038/s41594-022-00730-w)

input- anti-GFP

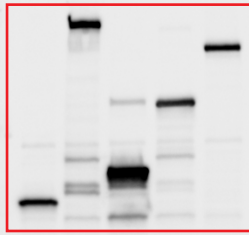

IP- anti-GFP

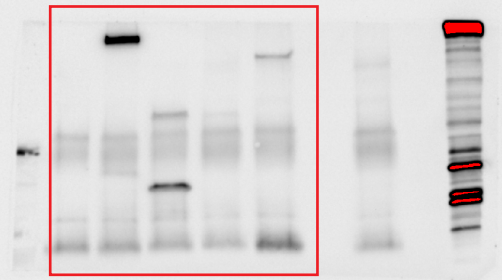

input TACC3

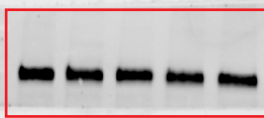

IP- TACC3

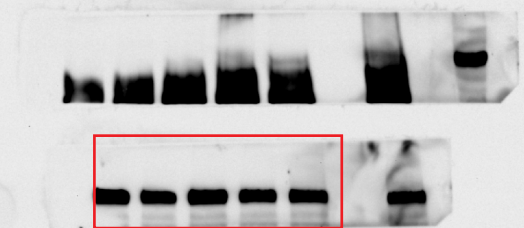

Supplement: Source Data Fig. 6d — Unprocessed blots for Fig. 6d. [file 41594_2022_730_MOESM11_ESM.pdf]

extended data Fig. 1b left

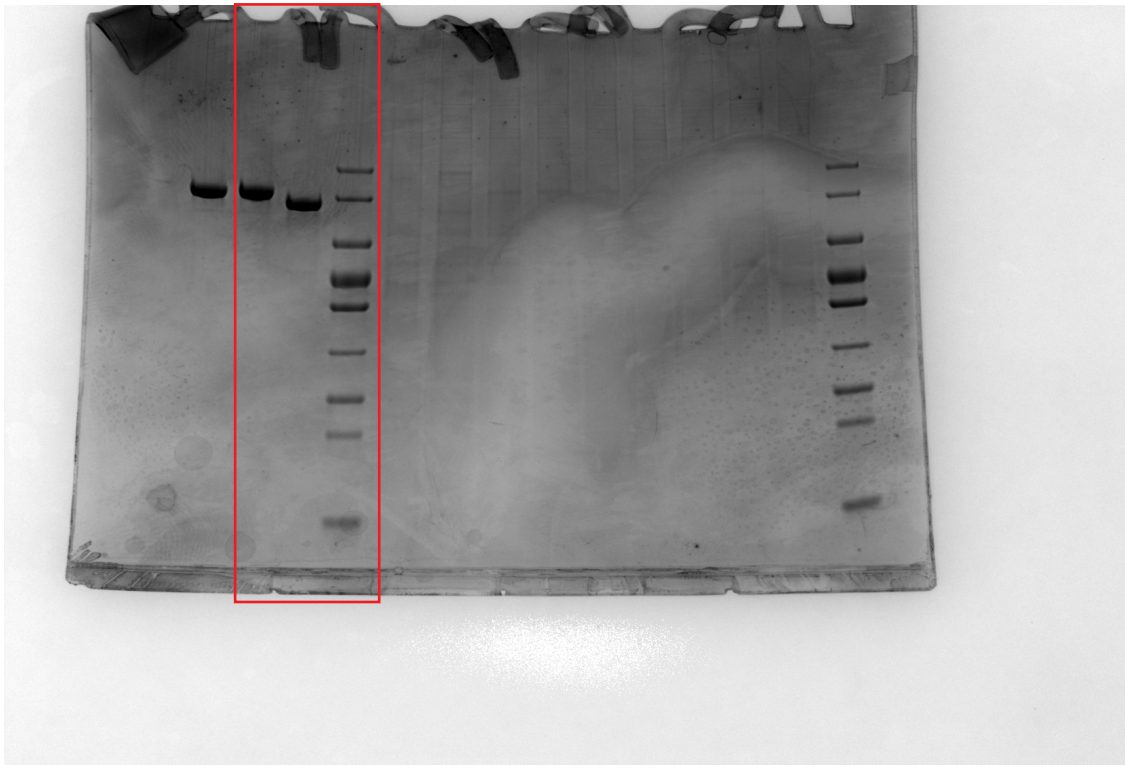

extended data Fig. 1b right

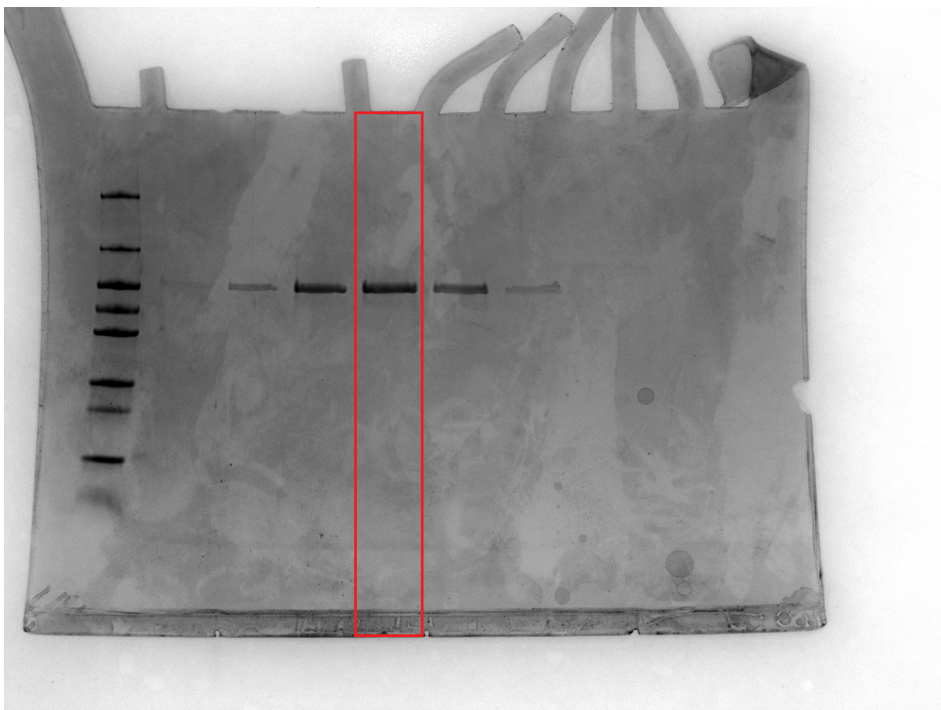

Supplement: Source Data Extended Data Fig. 1b — Uncropped gel for Extended Data Fig. 1b. [file 41594_2022_730_MOESM13_ESM.pdf]

Extend datat Fig. 6d left

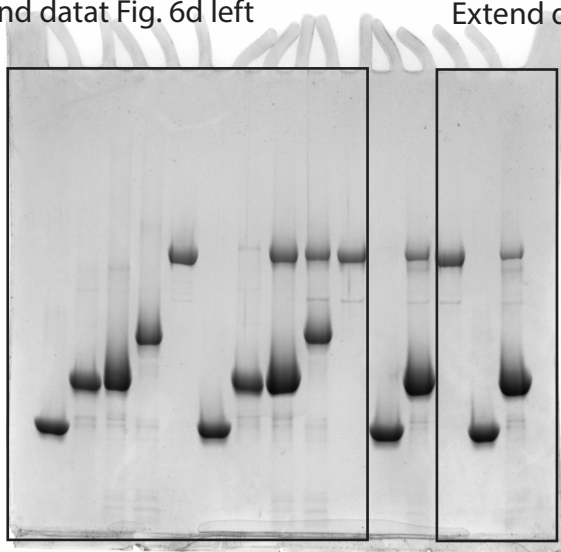

Extend datat Fig. 6d right

marker

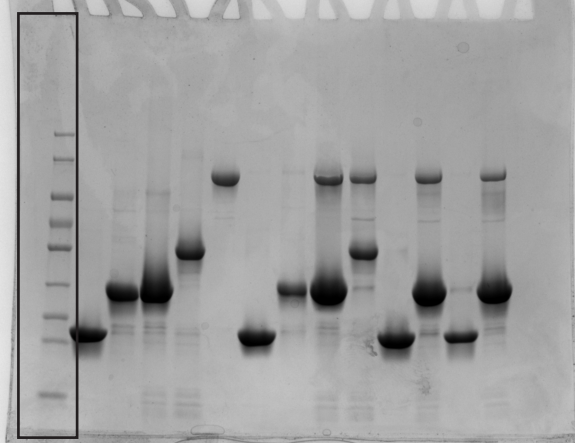

Supplement: Source Data Extended Data Fig. 6d — Uncropped gel for Extended Data Fig. 6d. [file 41594_2022_730_MOESM17_ESM.pdf]
