# Supplementary material for: Structural basis of phosphatidylinositol 3-kinase C2α function
Source: Nat Struct Mol Biol. 2022 Mar 7;29(3):218–28. doi: 10.1038/s41594-022-00730-w (PMC8930771; doi:10.1038/s41594-022-00730-w)
Supplement: Supplementary file 3 — Peer Review Information [file 41594_2022_730_MOESM3_ESM.pdf]

natureresearch

Peer Review Information

Journal: Nature Structural and Molecular Biology
Manuscript Title: Structural basis of phosphatidylinositol 3-kinase C2alpha function
Corresponding author name(s): Professor Volker Haucke, Dr Wen-Ting Lo

Reviewer Comments & Decisions:

Decision Letter, initial version:

3rd Nov 2021

Dear Dr. Haucke,

Thank you again for submitting your manuscript "Structural basis of phosphatidylinositol 3-kinase C2alpha function". I apologize for the delay in responding, which resulted from the difficulty in obtaining suitable referee reports. Nevertheless, we now have comments (below) from the 3 reviewers who evaluated your paper.

You will see that all three reviewers find that the paper is of very high technical quality and represents an important advance, and only have minor comments. Therefore we'll be happy in principle to publish it in Nature Structural & Molecular Biology, pending minor revisions to satisfy the referees' final requests and to comply with our editorial and formatting guidelines.

We are now performing detailed checks on your paper and will send you a checklist detailing our editorial and formatting requirements in about a week. Please do not upload the final materials and make any revisions until you receive this additional information from us.

To facilitate our work at this stage, we would appreciate if you could send us the main text as a word file. Please make sure to copy the NSMB account (cc'ed above).

Thank you again for your interest in Nature Structural & Molecular Biology Please do not hesitate to contact me if you have any questions.

Sincerely,

Carolina

Carolina Perdigoto, PhD
Chief Editor

Nature Structural & Molecular Biology
orcid.org/0000-0002-5783-7106

Reviewer #1 (Remarks to the Author):

The manuscript of Lo et al. represents an important step forward in understanding the mechanism of autoinhibition and activation of the class II PI3K, PIK3C2alpha. For years, reviews of PI3Ks have simply said that not much is known about these enzymes. However, with burgeoning attention on these enzymes due to their newly discovered roles in cell biology, it is a perfect time to understand how these enzymes function. Although this class of enzyme was discovered more than 20 years ago, it has resisted nearly all efforts at understanding its structure. It was difficult to express and difficult to crystallise. Lo et al. built on HDX-MS results that defined key regions in the enzyme that are disordered, and they truncated key N-terminal, C-terminal and one internal deletion that gave structures of highest resolution. With the structure in hand, they proposed how the enzyme would interact with substrate ATP and lipids. The identified key interactions that held the enzyme in an autoinhibited conformation. Using cryo-EM, they also developed an understanding of how the C-terminal C2 and PX domains maintain the enzyme in an inhibited state until bound to membranes with the correct composition. The authors also characterised inhibitors binding to the enzyme. This is the first structural insight into developing specific inhibitors of this class of enzymes. The reported structure will serve as a paradigm for the entire the class II PI3K family.

Overall, this is a timely and fascinating contribution to the field of 3-kinases. Probably the most unexpected is the structural observation of a helical bundle in the structure that has no orthologue in other classes of PI3Ks. The authors showed remarkable insight to recognise the similarity of the structure of this PIK3C2alpha domain to structures of F-actin-binding domains, and they have demonstrated that this domain is necessary and sufficient for targeting the enzyme to mitotic spindles.

The manuscript is an excellent contribution as it is. There are only a few very minor points that might need attention.

1. There is a citation 10 in the author list for Haucke, but there is no 10 footnote.
2. In the abstract, "as a paradigm for entire the class II PI3K family" should be "as a paradigm for the entire class II…"
3. In the introduction it states that PI3Ks are classified on the basis of their structural organization not and substrate specificity. They are really classified on the basis of their structures. The class I enzymes can use a range of substrates in vitro.
4. Fig 2e shows that PI4P is a better substrate than PI. Is PI significantly greater than background? (i.e., the activity that is measured in the ADP-GLO assay in the absence of a lipid substrate).
5. There is the statement on p. 5 that "H1252 within the DRH motif acts as catalytic base in lipid kinases to de-protonate the 3'-OH of the inositol substrate, a reaction aided by D1250 and D1268". This statement should either give a reference, describe the experiments that the authors carried out to come to this conclusion or it should be labelled that this is a proposal as to what is the catalytic base. The designation of the catalytic base for various lipid and protein kinases has been a contentious issue.

Reviewer #2 (Remarks to the Author):

In their manuscript, Lo et al. investigate structure and function of phosphatidylinositol 3-kinase type 2alpha (PI3KC2alpha). PI3KC2alpha belongs to the class II PI3K family that remains understudied, and despite their disease relevance, structural, functional and mechanistic details about these kinases are missing. In the present study, the authors overcome this gap of knowledge by using cryo-electron microscopy (cryo-EM) and X-ray crystallography (XRC) to study the structure of PI3KC2alpha. Moreover, chemical cross-linking coupled to mass spectrometry (XL-MS) was used to monitor conformational changes of the protein.

Overall, the manuscript is very well written and easy to follow, and results are clearly presented. The abstract provides a concise summary, and I consider the length of the text appropriate. The work provides a comprehensive overview of structural and functional investigations of the enzyme. The structures of various constructs were resolved by XRC or cryo-EM, and provide insights into the active site and the substrate binding pocket. All structural work is of high quality and was performed according to the standards of the field. Inhibition of enzymatic activity is examined by resolving the structure of the protein in complex with two small molecules, Torin-2 and PIK-90. Structural studies are complemented by functional assays, including live cell assays. Overall, this is an elegant study worthy of publication in NSMB, as it addresses a highly relevant class of enzymes.

I have been asked to comment specifically on the XL-MS part. This constitutes only a small part of the main manuscript and I have no specific concerns about how cross-linking data was used in this study. However, in my opinion, more details should be provided in the methods section even though the technique was not one of the main methods used in this work.

1. The methods section provides rather limited information about experimental procedures following the cross-linking step itself. This could be expanded further by adding more details, especially about the data analysis.

2. Extended Data Figure 6 shows in more detail how the XL-MS data were used. In this context, the authors mention in the figure legend that cross-linking assisted docking was performed using HADDOCK, this step is completely left out of the methods details and should be added.

3. In the same figure, panel (b) is the only source of information for the experimentally observed cross-links. A dedicated table listing all the identified peptide pairs would be more informative. The authors should also consider depositing the XL-MS data in a community repository.

Minor comments:

Figure 5: The legend for panel (d) and the panel itself are not completely aligned. The legend mentions an inset with a close-up view, but it seems that both independent channels and the merge show the same view?

Method section, page 28: The first sentences of the paragraph "Cryo-EM sample preparation of PI3KC2alpha-deltaN" need to be revised.

Method section, page 30: deoxicitidine > deoxycytidine

Method section, page 31: policlonal > polyclonal

Reviewer #3 (Remarks to the Author):

PI3K signaling family plays important function in lipid related cell metabolism, and contains many potential targets for therapeutic application. In this manuscript, the authors provided a thorough understanding about the unique structural details of class II PI3KC2α in multi-states, suggesting the structural mechanism of lipid-induced activation. Those novel knowledges are significant and provide immediate interest to PI3K research filed. The structure, biochemistry and cell imaging data were achieved, analyzed and presented with high quality. I don't have major concerns for publication.

Couple of minor suggestions:
(1) Please insert scale bar for all the EM images and volumes, if there is no atomic model severing as scale reference.
(2) Provide the value of elution volume for each protein in extended data fig 1
(3) To me, the resolution for cryoEM map is over-estimated (extended data fig 5). Also, you may try to throw away some particles in the dominant views and re-do the refinement to get a more isotropic map.

Author Rebuttal to Initial comments

Response to the reviewers
We would like to thank the editor as well as all three referees for their careful reading of our manuscript and for their thoughtful suggestions. We were very happy to learn that all three reviewers agree on the high general interest and timeliness of our study and for their support to publish our findings in *NSMB*. In the revised manuscript we have now addressed all of their suggestions and comments as further detailed below (our response in blue).

Reviewer #1
The manuscript of Lo et al. represents an important step forward in understanding the mechanism of autoinhibition and activation of the class II PI3K, PIK3C2alpha. For years, reviews of PI3Ks have simply said that not much is known about these enzymes. However, with burgeoning attention on these enzymes due to their newly discovered roles in cell biology, it is a perfect time to understand how these enzymes function. Although this class of enzyme was discovered more than 20 years ago, it has resisted nearly all efforts at understanding its structure. It was difficult to express and difficult to crystallise. Lo et al. built on HDX-MS results that defined key regions in the enzyme that are disordered, and they truncated key N-terminal, C-terminal and one internal deletion that gave structures of highest resolution. With the structure in hand, they proposed how the enzyme would interact with substrate ATP and lipids. The identified key interactions that held the enzyme in an autoinhibited conformation. Using cryo-EM, they also developed an understanding of how the C-terminal C2 and PX domains maintain the enzyme in an inhibited state until bound to membranes with the correct composition. The authors also characterised inhibitors binding to the enzyme. This is the first structural insight into developing specific inhibitors of this class of enzymes. The reported structure will serve as a paradigm for the entire the class II PI3K family.

Overall, this is a timely and fascinating contribution to the field of 3-kinases. Probably the most

unexpected is the structural observation of a helical bundle in the structure that has no orthologue in other classes of PI3Ks. The authors showed remarkable insight to recognise the similarity of the structure of this PIK3C2alpha domain to structures of F-actin-binding domains, and they have demonstrated that this domain is necessary and sufficient for targeting the enzyme to mitotic spindles.

The manuscript is an excellent contribution as it is. There are only a few very minor points that might need attention.
Response: We thank the referee for his/ her enthusiastic comments and for highlighting the quality and impact of our study.

1. There is a citation 10 in the author list for Haucke, but there is no 10 footnote.
Response: We thank the referee for spotting this error that has been corrected in the revised version of our Ms.

2. In the abstract, "as a paradigm for entire the class II PI3K family" should be "as a paradigm for the entire class II…"
Response: We have edited the abstract accordingly.

3. In the introduction it states that PI3Ks are classified on the basis of their structural organization not and substrate specificity. They are really classified on the basis of their structures. The class I enzymes can use a range of substrates in vitro.
Response: Thank you! We have edited the sentence as suggested.

4. Fig 2e shows that PI4P is a better substrate than PI. Is PI significantly greater than background? (i.e., the activity that is measured in the ADP-GLO assay in the absence of a lipid substrate).

Response: We routinely conduct control experiments in the absence of any lipid substrate. These data shown below show that the signal for PI as a substrate is about 6.5-fold above that obtained in the absence of lipid and is, thus, significantly above background.

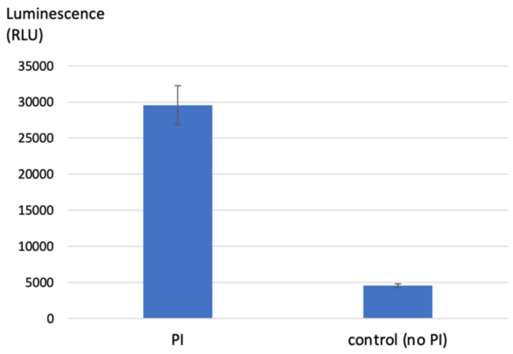

5. There is the statement on p. 5 that "H1252 within the DRH motif acts as catalytic base in lipid kinases to de-protonate the 3'-OH of the inositol substrate, a reaction aided by D1250 and D1268". This statement should either give a reference, describe the experiments that the authors carried out to come to this conclusion or it should be labelled that this is a proposal as to what is the catalytic base. The designation of the catalytic base for various lipid and protein kinases has been a contentious issue.
Response: We refer to the proposed mechanism of VPS34 from Miller et al Science (2010) [see: doi: 10.1126/science.1184429] and have added the missing reference in the revised Ms.

Reviewer #2
In their manuscript, Lo et al. investigate structure and function of phosphatidylinositol 3-kinase type 2alpha (PI3KC2alpha). PI3KC2alpha belongs to the class II PI3K family that remains understudied, and despite their disease relevance, structural, functional and mechanistic details about these kinases are missing. In the present study, the authors overcome this gap of knowledge by using cryo-electron microscopy (cryo-EM) and X-ray crystallography (XRC) to study the structure of PI3KC2alpha. Moreover, chemical cross-linking coupled to mass spectrometry (XL-MS) was used to monitor conformational changes of the protein.

Overall, the manuscript is very well written and easy to follow, and results are clearly presented. The abstract provides a concise summary, and I consider the length of the text appropriate. The work provides a comprehensive overview of structural and functional investigations of the enzyme. The structures of various constructs were resolved by XRC or cryo-EM, and provide insights into the active site and the substrate binding pocket. All structural work is of high quality and was performed according to the standards of the field. Inhibition of enzymatic activity is examined by resolving the structure of the protein in complex with two small molecules, Torin-2 and PIK-90. Structural studies are complemented by functional assays, including live cell assays. Overall, this is an elegant study worthy of publication in NSMB, as it addresses a highly relevant class of enzymes.
Response: We thank the referee for his/ her support and the endorsement to publish our work in *NSMB*.

I have been asked to comment specifically on the XL-MS part. This constitutes only a small part of the main manuscript and I have no specific concerns about how cross-linking data was used in this study. However, in my opinion, more details should be provided in the methods section even though the technique was not one of the main methods used in this work.

1. The methods section provides rather limited information about experimental procedures following the cross-linking step itself. This could be expanded further by adding more details, especially about the data analysis.
Response: We have added additional details on the experimental procedures for the MS analysis and also provide an additional reference in the revised Methods section of our paper.

2. Extended Data Figure 6 shows in more detail how the XL-MS data were used. In this context, the authors mention in the figure legend that cross-linking assisted docking was performed using HADDOCK, this step is completely left out of the methods details and should be added.
Response: We apologize for this omission. We have now added further details on the crosslinking- and EM map-assisted docking using HADDOCK in the revised Methods section.

3. In the same figure, panel (b) is the only source of information for the experimentally observed cross-links. A dedicated table listing all the identified peptide pairs would be more informative. The authors should also consider depositing the XL-MS data in a community repository.
Response: We now provide the identified crosslinked peptides in as Supplementary Table 3 in the revised version of our manuscript.

Minor comments:
Figure 5: The legend for panel (d) and the panel itself are not completely aligned. The legend mentions an inset with a close-up view, but it seems that both independent channels and the merge show the same view?
Response: Thank you for spotting the mis-alignment that has now been fixed. We have also modified the legend to this figure to make clear that only high magnification close-up views are presented to illustrate the colocalization of clathrin with $PI(3,4)P_2$, i.e. the lipid product of PI3KC2α.

Method section, page 28: The first sentences of the paragraph "Cryo-EM sample preparation of PI3KC2alpha-deltaN" need to be revised.
Response: We have revised the text to: "Purified PI3KC2$\alpha^{\Delta N}$ (0.8 mg/ml in 20 mM Tris-HCl, 100 mM NaCl at pH 7.4) were used for plunge-freezing."

Method section, page 30: deoxicitidine > deoxycytidine
Method section, page 31: policlonal > polyclonal
Response: Thank you for spotting these errors that have been corrected in the revised Ms.

Reviewer #3
PI3K signaling family plays important function in lipid related cell metabolism, and contains many potential targets for therapeutic application. In this manuscript, the authors provided a thorough understanding about the unique structural details of class II PI3KC2α in multi-states, suggesting the structural mechanism of lipid-induced activation. Those novel knowledges are significant and provide immediate interest to PI3K research filed. The structure, biochemistry and cell imaging data were achieved, analyzed and presented with high quality. I don't have major concerns for publication.
Response: We thank the referee for highlighting the quality of our study and for the endorsement to publish our findings in *NSMB*.

Couple of minor suggestions:

(1) Please insert scale bar for all the EM images and volumes, if there is no atomic model severing as scale reference.
Response: Sale bars have been added to Fig. 4a and Extended Data Fig. 5b.

(2) Provide the value of elution volume for each protein in extended data fig 1
Response: Elution volumes on the x-axis have been added to Extended Data Fig 1a as suggested.

(3) To me, the resolution for cryoEM map is over-estimated (extended data fig 5).
Response: Indeed, Extended Data Fig.5 shows the resolution is anisotropic and variable for the different domains. Still as the core domain is well ordered, the overall number is 4.4 Å. We have updated the description of the map in the main Ms text as follows to clarify this point:
"Tilted data collection followed by 2D classification resulted in a reconstruction with a nominal resolution of 4.4 Å from ~600,000 particles with significant resolution anisotropy and the highest resolution in the core domain (Extended Data Fig. 5)."

Also, you may try to throw away some particles in the dominant views and re-do the refinement to get a more isotropic map.
Response: We have already made strong efforts to improve the isotropy of the cyro-EM map. However, as anisotropy reflects an inherent property of the protein analyzed no further improvement was possible. We nonetheless are of the opinion that the data presented together with the complementary analysis by protein X-ray crystallography and XL-mass spectrometry provide important insight into PI3KC2α structure and conformational activation.

Final Decision Letter:

21st Jan 2022

Dear Dr. Haucke,

We are now happy to accept your revised paper "Structural basis of phosphatidylinositol 3-kinase C2alpha function" for publication as a Article in Nature Structural & Molecular Biology.

Acceptance is conditional on the manuscript's not being published elsewhere and on there being no announcement of this work to the newspapers, magazines, radio or television until the publication date in Nature Structural & Molecular Biology.

Over the next few weeks, your paper will be copyedited to ensure that it conforms to Nature Structural & Molecular Biology style. Once your paper is typeset, you will receive an email with a link to choose the appropriate publishing options for your paper and our Author Services team will be in touch regarding any additional information that may be required.

After the grant of rights is completed, you will receive a link to your electronic proof via email with a request to make any corrections within 48 hours. If, when you receive your proof, you cannot meet

this deadline, please inform us at rjsproduction@springernature.com immediately.

You will not receive your proofs until the publishing agreement has been received through our system.

Due to the importance of these deadlines, we ask that you please let us know now whether you will be difficult to contact over the next month. If this is the case, we ask you provide us with the contact information (email, phone and fax) of someone who will be able to check the proofs on your behalf, and who will be available to address any last-minute problems.

To assist our authors in disseminating their research to the broader community, our SharedIt initiative provides all co-authors with the ability to generate a unique shareable link that will allow anyone (with or without a subscription) to read the published article. Recipients of the link with a subscription will also be able to download and print the PDF.

As soon as your article is published, you can generate your shareable link by entering the DOI of your article here: <a href="http://authors.springernature.com/share">http://authors.springernature.com/share<a>. Corresponding authors will also receive an automated email with the shareable link

Note the policy of the journal on data deposition: http://www.nature.com/authors/policies/availability.html.

Your paper will be published online soon after we receive proof corrections and will appear in print in the next available issue. You can find out your date of online publication by contacting the production team shortly after sending your proof corrections. Content is published online weekly on Mondays and Thursdays, and the embargo is set at 16:00 London time (GMT)/11:00 am US Eastern time (EST) on the day of publication. Now is the time to inform your Public Relations or Press Office about your paper, as they might be interested in promoting its publication. This will allow them time to prepare an accurate and satisfactory press release. Include your manuscript tracking number (NSMB-A45206A) and our journal name, which they will need when they contact our press office.

About one week before your paper is published online, we shall be distributing a press release to news organizations worldwide, which may very well include details of your work. We are happy for your institution or funding agency to prepare its own press release, but it must mention the embargo date and Nature Structural & Molecular Biology. If you or your Press Office have any enquiries in the meantime, please contact press@nature.com.

You can now use a single sign-on for all your accounts, view the status of all your manuscript submissions and reviews, access usage statistics for your published articles and download a record of your refereeing activity for the Nature journals.

If you have not already done so, we strongly recommend that you upload the step-by-step protocols used in this manuscript to the Protocol Exchange. Protocol Exchange is an open online resource that allows researchers to share their detailed experimental know-how. All uploaded protocols are made freely available, assigned DOIs for ease of citation and fully searchable through nature.com. Protocols can be linked to any publications in which they are used and will be linked to from your article. You can also establish a dedicated page to collect all your lab Protocols. By uploading your Protocols to Protocol Exchange, you are enabling researchers to more readily reproduce or adapt the methodology

you use, as well as increasing the visibility of your protocols and papers. Upload your Protocols at www.nature.com/protocolexchange/. Further information can be found at www.nature.com/protocolexchange/about.

An online order form for reprints of your paper is available at https://www.nature.com/reprints/author-reprints.html. Please let your coauthors and your institutions' public affairs office know that they are also welcome to order reprints by this method.

Please note that <i>Nature Structural & Molecular Biology</i> is a Transformative Journal (TJ). Authors may publish their research with us through the traditional subscription access route or make their paper immediately open access through payment of an article-processing charge (APC). Authors will not be required to make a final decision about access to their article until it has been accepted. Find out more about Transformative Journals

<B>Authors may need to take specific actions to achieve compliance with funder and institutional open access mandates. For submissions from January 2021, if your research is supported by a funder that requires immediate open access (e.g. according to Plan S principles) then you should select the gold OA route, and we will direct you to the compliant route where possible. For authors selecting the subscription publication route our standard licensing terms will need to be accepted, including our self-archiving policies. Those standard licensing terms will supersede any other terms that the author or any third party may assert apply to any version of the manuscript.

In approximately 10 business days you will receive an email with a link to choose the appropriate publishing options for your paper and our Author Services team will be in touch regarding any additional information that may be required.

You will not receive your proofs until the publishing agreement has been received through our system.

If you have any questions about our publishing options, costs, Open Access requirements, or our legal forms, please contact ASJournals@springernature.com

Sincerely,

Carolina

Carolina Perdigoto, PhD
Chief Editor
Nature Structural & Molecular Biology
orcid.org/0000-0002-5783-7106
